# Biodegradation of Xenoestrogens by the Green Tide Forming Seaweed *Ulva*: A Model System for Bioremediation

**DOI:** 10.1021/acsestwater.4c00961

**Published:** 2025-03-05

**Authors:** Justus B. Hardegen, Maximilian S. F. Knips, Johanna K. Däumer, Svenja Kretzer, Thomas Wichard

**Affiliations:** Institute for Inorganic and Analytical Chemistry, Friedrich Schiller University Jena, Jena 07743, Germany

**Keywords:** alga, bisphenol, bromoperoxidase, degradation, ethinylestradiol, micropollutants, sea lettuce, transformation products

## Abstract

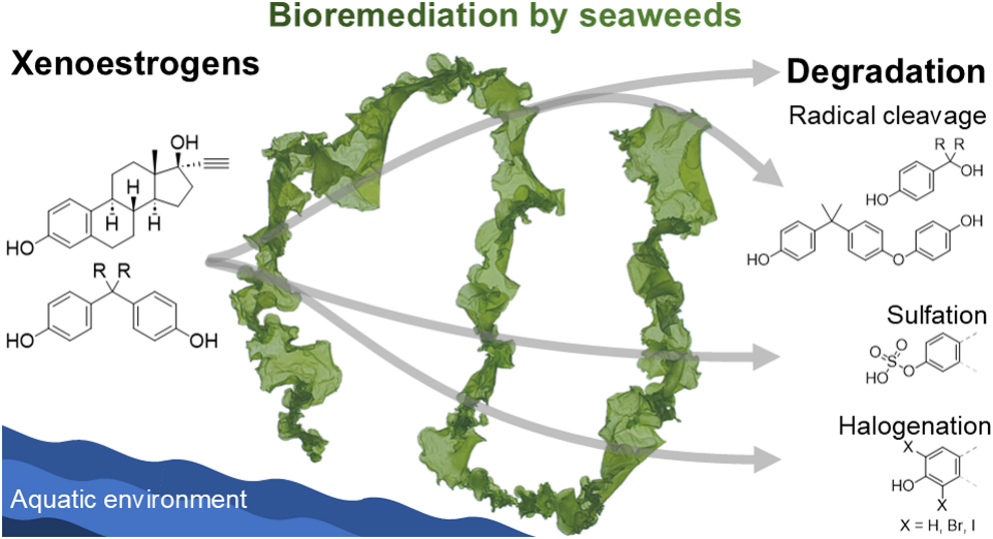

Anthropogenic xenoestrogens pose serious threats to humans and the environment. *Ulva* (Chlorophyta), a green macroalga that can propagate in environments of various salinities, is a potential candidate for efficient wastewater treatment and bioremediation. In this study, we tested the class of bisphenols and ethinylestradiol and investigated the underlying removal mechanisms of these xenoestrogens. The model organism *Ulva mutabilis* demonstrated over 99% removal efficiency for bisphenols A, B, E, F, P, and Z, and partial removal of bisphenol S. *Ulva* showed complete removal capabilities even under axenic conditions, while its associated bacteria were not involved. Complete removal of 6.6 mg L^–1^ of bisphenol A was achieved within 2 days and a half-time of 1.85 h. Biodegradation was the leading cause of removal, whereas bioaccumulation was minimal. The model substance bisphenol A underwent various reactions, and 20 transformation products were detected using stable isotope labeling. While most of the bisphenol A was completely biodegraded, the primary transformation products were monobromobisphenol A, bisphenol A bisulfate, and 4-hydroxypropanylphenol. This study highlights the potential of the green seaweed *Ulva* to provide a pathway for more effective and sustainable bioremediation strategies to tackle the environmental pollution caused by xenoestrogens.

## Abstract 2

We provide strong evidence for the viability of green seaweed as a bioremediation tool. Algae, particularly *Ulva*, have a significant potential to mitigate the effects of xenoestrogens, such as bisphenols, in contaminated water systems.

## Introduction

Anthropogenic activity has profound and detrimental effects on ecosystems worldwide. Contaminants of emerging concern, or micropollutants (MP), have become a focus of interest because of their potential to affect the environment and human health at very low concentrations.^1−3^ In particular, even minute concentrations of xenoestrogens–a class of endocrine-disrupting chemicals that mimic the activity of the natural estrogens–can affect animals and humans.^4,5^ While their long-term effects at low concentrations are often subtle and difficult to verify, they have been shown to cause metabolic and developmental disorders and reduce fertility.^6−8^ Therefore, environmental pollution caused by xenoestrogens must be prevented by avoiding emissions and eliminating the emitted xenoestrogens.^9,10^ Bisphenol A (BPA) is the most prominent xenoestrogen because it is present in several everyday objects, resulting in a constant and worldwide release.^11^ This has led to the manufacturing of BPA-free products and a ban on BPA for thermal paper (e.g., paper receipts) in the European Union.^12,13^ However, BPA is often simply replaced with other bisphenols such as bisphenol F (BPF) or bisphenol S (BPS), which have been shown to exert similar endocrine-disrupting properties.^14,15^ Another relevant xenoestrogen is α-ethinylestradiol (EE_2_), a synthetic estrogen used in oral contraceptives and menopausal therapy. After its intended use, EE_2_ is excreted through the urine and enters wastewater systems. However, conventional water treatment processes often fail to sufficiently remove EE_2_, thereby allowing it to enter the environment.^16^

Although numerous researchers have studied the removal of xenoestrogens, especially BPA, by bacteria and physicochemical processes, further research is required to understand the adverse effects of xenoestrogens on the aquatic environment and their removal by aquatic organisms.^17,18^ For example, algae offer an economical, adaptable, and eco-friendly tool for wastewater treatment and bioremediation.^19−21^ Macroalgae are highly effective owing to their ease of harvest, capacity to be attached for coastal bioremediation, and ability to be retained in cleaning systems or integrated multitrophic aquacultures.^22−25^ However, their potential remains insufficiently explored, and the contribution of the associated symbiotic bacteria is unclear because the degradation is often generally attributed to algae.^5^

The green macroalga *Ulva*, commonly known as sea lettuce, is a promising candidate for wastewater management programs in coastal, estuarian, and urban areas because of its wide tolerance to salinity and capability to remove nutrients and heavy metals.^22,26−29^ Additionally, *Ulva* has been reported to remove BPA and EE_2_ from its surrounding environment.^30,31^ The cosmopolitan alga *Ulva compressa* (cultivar *mutabilis*) has been developed into a well-suited model organism for research on, for example, environmental stresses on algae.^32^

**Figure 1:**
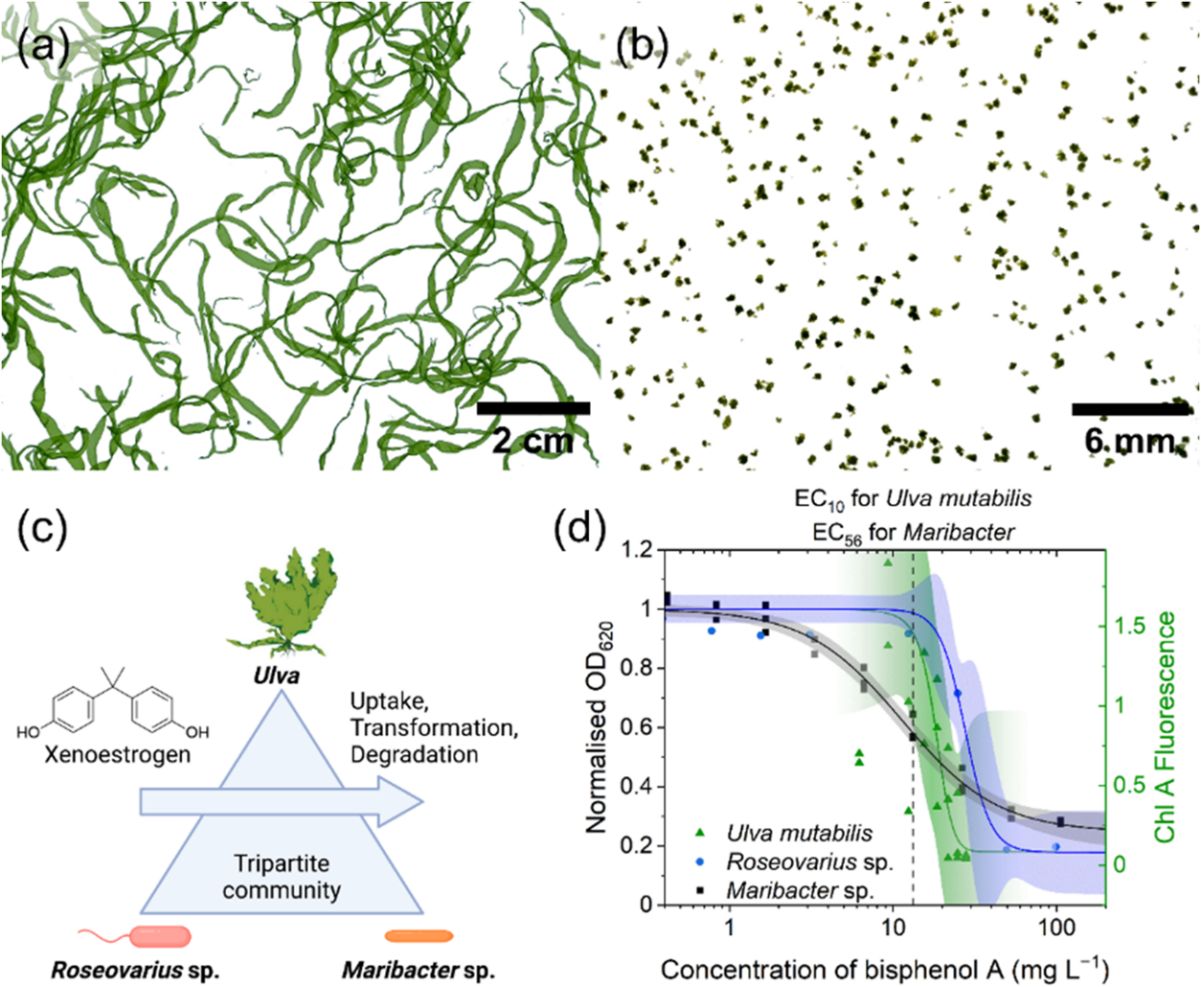
The tripartite community of *Ulva mutabilis***–***Roseovarius* sp.–*Maribacter* sp. in the presence of BPA. Photographs of the tubular *U. mutabilis* inoculated with the two essential bacteria releasing AGMPFs (a), *U. mutabilis* cultivated under axenic conditions without the presence of AGMPFs (b), feasible removal mechanisms of BPA (c) New Text (created with BioRender.com. Wichard, T. (2025) https://BioRender.com/q04e174), and toxicity toward the individual species of the tripartite community (d).

Some key features are highlighted here that are essential for this study:^33^*Ulva* live in symbiosis with a diverse bacterial consortium but can also be cultivated under axenic and standardized conditions.^34^ The complex microbiome of *Ulva mutabilis* can be reduced to two bacterial strains, *Roseovarius* sp. and *Maribacter* sp., which release algal growth and morphogenesis-promoting factors (AGMPF) essential for full algal development (Figure 1a).^35^ This tripartite community of *U. mutabilis*–*Roseovarius* sp.–*Maribacter* sp. is stable in culture, and the species can be separated and grown individually. *U. mutabilis* develops into deformed cell clusters under axenic conditions (callus, Figure 1b).^36^ Alternatively, axenic *U. mutabilis* can be supplemented with AGMPFs that facilitate species-typical development under axenic conditions (recovered morphotype).^33^ This allows the investigation of individual species of the holobiont under controlled laboratory conditions and paves the way to address the following research objectives of this study:

(i) The ecotoxicological effect of xenoestrogens on the development of the holobiont *Ulva*, particularly in interactions with its effective morphogens.
(ii) The contribution of algae and bacteria to micropollutant bioremediation.

However, the process of xenoestrogen removal by *Ulva* is not yet fully understood. Zhang et al. (2019, 2021) investigated the removal of BPA from *U. prolifera* and *U. pertusa*.^30,37^ While both species removed BPA efficiently, *U*. *pertusa* displayed adsorption potential and a higher removal rate.^37^ In both studies, the authors concluded that BPA is metabolized after uptake because the majority of the removed BPA could not be extracted from the algal tissue. However, the current research lacks a temporal profile of the absorbed BPA to prove that the missing amount is not an artifact caused by low recovery. Furthermore, no BPA transformation products or degradation intermediates by *Ulva* have been reported thus far, making it unclear what occurs to the BPA after uptake.^18,38,39^ Therefore, further research is required to understand the mechanisms underlying algae-mediated xenoestrogen removal. Previous studies lacked controlled axenic conditions and could not assess the contribution of the algal microbiome; however, the *Ulva* model system enabled studies to be conducted under fully axenic conditions. To our knowledge, this is the first study that used controlled axenic conditions to distinguish between the species involved and investigate their contributions.

Phytoremediation, a field with immense potential for tackling environmental pollution, particularly with specific toxins such as xenoestrogens, requires thorough laboratory investigations in controlled settings to identify specific algae- or bacteria-micropollutant interactions. This study examined how *U. mutabilis*, a green macroalga, can remove bisphenols and α-ethinylestradiol. Experimental dissection of the host and microorganisms using gnotobiotic (axenic) algae helped identify the species contribution to BPA removal and calculate the uptake and removal kinetics (Figure 1c). Stable isotope labeling and high-resolution mass spectrometry were performed to identify the transformation products of BPA, BPF, and EE_2_.

## Materials and Methods

### Algae and Bacteria

Haploid gametophytes of *Ulva mutabilis* Føyn (sl-G[mt + ]; morphotype ’slender’; *locus typicus*: Ria Formosa, Portugal, strain FSU-UM5–1) were used as a model system for the green macroalga. The cultivar is conspecific to *Ulva compressa* and is referred to as *Ulva mutabilis* throughout the study.^40^ Axenic gametes were inoculated with two bacterial strains, *Roseovarius* sp. strain MS2 (GenBank EU359909) and *Maribacter* sp. strain MS6 (GenBank EU359911) to obtain a tripartite community.^33^ Alternatively, the two bacterial strains were substituted with their produced algal growth and morphogenesis-promoting factors (AGMPFs), specifically the *Maribacter*-factor thallusin (10 nmol L^–1^) and an extract from the *Roseovarius* sp. supernatant. This *Ulva* culture typically matures into adult algae under axenic conditions.^43,45^

### Standard Cultivation Procedure for Algae and Bacteria

Algal cultures were cultivated, and all experiments with algae were performed in artificial seawater *Ulva* culture medium (UCM) at 18 °C ± 2 °C with a light/dark cycle of 17/7 h and a light intensity of 40–80 μmol photons m^–2^ s^–1^.^35,41^ Bacteria strains were propagated in marine broth-enriched UCM (50% by volume; Carl Roth, Germany) and investigated in the same medium or their respective minimal medium: UCM enriched with 1% by volume glycerol as a carbon source for *Roseovarius* sp. MS2 and HaHa_100 medium for *Maribacter* sp. MS6.^42,43^

### Dose–Response Curves for *Roseovarius* sp. and *Maribacter* sp. Inoculated with BPA

The effect of BPA on the growth of the tripartite community was investigated with a broth microdilution method and minimal media in 48 well plates (*V* = 500 μL).^44^ The growth was regularly measured using the multimode microplate reader Varioskan Flash (Thermo Fisher Scientific, Waltham, MA) at an optical density of 620 nm in technical triplicates with a starting optical density of 0.02. The late exponential phase (5 d for *Roseovarius* sp. MS2, and 20 h for *Maribacter* sp. MS6) was used to calculate the dose–response curves. The curves were calculated as previously described.^31^

### Removal of Bisphenol by the Tripartite Community *U. mutabilis*–*Roseovarius* sp.–*Maribacter* sp. from the Culture Medium

The removal of bisphenols A, B, E, F, P, S, and Z and ethinylestradiol by the tripartite community was investigated according to established protocols.^31^ Three to five individual 4-week-old (1–2 cm) tripartite *Ulva* cultures were incubated with concentrations based on the 10% effect (EC_10_) calculated for *Ulva* in toxicity assays for BPE, BPF, and BPZ (Figure S3, Table S6). The concentrations of BPA, BPB, BPP, BPS, and EE_2_ varied because of their poor solubility and lack of or high variation in toxicity. *Ulva* was incubated with 6.62 mg L^–1^ (29.0 μmol L^–1^) BPA, 11.3 mg L^–1^ (46.4 μmol L^–1^) BPB, 24.1 mg L^–1^ (113 μmol L^–1^) BPE, 3.19 mg L^–1^ (15.9 μmol L^–1^) BPF, 2.50 mg L^–1^ (7.22 μmol L^–1^) BPP, 96.8 mg L^–1^ (387 μmol L^–1^) BPS, 10.7 mg L^–1^ (39.8 μmol L^–1^) BPZ, and 2.83 mg L^–1^ (9.53 μmol L^–1^) EE_2_ in biological triplicates. Medium samples were taken before, 1 h after, and 14 days after adding the alga to the dissolved xenoestrogens. Abiotic controls with and without light were obtained before and 14 d after incubation.

### Removal of BPA by Single Species of the Tripartite Community *U. mutabilis* – *Roseovarius* sp.–*Maribacter* sp

The change in BPA concentration over 14 days starting with 13.2 mg L^–1^ (58.0 μmol L^–1^) was determined for different phenocopies of *U. mutabilis*: tripartite *Ulva* (Figure 1a), axenic callus-shaped *Ulva* (Figure 1b), and axenic *Ulva* fully developed by supplementation of AGMPFs in UCM.^45^ The changes in BPA were also determined for the two bacterial strains of the tripartite community in complex and minimal media. Biological triplicates and abiotic controls were prepared for all conditions.

### Amount of BPA, BPF, and Ethinylestradiol in Medium and Alga Tissue during Removal

Three to five individuals of 4-week-old (1–2 cm) tripartite *Ulva* cultures were incubated with 13.2 mg L^–1^ (58.0 μmol L^–1^) BPA, 3.18 mg L^–1^ (15.9 μmol L^–1^) BPF, or 2.83 mg L^–1^ (9.53 μmol L^–1^) EE_2_ in 2 mL of UCM. In total 0.62 mg BPA, 0.18 mg BPF, and 0.16 mg EE_2_ per 1 ± 0.1 g *Ulva* (fresh weight) were added to the culture. Medium and algal tissue samples were collected immediately and at doubling intervals between 22.5 min and 16 d after addition. In total, 36 biological replicates were prepared for each compound, and three replicates were sacrificed at each sampling point. Additionally, medium samples of abiotic controls were obtained.

### Quantification of Bisphenols and Ethinylestradiol in Aqueous Samples

The concentrations of all xenoestrogens in the water were determined using ultrahigh performance liquid chromatography-electrospray ionization-high-resolution mass spectrometry (UHPLC-ESI-HRMS), as reported in our previous publication (see Supporting Information for details, Figure S1, Tables S1−S3).^31^ Briefly, samples were spiked with stable isotope-labeled internal standards, diluted, and filtered before injection.

### Quantification of Bisphenols and Ethinylestradiol in Algal Tissue

Xenoestrogens in the algal tissue were quantified based on established protocols (see Supporting Information for details, Figure S2, Tables S4,S5).^46,47^ Briefly, algal samples were washed with ultrapure water, freeze-dried, pulverized, and extracted. The extraction mixture contained ethanol, methanol, chloroform (60:20:20 by volume), and isotopologous internal standards (1% of the initial molar amount). After centrifugation, the supernatant was collected, the solvents were evaporated under vacuum, and the residue was resuspended in pyridine, derivatized with MSTFA, and measured using gas chromatography (GC)-EI-HRMS.

### Identification of Transformation Products by Stable Isotope Labeling

BPA, BPF, and EE_2_ transformation and degradation products were detected in an isotope label data set comparison experiment (see Supporting Information for details).^48^ For this purpose, each compound and at least one stable isotope-labeled isotopologue were incubated in parallel with 100 mg of tripartite *Ulva mutabilis* (fresh weight) in 2 mL UCM. We tested 13.2 mg L^–1^ BPA (58.0 μmol L^–1^), 14.2 mg L^–1^ BPA-D_16_ (58.0 μmol L^–1^), 13.9 mg L^–1^ BPA-^13^C_12_ (58.0 μmol L^–1^), 3.18 mg L^–1^ BPF (15.9 μmol L^–1^), 3.34 mg L^–1^ BPF-D_10_ (15.9 μmol L^–1^), 2.83 mg L^–1^ EE_2_ (9.53 μmol L^–1^), and 2.86 mg L^–1^ EE_2_-D_4_ (9.53 μmol L^–1^). Five biological replicates were prepared for each substance, and controls were prepared without xenoestrogen. After 2 days, algal tissue samples were collected and extracted for GC and LC metabolomic analyses. Mass spectrometric data were analyzed using the R package X^13^CMS to identify features of labeled transformation products.^49,50^ Identified features were manually verified as coeluting and deconvoluted into mass spectra. The structures of the transformation products were elucidated using stable isotope labeling, knowledge of precursor molecules, high-resolution mass spectrometry, and customary techniques (see Supporting Information for details, Figure S8).^51^ After identifying major pathways, the data were re-evaluated and checked for traces of further transformation products of the identified reactions.

The identities of 3-monobromobisphenol A, 3,3′-dibromobisphenol A, 3,3′,5-tribromobisphenol A, and 3,3′,5,5′-tetrabromobisphenol A were confirmed by coinjection with standards synthesized according to Doumas et al. (2018) using bromine as the reagent and CH_2_Cl_2_/THF as the solvent.^52^ Coinjection with a commercial standard confirmed the identity of 4-(2-hydroxypropan-2-yl)phenol. MS^2^ experiments with a collision energy of 55 eV confirmed the structure of BPA bisulfate.

### Repeated Addition of BPA to an Algal Culture

The 6-week-old tripartite community *U. mutabilis*–*Roseovarius* sp.–*Maribacter* sp. was incubated at BPA concentrations found in contaminated wastewater to determine its feasibility for bioremediation. Five cultures of 1.3 g fresh *Ulva* in 115 mL UCM were spiked with 228 μg L^–1^ (≙ 1 μmol L^–1^) every 12 h for 3 days. Culture medium samples were collected before and after spiking and 6 h after spiking during the day.

## Results and Discussion

### *Ulva* removes BPA and Supports Bacteria Growth

Algae, with their unique ability to create protective microenvironments, shield bacteria from harmful conditions, such as UV radiation, temperature fluctuations, and desiccation. The extracellular polymeric substances produced by macroalgae form biofilms that serve as safe harbors for bacteria. We tested the hypothesis that *U. mutabilis* can absorb and detoxify harmful bisphenols in the environment. The algal growth-promoting and thallusin-releasing strain *Maribacter* sp. was found to be even more sensitive to BPA than the algal host itself, with its growth inhibited by 56% at concentrations that decreased Chl-*a* fluorescence of *Ulva* only by 10% (Figure 1d). Additionally, *Maribacter* sp. was affected at lower concentrations than *U. mutabilis* and *Roseovarius* sp. While the other effective concentration (EC) curves were steep, *Maribacter* sp. displayed a flat slope over 2 orders of magnitude with an increasing effect.

### Bisphenols are Eliminated in the Presence of the Green Seaweed *Ulva*

**Figure 2:**
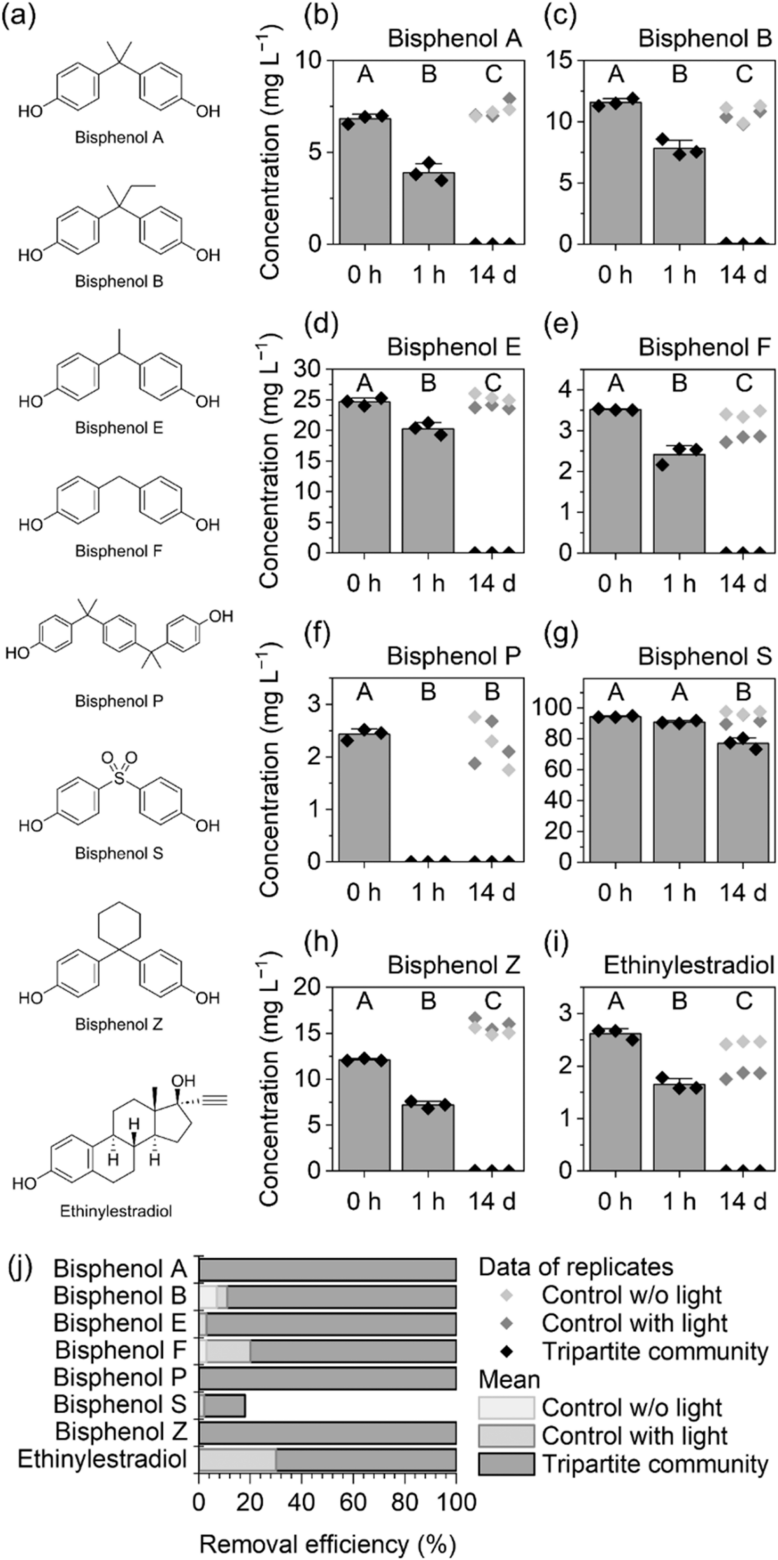
Removal of xenoestrogens from the culture medium. Structures of tested substances (a). Concentrations in the culture medium before and after incubation with the 4-week-old tripartite community: *Ulva mutabilis*–*Roseovarius* sp.–*Maribacter* sp. (b–i). Data of replicates are depicted as symbols, and the means and standard deviations of replicates with the tripartite community as columns with whiskers. Means with different capital letters differ significantly (*p* ≤ 0.001, ANOVA with posthoc Dunn-Šidák). Removal efficiency under different conditions (j).

The tripartite community of *Ulva* swiftly removed bisphenols B, E, F, P, and Z, in addition to BPA and EE_2_ (Figure 2). A significant amount of these bisphenols was eliminated within 1 h (18 to 100%), with complete elimination over 14 days. BPP was the most efficiently eliminated, falling below the limit of detection (LOD) within 1 h. BPS was the only exception, showing a reduction of only 18% within 14 days. (Figure 2g). EE_2_ and BPF exhibited reduced stability in the abiotic controls under light, but their removal was significantly accelerated in the presence of the tripartite community.

We postulate that similar mechanisms are responsible for the removal of all the bisphenols. While BPP was particularly susceptible to this mechanism and BPS remained relatively stable, the bisphenols A, B, E, F, and Z behaved similarly, which is likely a consequence of their closely related structure. The different linking group of BPS (sulfone) changes its polarity and activity compared to other bisphenols, which are all linked via hydrocarbons.^53^ For example, if the hypothetical removal mechanism depends on the diffusion or transport of MPs through the cell membrane, the higher polarity of BPS may hinder removal.^54^ Based on these results, we do not recommend the replacement of BPA with BPS, as the latter proved considerably more persistent under the studied conditions, whereas it is reported to exert endocrine-disrupting properties similar to BPA or BPF.^14,15^

### BPA was Removed by the Green Seaweed *Ulva* without Bacterial Support

**Figure 3:**
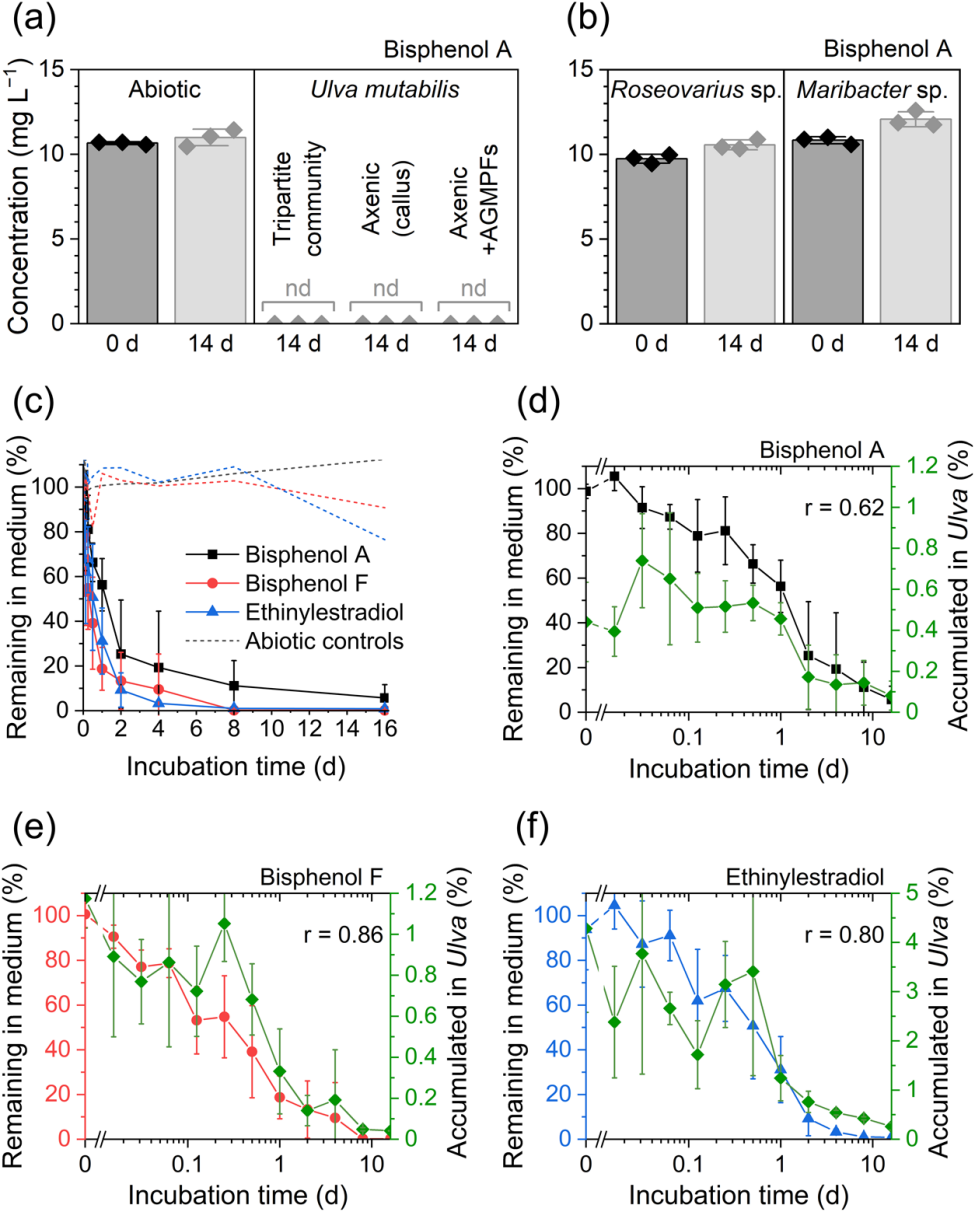
Concentrations of BPA in the culture medium before and after incubation with the tripartite community or axenic culture of *Ulva mutabilis*. Axenic *Ulva* was tested as a callus or with complete morphogenesis by adding AGMPFs and compared with abiotic conditions (a). BPA concentrations were determined in minimal medium before and after inoculation with *Roseovarius* sp. MS2 or *Maribacter* sp. MS6 (b). The concentrations of replicates are depicted as symbols and the means as columns with standard deviations as whiskers (a, b). Percentages of BPA (black), bisphenol F (red), and ethinylestradiol (blue) remaining in the medium and accumulated in *Ulva* tissue (green) over 16 days (c−f). BPA percentages in tissue were significantly lower and were correlated with their percentages in the culture medium (Spearman’s rank correlation coefficient [r]). Symbols represent the means of biological replicates, with whiskers indicating the standard deviation.

Axenic *U. mutabilis* and both bacterial strains of the tripartite community were individually incubated with BPA to determine the species-dependent contribution to BPA removal within the tripartite community. BPA was removed below the LOD under axenic conditions with and without the addition of bacterial AGMPFs (Figure 3a). Notably, the two bacterial strains did not significantly decrease the concentration of BPA in any of the culture media tested (Figure 3b). *U. mutabilis* was responsible for the observed decrease in BPA levels, whereas bacterial strains made no direct or indirect contributions (through growth and morphogenesis stimulation). Remarkably, BPA even inhibited the growth of *Maribacter* sp. MS6 by 56% at the tested concentrations (Figure 1d). Consequently, *U. mutabilis* may safeguard its supply of thallusin by removing BPA and enabling the unrestrained growth of the thallusin producer *Maribacter*. Further studies are required to investigate these interactions.

### Removal Kinetics

The removal kinetics of the three most relevant xenoestrogens—BPA, BPF, and EE_2_—were observed over 16 days. The concentration of each tested compound in the culture medium declined from 100% of the original concentration to 0%, reflecting the typical curve of a first-order reaction with a rapid change at first and slowing down over time (Figure 3c). The removal of BPF and EE_2_ was more rapid than BPA with half-times of 10, 14, and 30 h, respectively. To improve the conditions for bioremediation, the amount of *Ulva* biomass was increased 3-fold, and the starting concentration was halved. Consequently, the half-time of BPA removal was reduced to 1.85 h with a starting concentration of 6.62 mg L^–1^ (Figure S4). Furthermore, throughout all experiments, the speed of pollutant removal was considerably higher when the algae were in the active growth phase compared to those in stationary cultures. Nevertheless, even in total darkness, *U. mutabilis* eliminated BPA, although the rate of BPA removal was significantly decreased (Figure S4).

### Identification of Algal Xenoestrogens Removal Mechanism

To determine the uptake effect, the amounts of bioaccumulated BPA, BPF, and EE_2_ in *Ulva* were compared with the amounts remaining in the culture medium over 16 d (Figure 3d–f). The accumulated amount was significantly smaller (paired *t* test, *p* < 0.0001), with percentages of the initially added amount below 1% for BPA and BPF and below 5% for EE_2_ and followed a similar course with significant correlation (Spearman’s rank, *p* < 0.001). The significant correlation between the xenoestrogens remaining in the medium and tissue accumulation suggests an equilibrium between these states. This equilibrium could be the result of a passive process, such as diffusion through the cell membrane, owing to the low bioaccumulation factor. Given that a removal mechanism based solely on the uptake or adsorption would result in an increase in the amount of accumulated substances in *Ulva* tissue as that in the medium reduces, these findings indicate a removal mechanism involving uptake followed by chemical transformation.

The possibility of abiotic removal and loss during the washing of *Ulva* tissue was adequately addressed and eliminated by implementing appropriate controls. Furthermore, the abiotic control, which contained cell-free spent medium from *Ulva*, did not remove xenoestrogens (Figure 3a), indicating that extracellular enzyme activity was unlikely. Therefore, the only possibility is that *Ulva* takes up xenoestrogens and alters them chemically within its tissues. This process may involve chemical modifications (biotransformation) or degradation. In comparison, autoclaved *Ulva* partially removed BPA by adsorption but was over 100 times slower than living *Ulva*, confirming an active biological process as a major contributing factor to the removal (Figure S4).

### Algal Transformation of BPA

**Figure 4:**
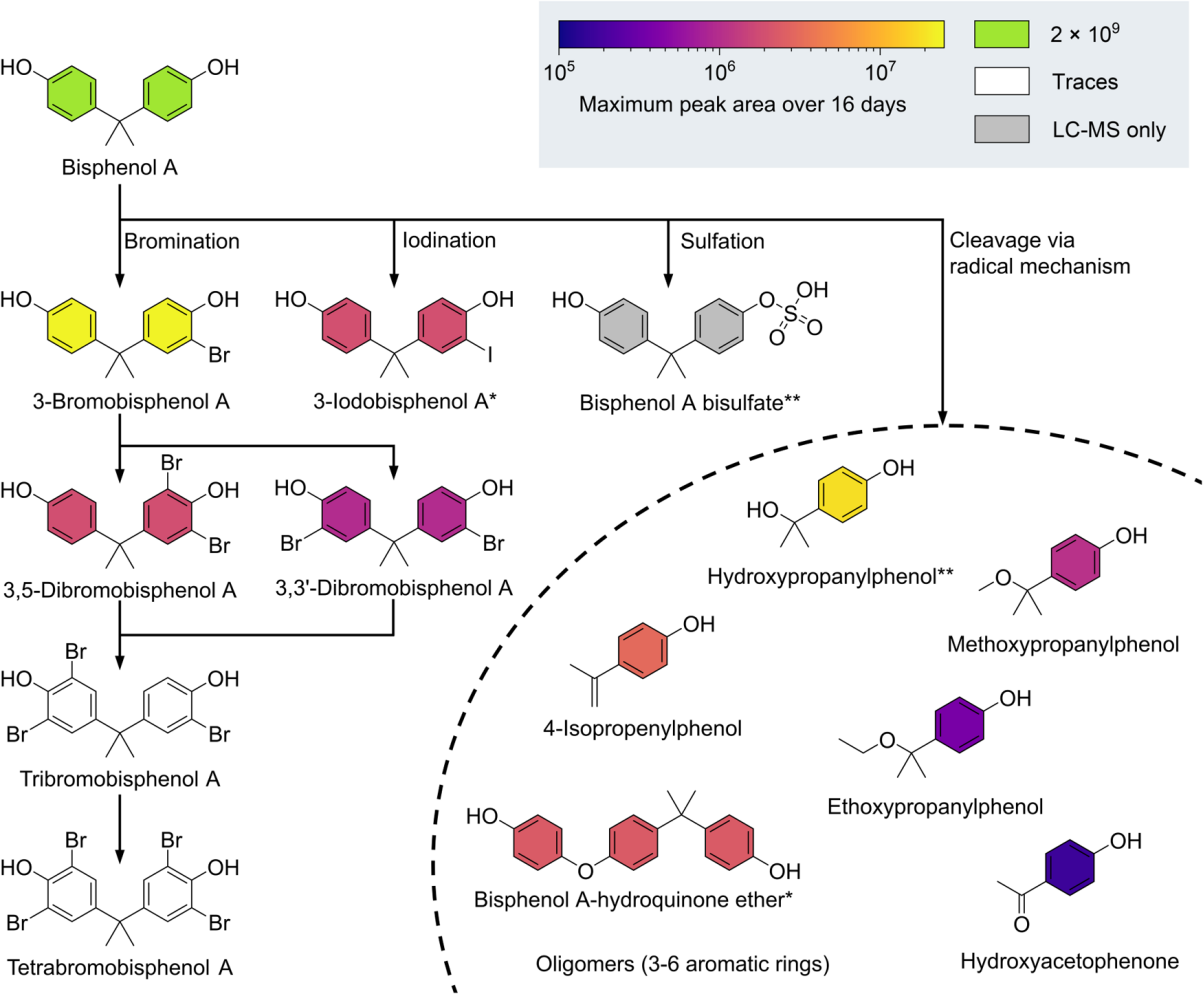
Transformation and degradation products of BPA identified in the tripartite community of *Ulva mutabilis***–***Roseovarius* sp.–*Maribacter* sp. Besides bisulfates (LC-MS), all transformation products were detected via GC-MS in *Ulva*. Additional brominated analogs were detected: * monobrominated, ** mono- and dibrominated. For GC-MS analysis, the maximum peak area of the products was determined over 16 days upon the onset of the experiment. A color code displays the maximum peak areas (See Supporting Information for details, Figure S5).

Twenty BPA transformation products were identified in *Ulva*. Of these, 15 were initially flagged as labeled via X^13^CMS, and traces of five more were detected using a targeted search (see Figures S9–S33 and Table S9). The transformation products underwent four primary reaction types: bromination, iodination, sulfation, and cleavage via a radical mechanism (Figure 4). Bromination was the most prominent transformation and resulted in five possible analogs of BPA brominated at the ortho position of the phenols: 3-monobromobisphenol A, 3,5-dibromobisphenol A, 3,3′-dibromobisphenol A, 3,3′,5-tribromobisphenol A, and 3,3′,5,5′-tetrabromobisphenol A. Additionally, seven brominated analogs from other pathways were identified (see below). The second type of reaction involves iodination with 3-iodobisphenol A and two of its monobromated isomers identified. Bromination and iodation are very likely to be catalyzed by bromoperoxidases reported to be present in *Ulva* spp.^55−57^ Bromination of bisphenol A up to four times was previously reported for the red seaweed *Gracilaria* sp.^58^ Although haloperoxidases are considerably common in the aquatic environment, this is the first known report on an iodized transformation product of BPA enabled by the sensitivity of the isotope-guided approach and the high tolerance of *Ulva* toward BPA.^18,38,39,59^ Notably, the enzymatic halogenation of the present bromoperoxidase seems to favor the formation of the 3,5-isomer of dibromo BPA because the peak area of the chromatogram was significantly higher than of the 3,3′-isomer (see Figure S24). Thus, the average proportion during incubation was 66.7% (1–16 days, *p* = 0.62%). In comparison to the proposed bromoperoxidase activity, the organic synthesis of dibromo BPA via electrophilic aromatic substitution is very selective toward the 3,3′-isomer and yields no 3,5-isomer.^52^ This result suggests the presence of a substrate binding site and supports recent discussions regarding the regioselectivity of bromoperoxidases, which generate free HOBr and have therefore, historically been thought to be nonselective.^60,61^ However, recent studies have found selective bromoperoxidases in bacteria.^62,63^ Moreover, one study on fungal haloperoxidase found evidence of a substrate-specific binding site close to the active site and a tunnel connection able to guide the active HOX to the substrate.^64^ Further investigation of bromoperoxidases of *Ulva* might provide novel insights into their catalytic activity, and bromoperoxidases in *Ulva* spp. may be promising tools for biocatalysis.^65^

The third type of reaction is sulfation, which forms BPA bisulfate and at least two further brominated analogs, also found in the culture medium (Figures S6,S7). Sulfotransferases are the most probable catalysts for this reaction, and *U. mutabilis* possesses multiple annotated genes for these enzymes.^66^ While BPA bisulfate is a known transformation product in mammals, to the best of our knowledge, this has not yet been reported in algae.^11,18,38,39^

The fourth reaction type was diverse: BPA cleavage resulted in five degradation products (propenylphenol (PP), methoxypropanylphenol, (MPP), ethoxypropanylphenol (EPP), hydroxypropanylphenol (HPP), and hydroxyacetophenone (HAP)) as well as two brominated analogs of the most abundant degradation product (bromohydroxypropanylphenol (BrHPP) and dibromohydroxypropanylphenol (Br_2_HPP)). These chemicals are structurally similar and may be formed by the same mechanism. Two additional transformation products contributed to the identification of the relevant pathway: the self-coupling product BPA-hydroquinone ether and one brominated analog (both with higher labeling than the original BPA, see Figures S29,S30), indicating a radical mechanism previously observed for peroxidases from plants and microorganisms.^67,68^ This hypothesis is supported by the detection of several other higher-labeled compounds in LC samples (^13^C_18_, ^13^C_24_, ^13^C_30_, ^13^C_36_, see Supporting Information for details, Table S9). Although these compounds have not yet been characterized, we propose they are coupling products of the same radical process with three to six tagged aromatic rings as described for similar products.^67^ Although the cleavage products propenylphenol and hydroxypropanylphenol were previously observed in the green alga *Desmodesmus* and were attributed to oxidative cleavage, this pathway does not account for the coupling products.^69^

The estimated quantity of all the transformation products was at least 100 times lower than that of the initially added BPA (see Figures S5−S7). The absence of substantial transformation products indicates that radical cleavage degradation was the primary mechanism for BPA removal.

Because the abiotic and bacterial controls displayed no significant removal of BPA in the medium, the detected radical cleavage products were most likely generated by *Ulva*. Furthermore, products of the other pathways were detected in the supernatant of axenic *Ulva* treated with BPA but were absent in abiotic controls (Figure S7). Therefore, *Ulva* generated all detected transformation products.

Under complete darkness, the removal rate was approximately eight times lower, demonstrating that the primary removal mechanism was light-dependent (Figure S4). This is consistent with a radical photodegradation process where *Ulva* acts as a photosensitizer.^70^ Therefore, we propose that radical cleavage was catalyzed by an oxidoreductase (e.g., peroxidase) that transfers one electron from BPA (or other phenols) to an electron acceptor (e.g., hydrogen peroxide), which was generated under irradiation of *Ulva*.

Although the removal rate decreased in the absence of light, the quantity of brominated and sulfated products in the culture medium increased considerably, providing evidence that the removal was primarily caused by the radical cleavage pathway, which slowed down in darkness, leaving more BPA available for bromination and sulfation as well as not degrading brominated and sulfated products (Figure S7). Considering that iodination occurs as a side reaction of bromoperoxidase activity and that the measured peak areas of the iodized products were negligible, this reaction was unlikely to be the main cause of quantitative removal.

### Algal Transformation of BPF

Four BPF degradation products were identified: ethoxymethylphenol, hydroxybenzaldehyde, hydroxymethylphenol, and aminomethylphenol (see Figures S34−S37). The first three were direct analogs of the degradation products identified for BPA, and the last was presumably the product of transamination of the second. Through a targeted search, traces of sulfated BPF were observed, and no brominated or iodized products were detected. Owing to the absence of the two methyl groups in BPF compared to BPA, the potential radical intermediates in BPF are probably less stable. This instability may contribute to the accelerated radical cleavage mechanism. Indeed, the abiotic controls highlighted the lower stability of BPF than that of BPA under irradiation (See Figure 2). However, the chemistry of the phenol groups remains unaltered mainly, and we anticipate that the bromination, iodination, and sulfation reactions will remain largely unchanged. Ultimately, BPF is degraded at a higher rate than BPA, reducing the formation of brominated, iodized, and sulfated byproducts. Given its accelerated degradation and the low quantity of stable transformation products, BPF is a viable substitute for BPA.

### Algal Transformation of α-Ethinylestradiol

Determining the EE_2_ transformation products is crucial because they may retain estrogenic activity and influence the environment and human health. Traces of the two isomers of brominated EE_2_ were identified in the tripartite community using GC-MS analysis (see Figures S38,S39). The brominated products in question were previously identified in the red macroalga *Gracilaria* sp. and the freshwater green microalga *Desmodesmus subspicatus*.^58,71^ Due to the absence of transformation products, the weak signal of brominated analogs and the rapid removal observed in other experiments, EE_2_ was most likely fully metabolized by *Ulva*. However, future studies should consider standards with higher labeling levels to increase the probability of detecting various degradation products close to the detection limit.

### Bioremediation Comparison of Different Species

The bioremediation potential of *U. mutabilis* was evaluated in comparison to other species based on its BPA removal efficiency and half-life (Table 1). *U. mutabilis* demonstrated complete removal of 6.6 mg L^–1^ of BPA within 48 h (Figure S4) and was the only algal species capable of achieving full BPA removal. While one macrophyte and several bacterial strains also exhibited complete BPA removal, *U. mutabilis* had the shortest half-life (1.85 h) among all species reported in the literature, which ranged from 3.8 h to over 44 days. Although the initial BPA concentration removed by *U. mutabilis* was lower than that achieved by some other species, these concentrations are significantly higher than those typically observed in wastewater and aquatic environments. Therefore, *U. mutabilis* represents a highly promising candidate for BPA bioremediation applications.

**Table 1:** Removal of Bisphenol A by Different Species^a^

species	init. conc. (mg L^–1^)	incubation time (h)	removal eff. (%)	half-time (h)	reference
**Bacteria**					
*Acinetobacter* sp.	100	360	21	1059*	(72)
*Bacillus megaterium*	5	72	100	15**	(73)
*Bacillus* sp.	10	96	100	22.6	(74)
*Cupriavidus basilensis*	59	48	78	22*	(75)
*Cupriavidus necator*	20	168	50	23***	(76)
*Pseudomonas knackmussii*	10	168	100	56**	(77)
*Pseudomonas putida*	228	96	97	48	(78)
*Pseudomonas putida*	100	32	100	8.4**	(79)
*Pseudomonas* sp.	100	360	60	272*	(72)
*Sphingobium* sp.	100	12	100	3.8**	(80)
**Green microalgae**					
*Chlorella fusca*	18.3	168	95	39	(81)
*Desmodesmus* sp.	1	240	57	120**	(69)
*Graesiella* sp.	25	120	52	113*	(82)
*Monoraphidium braunii*	4	96	48	102	(83)
*Picocystis* sp.	25	120	72	65*	(84)
*Scenedesmus obliquus* and *Desmodesmus* sp.	17	360	95	83*	(85)
**Green macroalgae**					
*Ulva mutabilis*	6.6	48	100	1.8	this study
*Ulva pertusa*	1	36	92	9.9*	(37)
*Ulva prolifera*	0.1	24	94	5.4***	(30)
**Macrophytes**					
*Ceratophyllum demersum*	5	288	100	22**	(86)

a When multiple conditions were compared, the highest removal efficiency (Removal eff.) with corresponding initial concentration (init. conc.) and incubation time was taken. A removal efficiency of 100% represents a removal below the detection limit. Half-times were often unavailable and were instead calculated from *removal efficiency at a given incubation time, **displayed removal kinetics, or ***given reaction rate constant.

### Environmental Significance in Wastewater Treatment

**Figure 5:**
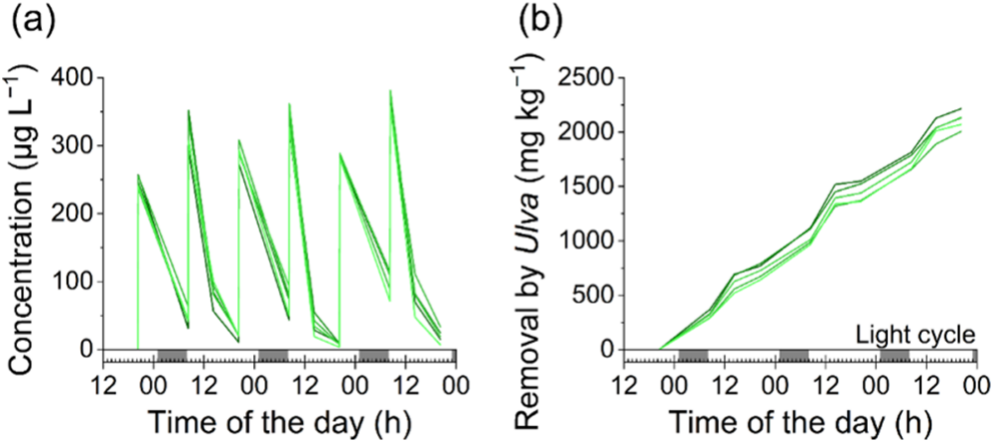
*Ulva mutabilis* consistently eliminated BPA. The concentration of BPA after 3 days of repeated spiking (a) and the total curve of eliminated BPA normalized based on the dry weight of *Ulva* are shown (b). Both curves display the outcomes of five individual repetitions, with the abscissa indicating a 14/7 light/dark cycle.

The BPA-removal capability of *Ulva* was tested at realistic concentrations found in industrial wastewater. The spiked amount was then quickly removed from the culture medium (Figure 5a). The mean removal efficiency within 12 h was 75 ± 4% over the dark period and 95.5 ± 1.3% over the light period (*N* = 5). These results highlight the importance of light during detoxification in phototrophic organisms. In total, 98.8 ± 0.7% of the added BPA was removed with an average of 2.1 mg BPA per gram dry weight (Figure 5b) or 166 μg per gram fresh weight *Ulva*.

To assess the feasibility of using *Ulva* for wastewater treatment, we calculated the necessary quantity of *Ulva* to effectively treat the wastewater produced by a paper factory of moderate size, with the objective of eliminating 99% of the BPA present. For instance, this factory generates 580 m^3^ of wastewater daily with an average concentration of 339 μg L^–1^ BPA.^87^ As 1.3 g of fresh weight was required to remove >99% BPA under laboratory conditions, we estimate that the example factory requires a cultivation tank with 3750 kg of *Ulva*. A tank system with an area of 1250 m^2^ at a density of 3 kg m^–2^ can provide such an amount of biomass.^88^ Consequently, *Ulva* can be applied in the bioremediation of micropollutants such as xenoestrogens, in addition to nutrient consumption and metal uptake, particularly as part of Integrated Multi-Trophic Aquaculture (IMTA) systems that use *Ulva* alongside fish or shellfish farming. In future studies, the nutrient-dependent hormesis effect of micropollutants and algal biomass production in upscaled applications must be investigated.^89,90^

### Toxicological Assessment of Xenoestrogen Metabolites

Because the transformation products may be more harmful, persistent, or otherwise more hazardous than their parent pollutants, assessing their estrogenic activity, stability, and quantity is of paramount importance. Although the structure–activity relationship of (xeno)estrogens is a highly complex topic and the activity assessment of new compounds requires a multitude of bioassays, several assumptions can be made based on previous studies: degradation products of bisphenols retain receptor affinity proportional to the remaining part of the molecule (as long as they preserve one phenol group).^91^ Bromination generally reduces the receptor affinity of estrogens.^92^ BPA bisulfate is inactive, although this sulfation is reversible.^93,94^ Derivatives or degradation products of ethinylestradiol have also lowered receptor affinity.^95^ Therefore, we expect lower receptor affinity for all identified transformation products than their parent xenoestrogens. However, further bioassay studies with standards of identified transformation products are necessary to obtain reliable receptor affinity and toxicity data.

Compared to BPA, its transformation products displayed low peak areas, which suggest that the transformation products are either intermediates or stable byproducts of negligible abundance (see Figures S5–S7). The transformation products of BPF and EE_2_ were determined by an even lesser degree of abundance. As all transformation products were expected to be less harmful and less abundant than their parent compounds, *Ulva* represents a feasible and sustainable approach for water treatment and the bioremediation of xenoestrogens.

## Conclusions

The removal of xenobiotics is highly species-specific. In the tested reductionist system, *Ulva*, rather than its symbiotic bacteria, was primarily responsible for removing BPA and other xenoestrogens. To disentangle the contributions of the host and its microbiome, investigations of holobionts must be conducted at the level of individual species. *U. mutabilis* achieved xenoestrogen removal primarily through degradation after uptake, producing only minor amounts of stable transformation products. This degradation occurred via a radical mechanism, generating cleavage products and coupling byproducts. Bromoperoxidases and sulfotransferases facilitated further bromination, iodination, or sulfation of transformation products. Importantly, all stable transformation products in *Ulva* were of minimal concern due to their low abundance and reduced endocrine activity.

Identifying the transformation products of BPA, BPF, and EE_2_ offers valuable insights into the biodegradation pathways and highlights the effectiveness of algae, specifically *Ulva*, in breaking down these contaminants. A systematic identification of the enzymes responsible for algal micropollutant metabolism could lead to the discovery of genes that serve as biomarkers for micropollutant removal from wastewater. The findings presented here can guide the development of more effective wastewater treatment strategies that leverage *Ulva* to eliminate harmful xenoestrogens from aquatic environments. Future research will focus on the scalability of employing *Ulva* in diverse environmental contexts and the long-term sustainability of this bioremediation approach.

## Supporting Information Available

Details on methods, additional results and discussion, mass spectra and chromatograms of identified transformation products (PDF)

## References

[ref1] Jiang J. J.; Lee C. L.; Fang M. D. Emerging organic contaminants in coastal waters: anthropogenic impact, environmental release and ecological risk. Mar. Pollut. Bull. 2014, 85 (2), 391–399. 10.1016/j.marpolbul.2013.12.045. 24439316

[ref2] Khan S.; Naushad M.; Govarthanan M.; Iqbal J.; Alfadul S. M. Emerging contaminants of high concern for the environment: Current trends and future research. Environ. Res. 2022, 207, 112609 10.1016/j.envres.2021.112609. 34968428

[ref3] Werkneh A. A.; Gebru S. B.; Redae G. H.; Tsige A. G. Removal of endocrine disrupters from the contaminated environment: public health concerns, treatment strategies and future perspectives - A review. Heliyon 2022, 8 (4), e09206 10.1016/j.heliyon.2022.e09206. 35464705 PMC9026580

[ref4] Watson C. S.; Alyea R. A.; Jeng Y. J.; Kochukov M. Y. Nongenomic actions of low concentration estrogens and xenoestrogens on multiple tissues. Mol. Cell. Endocrinol. 2007, 274 (1–2), 1–7. 10.1016/j.mce.2007.05.011. 17601655 PMC1986712

[ref5] Xiao Y.; Han D.; Currell M.; Song X.; Zhang Y. Review of Endocrine Disrupting Compounds (EDCs) in China’s water environments: Implications for environmental fate, transport and health risks. Water Res. 2023, 245, 120645 10.1016/j.watres.2023.120645. 37769420

[ref6] Vessa B.; Perlman B.; McGovern P. G.; Morelli S. S. Endocrine disruptors and female fertility: a review of pesticide and plasticizer effects. F&S Rep. 2022, 3 (2), 86–90. 10.1016/j.xfre.2022.04.003. PMC9250118 35789730

[ref7] Xing J.; Zhang S.; Zhang M.; Hou J. A critical review of presence, removal and potential impacts of endocrine disruptors bisphenol A. Comp. Biochem. Physiol. C 2022, 254, 109275 10.1016/j.cbpc.2022.109275. 35077873

[ref8] He J.; Xu J.; Zheng M.; Pan K.; Yang L.; Ma L.; Wang C.; Yu J. Thyroid dysfunction caused by exposure to environmental endocrine disruptors and the underlying mechanism: A review. Chem.-Biol. Interact. 2024, 391, 110909 10.1016/j.cbi.2024.110909. 38340975

[ref9] Azizi D.; Arif A.; Blair D.; Dionne J.; Filion Y.; Ouarda Y.; Pazmino A. G.; Pulicharla R.; Rilstone V.; Tiwari B.; Vignale L.; Brar S. K.; Champagne P.; Drogui P.; Langlois V. S.; Blais J. F. A comprehensive review on current technologies for removal of endocrine disrupting chemicals from wastewaters. Environ. Res. 2022, 207, 112196 10.1016/j.envres.2021.112196. 34634314

[ref10] Wiseman A.; Goldfarb P. S.; Ridgway T. J.; Wiseman H. Xenoestrogens and phytoestrogens: bioremediation or biomonitoring?. Trends Biotechnol. 1999, 17 (2), 43 10.1016/S0167-7799(98)01255-4. 10087600

[ref11] Michałowicz J. Bisphenol A – sources, toxicity and biotransformation. Environ. Toxicol. Pharmacol. 2014, 37 (2), 738–758. 10.1016/j.etap.2014.02.003. 24632011

[ref12] Bittner G. D.; Yang C. Z.; Stoner M. A. Estrogenic chemicals often leach from BPA-free plastic products that are replacements for BPA-containing polycarbonate products. Environ. Health 2014, 13 (1), 41 10.1186/1476-069X-13-41. 24886603 PMC4063249

[ref13] Commission Regulation (EU) 2016/2235 of 12 December 2016 amending Annex XVII to Regulation (EC) No 1907/2006 of the European Parliament and of the Council concerning the Registration, Evaluation, Authorisation and Restriction of Chemicals (REACH) as regards bisphenol A (Text with EEA relevance). Off. J. Eur. Union 2016, 337, 3–5.

[ref14] Eladak S.; Grisin T.; Moison D.; Guerquin M. J.; N’Tumba-Byn T.; Pozzi-Gaudin S.; Benachi A.; Livera G.; Rouiller-Fabre V.; Habert R. A new chapter in the bisphenol A story: bisphenol S and bisphenol F are not safe alternatives to this compound. Fertil. Steril. 2015, 103 (1), 11–21. 10.1016/j.fertnstert.2014.11.005. 25475787

[ref15] Tanner E. M.; Hallerback M. U.; Wikstrom S.; Lindh C.; Kiviranta H.; Gennings C.; Bornehag C. G. Early prenatal exposure to suspected endocrine disruptor mixtures is associated with lower IQ at age seven. Environ. Int. 2020, 134, 105185 10.1016/j.envint.2019.105185. 31668669

[ref16] Klaic M.; Jirsa F. 17alpha-Ethinylestradiol (EE_2_): concentrations in the environment and methods for wastewater treatment - an update. RSC Adv. 2022, 12 (20), 12794–12805. 10.1039/D2RA00915C. 35496331 PMC9044539

[ref17] Dietrich M.; Franke M.; Stelter M.; Braeutigam P. Degradation of endocrine disruptor bisphenol A by ultrasound-assisted electrochemical oxidation in water. Ultrason. Sonochem. 2017, 39, 741–749. 10.1016/j.ultsonch.2017.05.038. 28733001

[ref18] Im J.; Loffler F. E. Fate of Bisphenol A in Terrestrial and Aquatic Environments. Environ. Sci. Technol. 2016, 50 (16), 8403–8416. 10.1021/acs.est.6b00877. 27401879

[ref19] Norvill Z. N.; Shilton A.; Guieysse B. Emerging contaminant degradation and removal in algal wastewater treatment ponds: Identifying the research gaps. J. Hazard. Mater. 2016, 313, 291–309. 10.1016/j.jhazmat.2016.03.085. 27135171

[ref20] Mohsenpour S. F.; Hennige S.; Willoughby N.; Adeloye A.; Gutierrez T. Integrating micro-algae into wastewater treatment: A review. Sci. Total Environ. 2021, 752, 142168 10.1016/j.scitotenv.2020.142168. 33207512

[ref21] Viegas C.; Gouveia L.; Goncalves M. Aquaculture wastewater treatment through microalgal. Biomass potential applications on animal feed, agriculture, and energy. J. Environ. Manage. 2021, 286, 112187 10.1016/j.jenvman.2021.112187. 33609932

[ref22] Neori A.; Chopin T.; Troell M.; Buschmann A. H.; Kraemer G. P.; Halling C.; Shpigel M.; Yarish C. Integrated aquaculture: rationale, evolution and state of the art emphasizing seaweed biofiltration in modern mariculture. Aquaculture 2004, 231 (1), 361–391. 10.1016/j.aquaculture.2003.11.015.

[ref23] Neori A. Essential role of seaweed cultivation in integrated multi-trophic aquaculture farms for global expansion of mariculture: an analysis. J. Appl. Phycol. 2008, 20 (5), 567–570. 10.1007/s10811-007-9206-3.

[ref24] Qiu S.; Ge S. J.; Champagne P.; Robertson R. M. Potential of *Ulva lactuca* for municipal wastewater bioremediation and fly food. Desalin. Water Treat. 2017, 91, 23–30. 10.5004/dwt.2017.20767.

[ref25] Liu J. J.; Pemberton B.; Lewis J.; Scales P. J.; Martin G. J. O. Wastewater treatment using filamentous algae - A review. Bioresour. Technol. 2020, 298, 122556 10.1016/j.biortech.2019.122556. 31843358

[ref26] Ichihara K.; Miyaji K.; Shimada S. Comparing the low-salinity tolerance of lva species distributed in different environments. Phycol. Res. 2013, 61 (1), 52–57. 10.1111/j.1440-1835.2012.00668.x.

[ref27] Fricke A.; Harbart V.; Schreiner M.; Baldermann S. A proof of concept for inland production of the “sea-vegetable” *Ulva compressa* in Brandenburg (Central Europe) using regional saline groundwater. Algal Res. 2023, 74, 103226 10.1016/j.algal.2023.103226.

[ref28] Alström-Rapaport C.; Leskinen E.; Pamilo P. Seasonal variation in the mode of reproduction of *Ulva intestinalis* in a brackish water environment. Aquat. Bot. 2010, 93 (4), 244–249. 10.1016/j.aquabot.2010.08.003.

[ref29] Rybak A. S. Freshwater macroalga, *Ulva pilifera* (Ulvaceae, Chlorophyta) as an indicator of the trophic state of waters for small water bodies. Ecol. Indicators 2021, 121, 106951 10.1016/j.ecolind.2020.106951.

[ref30] Zhang C.; Lu J.; Wu J.; Luo Y. Phycoremediation of coastal waters contaminated with bisphenol A by green tidal algae *Ulva prolifera*. Sci. Total Environ. 2019, 661, 55–62. 10.1016/j.scitotenv.2019.01.132. 30665132

[ref31] Hardegen J.; Amend G.; Wichard T. Lifecycle-dependent toxicity and removal of micropollutants in algal cultures of the green seaweed *Ulva* (Chlorophyta). J. Appl. Phycol. 2023, 35, 2031 10.1007/s10811-023-02936-x.

[ref32] Ghaderiardakani F.; Quartino M. L.; Wichard T. Microbiome-Dependent Adaptation of Seaweeds Under Environmental Stresses: A Perspective. Front. Mar. Sci. 2020, 7, 575228 10.3389/fmars.2020.575228.

[ref33] Wichard T. From model organism to application: Bacteria-induced growth and development of the green seaweed *Ulva* and the potential of microbe leveraging in algal aquaculture. Semin. Cell Dev. Biol. 2023, 134, 69 10.1016/j.semcdb.2022.04.007. 35459546

[ref34] Wichard T.; Charrier B.; Mineur F.; Bothwell J. H.; Clerck O. D.; Coates J. C. The green seaweed *Ulva*: a model system to study morphogenesis. Front. Plant Sci. 2015, 6, 72 10.3389/fpls.2015.00072. 25745427 PMC4333771

[ref35] Califano G.; Wichard T. Chapter 9. Preparation of axenic cultures in *Ulva* (Chlorophyta). In Protocols for Macroalgae Research; Charrier B.; Wichard T.; Reddy C. R. K., Eds.; CRC Press: Boca Raton, 2018. 10.1201/b21460.

[ref36] Wichard T. Exploring bacteria-induced growth and morphogenesis in the green macroalga order Ulvales (Chlorophyta). Front. Plant Sci. 2015, 6, 86 10.3389/fpls.2015.00086. 25784916 PMC4347444

[ref37] Zhang C.; Lu J.; Wu J. Enhanced removal of phenolic endocrine disrupting chemicals from coastal waters by intertidal macroalgae. J. Hazard. Mater. 2021, 411, 125105 10.1016/j.jhazmat.2021.125105. 33485233

[ref38] Cheng F.; Wang J. Biological strategies for Bisphenol A degradation: mechanisms and pathways. Rev. Environ. Sci. Biotechnol. 2024, 23, 601 10.1007/s11157-024-09704-4.

[ref39] Azizullah A.; Gao K. S.; Khan S.; Gao G. The interplay between bisphenol A and algae-A review. J. King Saud Univ. Sci. 2022, 34 (5), 102050 10.1016/j.jksus.2022.102050.

[ref40] Steinhagen S.; Barco A.; Wichard T.; Weinberger F. Conspecificity of the model organism *Ulva mutabilis* and *Ulva compressa* (Ulvophyceae, Chlorophyta). J. Phycol. 2019, 55 (1), 25–36. 10.1111/jpy.12804. 30367499

[ref41] Nahor O.; Morales-Reyes C. F.; Califano G.; Wichard T.; Golberg A.; Israel Á. Flow cytometric measurements as a proxy for sporulation intensity in the cultured macroalga *Ulva* (Chlorophyta). Bot. Mar. 2021, 64 (2), 83–92. 10.1515/bot-2020-0050.

[ref42] Hahnke R. L.; Harder J. Phylogenetic diversity of Flavobacteria isolated from the North Sea on solid media. Syst. Appl. Microbiol. 2013, 36 (7), 497–504. 10.1016/j.syapm.2013.06.006. 23957959

[ref43] Ulrich J. F.; Grafe M. S.; Dhiman S.; Wienecke P.; Arndt H. D.; Wichard T. Thallusin Quantification in Marine Bacteria and Algae Cultures. Mar. Drugs 2022, 20 (11), 690 10.3390/md20110690. 36355014 PMC9696546

[ref44] Wiegand I.; Hilpert K.; Hancock R. E. Agar and broth dilution methods to determine the minimal inhibitory concentration (MIC) of antimicrobial substances. Nat. Protoc. 2008, 3 (2), 163–175. 10.1038/nprot.2007.521. 18274517

[ref45] Alsufyani T.; Califano G.; Deicke M.; Grueneberg J.; Weiss A.; Engelen A. H.; Kwantes M.; Mohr J. F.; Ulrich J. F.; Wichard T. Macroalgal bacterial interactions: identification and role of thallusin in morphogenesis of the seaweed *Ulva* (Chlorophyta). J. Exp. Bot. 2020, 71 (11), 3340–3349. 10.1093/jxb/eraa066. 32016363 PMC7289720

[ref46] Kuhlisch C.; Califano G.; Wichard T.; Pohnert G. Metabolomics of intra- and extracellular metabolites from micro- and macroalgae using GC-MS and LC-MS. In Protocols for Macroalgae Research; Charrier B.; Wichard T.; Reddy C. R. K., Eds.; CRC Press: Boca Raton, 2018, Chapter 18.

[ref47] Hardegen J.; Braeutigam P.; Abendroth C.; Wichard T. Bisphenol A: Quantification in Complex Matrices and Removal by Anaerobic Sludges. Pollutants 2021, 1 (4), 194–206. 10.3390/pollutants1040016.

[ref48] Baumeister T. U. H.; Ueberschaar N.; Schmidt-Heck W.; Mohr J. F.; Deicke M.; Wichard T.; Guthke R.; Pohnert G. DeltaMS: a tool to track isotopologues in GC- and LC-MS data. Metabolomics 2018, 14 (4), 41 10.1007/s11306-018-1336-x. 30830340

[ref49] Smith C. A.; Want E. J.; O’Maille G.; Abagyan R.; Siuzdak G. XCMS: processing mass spectrometry data for metabolite profiling using nonlinear peak alignment, matching, and identification. Anal. Chem. 2006, 78 (3), 779–787. 10.1021/ac051437y. 16448051

[ref50] Huang X.; Chen Y. J.; Cho K.; Nikolskiy I.; Crawford P. A.; Patti G. J. X13CMS: global tracking of isotopic labels in untargeted metabolomics. Anal. Chem. 2014, 86 (3), 1632–1639. 10.1021/ac403384n. 24397582 PMC3982964

[ref51] McLafferty F. W.; Tureček Fe. Interpretation of Mass Spectra, 4th ed.; University Science Books: Mill Valley, Calif, 1993.

[ref52] Doumas M.; Rouillon S.; Venisse N.; Nadeau C.; Pierre Eugene P.; Farce A.; Chavatte P.; Dupuis A.; Migeot V.; Carato P. Chlorinated and brominated bisphenol A derivatives: Synthesis, characterization and determination in water samples. Chemosphere 2018, 213, 434–442. 10.1016/j.chemosphere.2018.09.061. 30243209

[ref53] Vasiljevic T.; Harner T. Bisphenol A and its analogues in outdoor and indoor air: Properties, sources and global levels. Sci. Total Environ. 2021, 789, 148013 10.1016/j.scitotenv.2021.148013. 34323825

[ref54] Yang N. J.; Hinner M. J. Getting across the cell membrane: an overview for small molecules, peptides, and proteins. Methods Mol. Biol. 2015, 1266, 29–53. 10.1007/978-1-4939-2272-7_3. 25560066 PMC4891184

[ref55] Flodin C.; Whitfield F. Biosynthesis of bromophenols in marine algae. Water Sci. Technol. 1999, 40 (6), 53–58. 10.2166/wst.1999.0260.

[ref56] Flodin C.; Helidoniotis F.; Whitfield F. B. Seasonal variation in bromophenol content andbromoperoxidase activity in *Ulva lactuca*. Phytochemistry 1999, 51 (1), 135–138. 10.1016/S0031-9422(98)00668-2.

[ref57] Neilson A. H. Biological Effects and Biosynthesis of Brominated Metabolites. In Handbook of Environmental Chemistry, 2003; Vol. 3R, pp 75–204.

[ref58] Astrahan P.; Korzen L.; Khanin M.; Sharoni Y.; Israel A. Seaweeds fast EDC bioremediation: Supporting evidence of EE_2_ and BPA degradation by the red seaweed Gracilaria sp., and a proposed model for the remedy of marine-borne phenol pollutants. Environ. Pollut. 2021, 278, 116853 10.1016/j.envpol.2021.116853. 33740605

[ref59] Mandrekar V. K.; Gawas U. B.; Majik M. S. Brominated Molecules From Marine Algae and Their Pharmacological Importance. In Studies in Natural Products Chemistry; Atta-ur-Rahman, Ed.; Elsevier, 2019; Vol. 61. Chapter 13.

[ref60] Senn H. M. Insights into enzymatic halogenation from computational studies. Front. Chem. 2014, 2, 98 10.3389/fchem.2014.00098. 25426489 PMC4227530

[ref61] Latham J.; Brandenburger E.; Shepherd S. A.; Menon B. R. K.; Micklefield J. Development of Halogenase Enzymes for Use in Synthesis. Chem. Rev. 2018, 118 (1), 232–269. 10.1021/acs.chemrev.7b00032. 28466644

[ref62] Höfler G. T.; But A.; Hollmann F. Haloperoxidases as catalysts in organic synthesis. Org. Biomol. Chem. 2019, 17 (42), 9267–9274. 10.1039/C9OB01884K. 31599911

[ref63] Ludewig H.; Molyneux S.; Ferrinho S.; Guo K.; Lynch R.; Gkotsi D. S.; Goss R. J. M. Halogenases: structures and functions. Curr. Opin. Struct. Biol. 2020, 65, 51–60. 10.1016/j.sbi.2020.05.012. 32619660

[ref64] Gérard E. F.; Mokkawes T.; Johannissen L. O.; Warwicker J.; Spiess R. R.; Blanford C. F.; Hay S.; Heyes D. J.; de Visser S. P. How Is Substrate Halogenation Triggered by the Vanadium Haloperoxidase from *Curvularia inaequalis*?. ACS Catal. 2023, 13 (12), 8247–8261. 10.1021/acscatal.3c00761. 37342830 PMC10278073

[ref65] Gkotsi D. S.; Dhaliwal J.; McLachlan M. M. W.; Mulholand K. R.; Goss R. J. M. Halogenases: powerful tools for biocatalysis (mechanisms applications and scope). Curr. Opin. Chem. Biol. 2018, 43, 119–126. 10.1016/j.cbpa.2018.01.002. 29414530

[ref66] De Clerck O.; Kao S. M.; Bogaert K. A.; Blomme J.; Foflonker F.; Kwantes M.; Vancaester E.; Vanderstraeten L.; Aydogdu E.; Boesger J.; Califano G.; Charrier B.; Clewes R.; Del Cortona A.; D’Hondt S.; Fernandez-Pozo N.; Gachon C. M.; Hanikenne M.; Lattermann L.; Leliaert F.; Liu X. J.; Maggs C. A.; Popper Z. A.; Raven J. A.; Van Bel M.; Wilhelmsson P. K. I.; Bhattacharya D.; Coates J. C.; Rensing S. A.; Van Der Straeten D.; Vardi A.; Sterck L.; Vandepoele K.; Van de Peer Y.; Wichard T.; Bothwell J. H. Insights into the Evolution of Multicellularity from the Sea Lettuce Genome. Curr. Biol. 2018, 28 (18), 2921 10.1016/j.cub.2018.08.015. 30220504

[ref67] Huang Q.; Weber W. J. Jr. Transformation and removal of bisphenol A from aqueous phase via peroxidase mediated oxidative coupling reactions: efficacy, products, and pathways. Environ. Sci. Technol. 2005, 39 (16), 6029–6036. 10.1021/es050036x. 16173560

[ref68] Kyrila G.; Katsoulas A.; Schoretsaniti V.; Rigopoulos A.; Rizou E.; Doulgeridou S.; Sarli V.; Samanidou V.; Touraki M. Bisphenol A removal and degradation pathways in microorganisms with probiotic properties. J. Hazard. Mater. 2021, 413, 125363 10.1016/j.jhazmat.2021.125363. 33592490

[ref69] Wang R.; Diao P.; Chen Q.; Wu H.; Xu N.; Duan S. Identification of novel pathways for biodegradation of bisphenol A by the green alga *Desmodesmus* sp.WR1, combined with mechanistic analysis at the transcriptome level. Chem. Eng. J. 2017, 321, 424–431. 10.1016/j.cej.2017.03.121.

[ref70] Xu Z.; Jia Y.; Zhang X.; Hu S.; Luo Y.; He H.; Chen B.; Huang B.; Pan X. Algal organic matter accelerates the photodegradation of tetracycline: Mechanisms, degradation pathways and product toxicity. Chem. Eng. J. 2023, 468, 143724 10.1016/j.cej.2023.143724.

[ref71] Maes H. M.; Maletz S. X.; Ratte H. T.; Hollender J.; Schaeffer A. Uptake, Elimination, and Biotransformation of 17α-Ethinylestradiol by the Freshwater Alga *Desmodesmus subspicatus*. Environ. Sci. Technol. 2014, 48 (20), 12354–12361. 10.1021/es503574z. 25238549

[ref72] Noszczyńska M.; Chodór M.; Jałowiecki Ł.; Piotrowska-Seget Z. A comprehensive study on bisphenol A degradation by newly isolated strains *Acinetobacter* sp. K1MN and *Pseudomonas* sp. BG12. Biodegradation 2021, 32 (1), 1–15. 10.1007/s10532-020-09919-6. 33205349 PMC7940318

[ref73] Suyamud B.; Inthorn D.; Panyapinyopol B.; Thiravetyan P. Biodegradation of Bisphenol A by a Newly Isolated *Bacillus megaterium* Strain ISO-2 from a Polycarbonate Industrial Wastewater. Water, Air, Soil Pollut. 2018, 229 (11), 348 10.1007/s11270-018-3983-y.

[ref74] Li G.; Zu L.; Wong P. K.; Hui X.; Lu Y.; Xiong J.; An T. Biodegradation and detoxification of bisphenol A with one newly-isolated strain *Bacillus* sp. GZB: kinetics, mechanism and estrogenic transition. Bioresour. Technol. 2012, 114, 224–230. 10.1016/j.biortech.2012.03.067. 22507902

[ref75] Zühlke M.-K.; Schlüter R.; Mikolasch A.; Zühlke D.; Giersberg M.; Schindler H.; Henning A.-K.; Frenzel H.; Hammer E.; Lalk M.; Bornscheuer U. T.; Riedel K.; Kunze G.; Schauer F. Biotransformation and reduction of estrogenicity of bisphenol A by the biphenyl-degrading *Cupriavidus basilensis*. Appl. Microbiol. Biotechnol. 2017, 101 (9), 3743–3758. 10.1007/s00253-016-8061-z. 28050635

[ref76] Heidari H.; Sedighi M.; Zamir S. M.; Shojaosadati S. A. Bisphenol A degradation by *Ralstonia eutropha* in the absence and presence of phenol. Int. Biodeterior. Biodegrad. 2017, 119, 37–42. 10.1016/j.ibiod.2016.10.052.

[ref77] Peng Y.-H.; Chen Y.-J.; Chang Y.-J.; Shih Y.-h. Biodegradation of bisphenol A with diverse microorganisms from river sediment. J. Hazard. Mater. 2015, 286, 285–290. 10.1016/j.jhazmat.2014.12.051. 25590822

[ref78] Louati I.; Dammak M.; Nasri R.; Belbahri L.; Nasri M.; Abdelkafi S.; Mechichi T. Biodegradation and detoxification of bisphenol A by bacteria isolated from desert soils. 3 Biotech 2019, 9 (6), 228 10.1007/s13205-019-1756-y. PMC6531583 31139543

[ref79] Eltoukhy A.; Jia Y.; Nahurira R.; Abo-Kadoum M. A.; Khokhar I.; Wang J.; Yan Y. Biodegradation of endocrine disruptor Bisphenol A by *Pseudomonas putida* strain YC-AE1 isolated from polluted soil, Guangdong, China. BMC Microbiol. 2020, 20 (1), 11 10.1186/s12866-020-1699-9. 31931706 PMC6958771

[ref80] Jia Y.; Eltoukhy A.; Wang J.; Li X.; Hlaing T. S.; Aung M. M.; Nwe M. T.; Lamraoui I.; Yan Y. Biodegradation of Bisphenol A by *Sphingobium* sp. YC-JY1 and the Essential Role of Cytochrome P450 Monooxygenase. Int. J. Molecular Sci. 2020, 21, 3588 10.3390/ijms21103588. PMC7278973 32438730

[ref81] Hirooka T.; Nagase H.; Uchida K.; Hiroshige Y.; Ehara Y.; Nishikawa Ji.; Nishihara T.; Miyamoto K.; Hirata Z. Biodegradation of bisphenol a and disappearance of its estrogenic activity by the green alga *Chlorella fusca* var. *vacuolata*. Environ. Toxicol. Chem. 2005, 24 (8), 1896–1901. 10.1897/04-259R.1. 16152959

[ref82] Ben Ouada S.; Ben Ali R.; Leboulanger C.; Zaghden H.; Choura S.; Ben Ouada H.; Sayadi S. Effect and removal of bisphenol A by two extremophilic microalgal strains (Chlorophyta). J. Appl. Phycol. 2018, 30 (3), 1765–1776. 10.1007/s10811-017-1386-x.

[ref83] Gattullo C. E.; Bährs H.; Steinberg C. E. W.; Loffredo E. Removal of bisphenol A by the freshwater green alga *Monoraphidium braunii* and the role of natural organic matter. Sci. Total Environ. 2012, 416, 501–506. 10.1016/j.scitotenv.2011.11.033. 22209372

[ref84] Ben Ouada S.; Ben Ali R.; Leboulanger C.; Ben Ouada H.; Sayadi S. Effect of Bisphenol A on the extremophilic microalgal strain *Picocystis* sp. (Chlorophyta) and its high BPA removal ability. Ecotoxicol. Environ. Saf. 2018, 158, 1–8. 10.1016/j.ecoenv.2018.04.008. 29656159

[ref85] Atengueño-Reyes K.; Velásquez-Orta S. B.; Yáñez-Noguez I.; Monje-Ramírez I.; Orta-Ledesma M. T. Removal processes and estrogenic activity of bisphenol—A and triclosan using microalgae. Algal Res. 2024, 82, 103670 10.1016/j.algal.2024.103670.

[ref86] Zhang G.; Wang Y.; Jiang J.; Yang S. Bisphenol A Removal by Submerged Macrophytes and the Contribution of Epiphytic Microorganisms to the Removal Process. Bull. Environ. Contam. Toxicol. 2017, 98 (6), 770–775. 10.1007/s00128-017-2071-0. 28361461

[ref87] Hardegen J.; Wichard T.; Braeutigam P.; Michael S. Das Abbauverhalten von Bisphenol A. KA Betriebsinfo 2024, 2, 3478–3482.

[ref88] Msuya F. E. The Effect of Stocking Density on the Performance of the Seaweed *Ulva reticulata* as a Biofilter in Earthen Pond Channels, Zanzibar, Tanzania. Western Indian Ocean J. Mar. Sci. 2007, 6 (1), 65–72. 10.4314/wiojms.v6i1.48227.

[ref89] Liu Y.; Wang F.; Chen X.; Zhang J.; Gao B. Cellular responses and biodegradation of amoxicillin in *Microcystis aeruginosa* at different nitrogen levels. Ecotoxicol. Environ. Saf. 2015, 111, 138–145. 10.1016/j.ecoenv.2014.10.011. 25450926

[ref90] Zhang Y.; Gao Q.; Liu S.-s.; Tang L.; Li X.-G.; Sun H. Hormetic dose-response of halogenated organic pollutants on *Microcystis aeruginosa*: Joint toxic action and mechanism. Sci. Total Environ. 2022, 829, 154581 10.1016/j.scitotenv.2022.154581. 35304143

[ref91] Okada H.; Tokunaga T.; Liu X.; Takayanagi S.; Matsushima A.; Shimohigashi Y. Direct evidence revealing structural elements essential for the high binding ability of bisphenol A to human estrogen-related receptor-gamma. Environ. Health Perspect. 2008, 116 (1), 32–38. 10.1289/ehp.10587. 18197296 PMC2199305

[ref92] Vollmer G.; Wunsche W.; Schutze N.; Feit B.; Knuppen R. Methyl and bromo derivatives of estradiol are agonistic ligands for the estrogen receptor of MCF-7 breast cancer cells. J. Steroid Biochem. Mol. Biol. 1991, 39 (3), 359–366. 10.1016/0960-0760(91)90047-9. 1911426

[ref93] Durcik M.; Gramec Skledar D.; Tomasic T.; Trontelj J.; Peterlin Masic L. Last piece in the puzzle of bisphenols BPA, BPS and BPF metabolism: Kinetics of the in vitro sulfation reaction. Chemosphere 2022, 303 (Pt 2), 135133 10.1016/j.chemosphere.2022.135133. 35636595

[ref94] Allard P. Bisphenol A. In Biomarkers in Toxicology; Gupta R. C., Ed.; Academic Press, 2014; Chapter 27, pp 459–474.

[ref95] Jordan V. C.; Mittal S.; Gosden B.; Koch R.; Lieberman M. E. Structure-activity relationships of estrogens. Environ. Health Perspect. 1985, 61, 97–110. 10.1289/ehp.856197. 3905383 PMC1568776

