# Supplementary material for: Biodegradation of Xenoestrogens by the Green Tide Forming Seaweed Ulva: A Model System for Bioremediation
Source: ACS ES T Water. 2025 Mar 5;5(3):1195–206. doi: 10.1021/acsestwater.4c00961 (PMC11915382; doi:10.1021/acsestwater.4c00961)
Supplement: Supplementary file 1 — ew4c00961_si_001.pdf [file ew4c00961_si_001.pdf]

Supporting Information for

Biodegradation of Xenoestrogens by the Green Tide Forming Seaweed *Ulva*: A Model System for Bioremediation

Justus B. Hardegen, Maximilian S. F. Knips, Johanna K. Däumer, Svenja Kretzer,

and Thomas Wichard[*]

Friedrich Schiller University Jena,

Institute for Inorganic and Analytical Chemistry, Jena, Germany

[*]Corresponding author: thomas.wichard@uni-jena.de

Content

Content 2
1. Standard methods and additional experiments (**Figures S1-S2, Tables S1-S6**) 4
1.1. Chemicals 4
1.2. Effective concentration of bisphenols for *Ulva mutabilis* 4
1.3. Kinetic of BPA with different *Ulva* morphotypes and conditions 4
1.4. Quantification of xenoestrogens in aqueous samples 5
1.5. Quantification of xenoestrogens in algal tissue 7
1.6. Identification of transformation products by stable isotope labeling 8
1.7. Time course of transformation products of BPA 10
2. Additional results and discussion (**Figures S3-S7, Tables S7-S8)** 11
2.1. Toxicity of bisphenols towards *Ulva* 11
2.2. Removal kinetics of BPA with different morphotypes and conditions 13
2.3. Transformation products of BPA 14
2.4. Time course of transformation products 16
3. Transformation products of bisphenol A (**Figures S8-S33, Table S9**) 20
3.1. Example of structural elucidation 21
3.2. Propenylphenol (PP) 23
3.3. Methoxypropanylphenol (MPP) 24
3.4. Ethoxypropanylphenol (EPP) 25
3.5. Hydroxypropanylphenol (HPP) 26
3.6. Hydroxyacetophenone (HAP) 28
3.7. Bromohydroxypropanylphenol (BrHPP) 29
3.8. Dibromohydroxypropanylphenol (Br_2HPP) 30
3.9. Bromobisphenol A (BrBPA) 31
3.10. Iodobisphenol A (IBPA) 33
3.11. 3,5-Dibromobisphenol A (3,5-Br_2BPA) 34
3.12. 3,3'-Dibromobisphenol A (3,3'-Br_2BPA) 35
3.13. Comparison of isomers of dibromobisphenol A (Br_2BPA) 36
3.14. Assignment of isomers of dibromobisphenol A (Br_2BPA) 37
3.15. Isomers of bromoiodobisphenol A (BrIBPA) 38
3.16. Polybrominated bisphenol A 39
3.17. Tribromobisphenol A (Br_3BPA) 40
3.18. Tetrabromobisphenol A (Br_4BPA) 41
3.19. Bisphenol A-hydroquinone ether (BPA-HQ) 42
3.20. Brominated bisphenol A-hydroquinone ether isomers 43
3.21. Bisphenol A bisulfate 44
3.22. Bromobisphenol A bisulfate 45

3.23. Dibromobisphenol A bisulfate 46
3.24. Additionally detected features with $^{13}C_{6n}$ label 47
4. Transformation products of bisphenol F (**Figures S34-S37**) 48
4.1. Ethoxymethylphenol 48
4.2. Hydroxybenzaldehyde 49
Hydroxymethylphenol 50
4.3. Aminomethylphenol 51
5. Transformation products of ethinylestradiol (**Figures S38-S39**) 52
5.1. Isomers of bromoethinylestradiol 52
6. References 54

1. Standard methods and additional experiments

1.1. Chemicals

Analytical standards of the bisphenols BPB, BPE, BPF, BPP, BPS, and BPZ were purchased from LGC Standards (England), while bisphenol A (BPA) and 17α-ethinylestradiol (EE_2) were purchased from Alfa Aesar (USA) and Sigma Aldrich/Merck (Germany), respectively. The isotopologues bisphenol B-D_8, E-D_{12}, F-D_{10} (CAS 1794786-93-8), P-D_{16}, S-D_8, and Z-D_6 were purchased from LGC standards (England), bisphenol A-D_{16} (CAS 96210-87-6) from CDN Isotopes (Canada), bisphenol-A-(diphenyl-$^{13}C_{12}$) (CAS 263261-65-0) from Sigma Aldrich/Merck (Germany), and 17α-ethinylestradiol-D_4 (2,4,16,16-D_4, CAS 350820-06-3) from Cambridge Isotope Laboratories (Canada).

1.2. Effective concentration of bisphenols for *Ulva mutabilis*

The effect of all bisphenols on *Ulva mutabilis* Føyn (sl-G[mt+]; morphotype 'slender'; locus typicus: Ria Formosa, Portugal); strain FSU-UM5-1) in its tripartite community was determined, as reported in our previous publication.[1] In short, 3-week-old *Ulva* individuals (0.5–1.0 cm long) were incubated at different concentrations of bisphenols in UCM for 14 days. The normalized average reflectance in the fluorescence spectrum (nARFS) upon excitation of actinic light was calculated and plotted against concentrations. Curves were fitted with a dose-response model.[2]

1.3. Kinetics of BPA with different *Ulva* morphotypes and conditions

Haploid gametophytes of *U. mutabilis* (morphotype 'wildytype'; strain FSU-UM1-41) propagated with its natural microbiome since 1952 were used additionally in this experiment.[3]

Several *U. mutabilis* cultures were incubated with 6.6 mg L^{-1} BPA in UCM in biological triplicates: slender morphotype in a tripartite community with light (1), without light (2), or dead after autoclaving at 121 °C for 20 min (3); slender morphotype grown and tested axenic as deformed callus shape (4); wildtype morphotype with natural microbiome (5). Furthermore, three abiotic controls were prepared (6). 0.1 mg BPA per 1 g *Ulva* (fresh weight) was added to the culture.

1.4. Quantification of xenoestrogens in aqueous samples

Aqueous samples were spiked with stable isotope-labeled internal standards (50 % of the initial molar amount of xenoestrogens). Samples were diluted to the working range of the calibration and filtered with 0.22-µm PVDF filters. 2 µL were injected and separated in a guarded ACQUITY UPLC BEH C_{18} column (130 Å, 1.7 µm, 100 × 2.1 mm, Waters, Ireland) over 10 minutes with a 7-minute gradient from eluent A to B with 0.3 mL min^{-1} flow rate (**Table S1**):

- Eluent A (aqueous): 90 % water, 10 % methanol, (v:v), 2 mmol L^{-1} $(NH_4)_2CO_3$
- Eluent B (organic): 100 % methanol, (v:v), 2 mmol L^{-1} $(NH_4)_2CO_3$

The analytes and internal standards were detected as deprotonated ions (**Table S2**) with the Q Exactive™ hybrid quadrupole-Orbitrap mass spectrometer (Thermo Fisher Scientific, USA). Base calibrations were used for quantification (**Fig. S1**, **Table S3**).

Table S1. Gradient elution of xenoestrogens in ultra-high-pressure liquid chromatography

Time (min)	Eluent A (%)	Eluent B (%)
**0**	100	0
**0.5**	100	0
**7.5**	0	100
**9**	0	100
**9.1**	100	0
**10**	100	0

Table S2. Quantifying ions and retention time for xenoestrogens and internal standards in UHPLC-ESI-HRMS. *Bisphenol A was entirely labeled, but the two phenolic deuterium atoms were exchanged for hydrogen atoms during chromatographic separation.

Compound	Retention time (min)	Quantifying ion	Polarity	Mass of analyte (*m*/*z*)	Mass of internal standard (*m*/*z*)	Label of internal standard
**Bisphenol A**	7.31	$(M-H)^-$	Negative	227.10775	241.19563	D_{14}*
**Bisphenol B**	7.66	$(M-H)^-$	Negative	241.12340	249.17362	D_8
**Bisphenol E**	7.04	$(M-H)^-$	Negative	213.09210	225.16742	D_{12}
**Bisphenol F**	6.71	$(M-H)^-$	Negative	199.07645	209.13922	D_{10}
**Bisphenol P**	8.68	$(M-H)^-$	Negative	345.18600	361.28643	D_{16}
**Bisphenol S**	1.68	$(M-H)^-$	Negative	249.02270	257.07292	D_8
**Bisphenol Z**	8.09	$(M-H)^-$	Negative	267.13905	273.17671	D_6
**Ethinylestradiol**	7.67	$(M-H)^-$	Negative	295.17035	299.19546	D_4

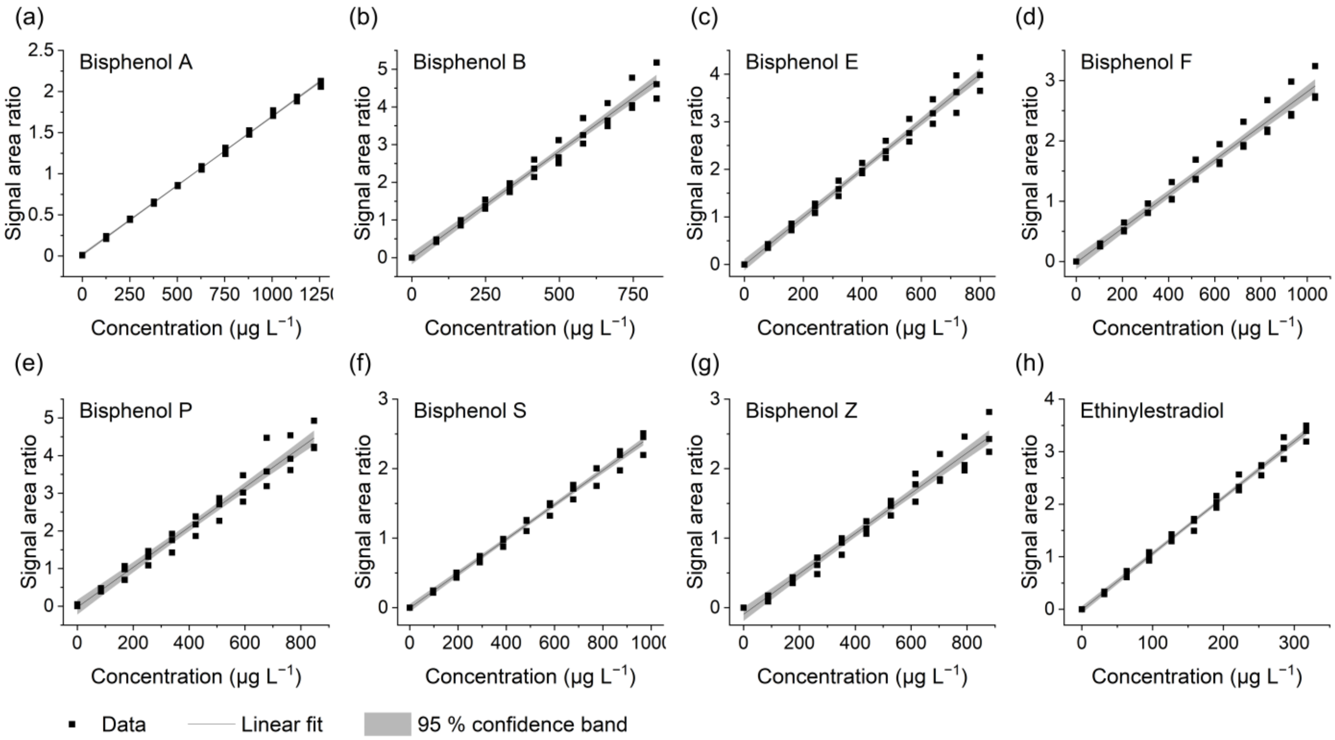

Figure S1. Base calibration for xenoestrogens concentration in aqueous samples. Calibration of bisphenol A (a), Bisphenol B (b), Bisphenol E (c), Bisphenol F (d), Bisphenol P (e), Bisphenol S (f), Bisphenol Z (g), and ethinylestradiol (h) with concentration versus signal area ratio. The signal area ratio was calculated using an isotopically labelled standard that was added at half the maximum concentration of the calibration.

Table S3. Calibration parameters for the concentration of xenoestrogens in aqueous samples. Intercept and slope are displayed with 95 % confidence intervals. (The calibration curve could be further simplified as the intercept did not differ significantly from zero.)

Compound	Intercept	Slope (L μg^{-1})	RSD (%)
**Bisphenol A**	0.01698 ± 0.01675	1680 ± 230	2.4
**Bisphenol B**	-0.01948 ± 0.15596	5690 ± 318	10.0
**Bisphenol E**	-0.00665 ± 0.11875	5015 ± 251	8.9
**Bisphenol F**	-0.01668 ± 0.11251	2829 ± 184	11.6
**Bisphenol P**	-0.02701 ± 0.19485	5309 ± 389	13.0
**Bisphenol S**	-0.00373 ± 0.05548	2474 ± 970	7.0
**Bisphenol Z**	-0.09411 ± 0.09672	2902 ± 186	11.4
**Ethinylestradiol**	-0.00764 ± 0.06665	10678 ± 355	5.9

1.5. Quantification of xenoestrogens in algal tissue

Alga tissue samples were washed three times by submersion in ultra-pure water, freeze-dried, and pulverized. The weight of the samples was determined before and after drying. Xenoestrogens were extracted with a mixture of ethanol, methanol, chloroform (60:20:20), and isotopologues internal standards (equaling 1 % of the initial amount) for 10 minutes in an ultrasonic bath. The mixture was centrifuged, the supernatant was transferred, and the solvents were carefully evaporated under a vacuum. The residue was resuspended in 200 µL pyridine, 50 µL of the solution derivatized with 50 µL MSTFA at 60 °C for 60 min and measured via GC-EI-HRMS. Measurements were performed with a Zebron ZB-SemiVolatiles column (30 m × 0.25 mm × 0.25 µm, Phenomenex, Aschaffenburg, Germany) in a Trace™ 1310 GC coupled to a Q-Exactive™ Orbitrap GC™. The injection volume was 2 µL, split 50, and liner temperature 250 °C. The analytes were separated over 36 min with a helium flow rate of 1.2 mL min^{-1} and a temperature ramp from 80 to 320 °C over 30 min with 2 min hold time at 80 °C and 4 min at 320 °C. Mass spectrometry was performed with an electron impact source at 300 °C, 70 eV electron energy, 9 min filament delay, and full MS mode with a scan range between 300 and 450 *m*/*z* and a resolution of 120,000. Most abundant ions (molecular ions or fragments) were used for quantification (Table S4). Base calibrations were used for quantification (**Fig. S2**, **table S5**).

Table S4. Quantifying ions for the determination of xenoestrogens. The retention time of analytes and internal standards are given for GC-EI-HRMS after derivatization with MSTFA. *Bisphenol A was entirely labeled, but the two phenolic deuterium atoms were lost during derivatization, and three more during fragmentation.

		**Analyte**		**Internal standard**		
**Compound**	Quantifying ion	Retention time (min)	Mass (*m*/*z*)	Retention time (min)	Mass (*m*/*z*)	Label
**Bisphenol A**	$(M{-}CH_3)^+$	22.16	357.17006	22.06	368.23910	D_{11}*
**Bisphenol F**	$(M)^{+\bullet}$	21.47	344.16223	21.41	354.22500	D_{10}
**Ethinylestradiol**	$(M{-}CH_3)^+$	27.94	425.23266	27.92	429.25777	D_4

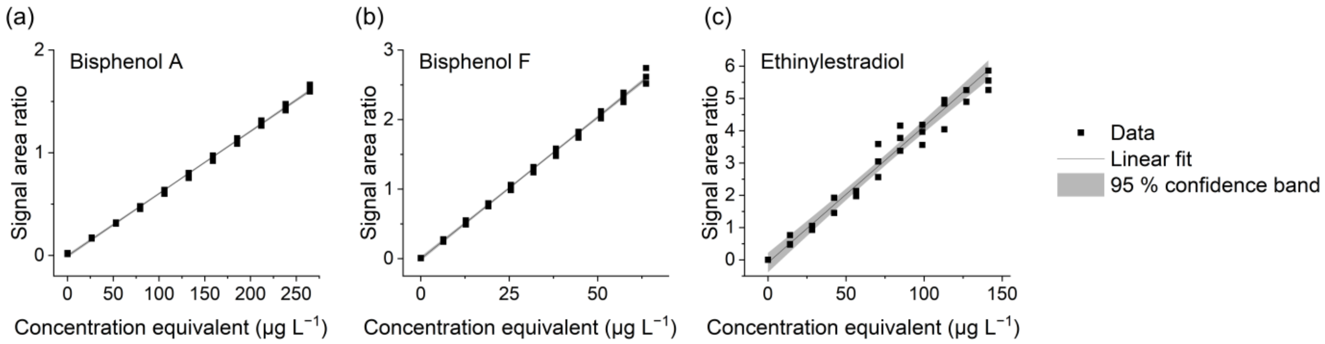

Figure S2. Base calibration for xenoestrogens in algal tissue. Calibration of bisphenol A (a), Bisphenol F (b), and ethinylestradiol (c) with concentration equivalent versus signal area ratio. The concentration equivalent refers to the amount of xenoestrogen absorbed divided by the initial volume. The signal area ratio was calculated using an isotopically labelled standard added at half the maximum concentration of the calibration.

Table S5. Calibration parameters for xenoestrogens in algal tissue. Intercept and slope are displayed with 95 % confidence intervals. (The calibration curve could be further simplified as the intercept did not differ significantly from zero.)

Compound	Intercept	Slope (L µg^{-1})	RSD (%)
**Bisphenol A**	-0.00454 ± 0.01611	0.00607 ± 0.0001	3
**Bisphenol F**	-0.00175 ± 0.03071	0.04073 ± 0.00082	3.6
**Ethinylestradiol**	-0.08500 ± 0.30152	0.04224 ± 0.00361	15.2

1.6. Identification of transformation products by stable isotope labeling

About 100 mg of tripartite *U. mutabilis* was incubated with unlabeled and labeled analogues of BPA, BPF, and EE2 in 2 mL UCM with five biological replicates each: 58.0 µmol L^{-1} unlabeled BPA, fully deuterated BPA, or diphenyl-$^{13}C_{12}$ labeled BPA; 15.9 µmol L^{-1} unlabeled BPF, or D_{10} labeled BPF; 9.5 µmol L^{-1} unlabeled EE_2, or D_4 labeled EE_2. After two days, alga tissue samples were washed by submersion three times in ultra-pure water, immediately frozen in liquid nitrogen, freeze-dried and pulverized. Intracellular metabolites were extracted with a mixture of ethanol, methanol, chloroform (60:20:20), and 20 µmol L^{-1} of $^{13}C_1$ labeled sorbitol for 10 minutes in an ultrasonic bath.[4] The volume of the mixture was added in proportion to the dry mass of the tissue with a maximum of 1.5 mL for ca. 4.5 mg. The mixture was centrifuged, and 300 µL of the supernatant was transferred for GC and 300 µL for LC analysis. Additionally, appropriate quality controls were prepared. The solvents were evaporated under vacuum, followed by GC and LC metabolomic profiling and data analysis.

1.6.1. GC metabolomic profiling

Dried extracts were dissolved in 50 µL methoxyamine pyridine solution (20 g L^{-1}), derivatized at 60 °C for 1 h, and incubated at room temperature for 16 h. 40 µL of this solution were further derivatized with 40 µL MSTFA 60 °C for 1 h. 1 µL of the final product was injected into the aforementioned GC-EI-HRMS with a split of 10 and a liner temperature of 250 °C. The analytes were separated over 40 min with a helium flow rate of 1 mL min−1 and a temperature ramp with 2 min hold time at 80 °C, increase to 120 °C over 2 min, 1 min hold time, increase to 250 °C over 26 min, increase to 320 °C over 7 min, and 2 min hold time. Mass spectrometry was performed with an electron impact source at 300 °C, 70 eV electron energy, 5.7 min filament delay, full MS mode with a scan range between 50 and 600 *m*/*z* and a resolution of 120,000. For additional targeted searches of postulated transformation products, the scan range was changed to between 53 and 800 *m/z*.

1.6.2. LC metabolomic profiling

Metabolome measurements were performed with the Q Exactive™ hybrid quadrupole-Orbitrap mass spectrometer (Thermo Fisher Scientific, USA) with ESI. Dried extracts were dissolved in methanol, and 2 µL injected. The analytes were separated in a guarded Accucore UPLC C18 column (80 Å, 2.6 µm, 100 × 2.1 mm, Thermo Fisher Scientific, USA) over 12 minutes with a 7.8-minute gradient from 0 % to 100 % organic (acetonitrile) to aqueous (water, 2 % acetonitrile, and 0.1 % formic acid). Analytes were detected in positive and negative mode in individual injections with 280,000 resolution and a scan range of 80 to 1200 *m*/*z*. After data analysis, identified transformation products were further characterized via parallel reaction monitoring (MS^2) using the collision energies 15, 35, and 55 eV, resolution 280,000, and isolation window of 0.4 *m*/*z*.

1.6.3. Data analysis

Thermo .raw files were converted to .mzXML using the MSconvertGUI software tool with default settings and vendor peak picking.[5] Data was processes with XCMS and X^{13}CMS.[6, 7] For this, the R-script was taken from Meyer *et al.* (2022).[8] Carbon isotopologue pairs (^{12}C and ^{13}C) were identified with the following parameters: centWave, ppm = 3, peakwidth = c(5,20)) isotopeMassDiff = 1.00335, RTwindow = 10, ppm = 3, massOfLabeledAtom = 12.00000, noiseCutoff = 10000, intChoice = "intb", varEq = FALSE, alpha = 0.05, singleSample = FALSE, compareOnlyDistros = FALSE, monotonicityTol = FALSE, enrichTol = 0.1. Hydrogen

isotopologue pairs (^{1}H and ^{2}H) were identified with isotopeMassDiff = 1.00628 and massOfLabeledAtom = 1.00783, and the other parameters being identical. Data annotations were confirmed by manual inspection.

1.7. Time course of transformation products of BPA

Backup samples of the 16-day experiment for kinetics and bioaccumulation of BPA were reevaluated to receive temporal information on transformation products. For GC analytes, 50 µL of extracts in pyridine were derivatized with 25 µL MSTFA and 1 µL measured with the GC metabolomic profiling method. Additionally, the LC-MS data of the kinetics experiment with different *Ulva* morphotypes and conditions (**Chapter 1.3**) was reevaluated. For a semi-quantitative approach, the peak areas of the most abundant specific ions of each identified transformation product were calculated (Tables S7 and S8).

Due to the experimental setup of the original bioaccumulation experiment, the LC/MS analytes were detected in the medium (*exo*-metabolome) and the GC/MS analytes in the tissue (*endo*-metabolome). Several GC analytes were not included in the time course: the products were detected in trace amounts, such as hydroxyacetophenone, which was not derivatized due to the experimental setup.

Peak areas were normalized with internal standard (BPA-D_{16}) area. To preserve the magnitude of the original areas, each peak area was divided by the area of the internal standard of its respective sample and multiplied by the mean area of the IS of all samples (of the experiment). LC samples were also corrected for concentration increase due to the evaporation of water. Peak areas were plotted versus time and fitted with a first-order consecutive reversible reaction kinetics model. The model considers the concentration (or peak area in this case) of any transformation product (B) in equilibrium with its precursor (A) and secondary transformation product (C) $A \rightleftharpoons B \rightleftharpoons C$ with four respective first-order reaction rate constants. The starting concentrations were 100 % for A and 0 % for both B and C. Fits were performed using an iterative generalized reduced gradient algorithm minimizing the sum of squares, including weighting to counteract the temporal non-linear data. BPA was also fitted with this model since BPA uptake was considered reversible first-order kinetic: BPA remaining in the medium was fitted as A and accumulated BPA as B.

2. Additional results and discussion

2.1. Toxicity of bisphenols towards *Ulva*

The toxic effect of all bisphenols was determined for *Ulva* in the tripartite community. The chlorophyll-*a* (chl-*a*) fluorescence was evaluated after 14 days of incubation with varying concentrations (**Fig. S3**) according to established protocols.[9] The bisphenols A, B, E, F, and Z showed a clear dose-response relationship, while BPP and BPS were not toxic for different reasons: BPP had a low solubility and was not toxic at saturated concentration. Nevertheless, the (chl-*a*) fluorescence decreased with increasing nominal concentration (added amount per volume, party precipitated), and the dose-response model was successfully fitted. While BPS was considerably more soluble, no significant decrease of chl-*a* fluorescence was observed over the tested range (0 and 200 to 500 mg L^{-1}; one-way ANOVA). However, the highest concentrations seemed to affect the chl-*a* fluorescence, and the dose-response model was successfully fitted.

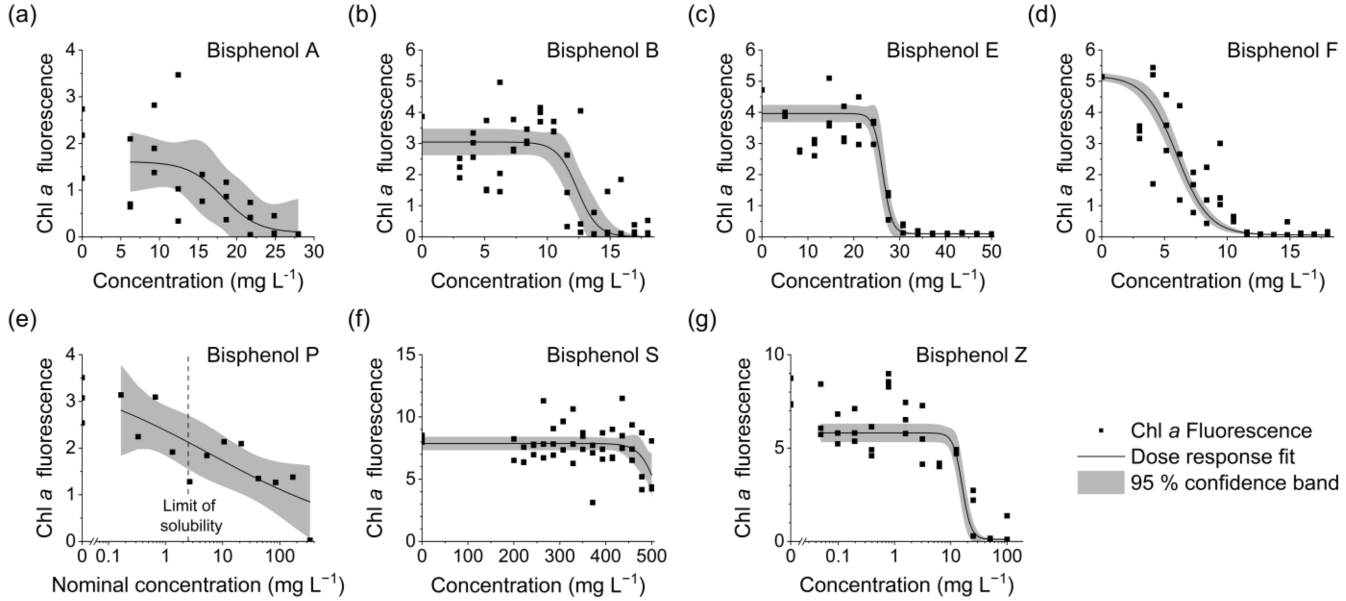

Figure S3. Toxicity of bisphenols for growing *Ulva mutabilis*. Chlorophyll-*a* fluorescence was fitted in a dose-response curve using independent biological replicates with four to seven specimens each. In alphabetical order: (**a**) bisphenol A, (**b**) bisphenol B, (**c**) bisphenol E, (**d**) bisphenol F, (**e**) bisphenol P, (**f**) bisphenol S, and (**g**) bisphenol Z decreased chlorophyll-*a* fluorescence. Bisphenol P was only soluble up to 2.5 mg L^{-1}. Bisphenol A data and fit were taken from previous publications for comparison.[1]

Comparing the toxicity towards *Ulva mutabilis*, the bisphenols ranged from an EC_{50} of 6.0 mg L^{-1} for BPF to a no-effect concentration of 500 mg L^{-1} for BPS and were ordered with increasing toxicity (**Table S6**): BPS << BPE < BPA < BPZ < BPB < BPF (≈ BPP). The curve steepness or

relative range of increasing effect differed quite drastically and was ordered with increasing steepness: from BPF < BPZ < BPA < BPB < BPE. BPP and BPS were not included due to low solubility or toxicity.

Table S6. Fit parameters and derived effective concentrations for dose-response fits of bisphenol toxicity. EC values are given as mean ± 95 % confidence intervals. Bottom asymptotes of bisphenol B, P, and S were set to 0 for improved fitting. Bisphenol P and Z curves were derived from geometric dilution series, which was logarithmized for fitting, while the other curves were derived from arithmetic dilutions. Therefore, these groups cannot directly compare inflection points and hill slopes.

	Bisphenol A	Bisphenol B	Bisphenol E	Bisphenol F	Bisphenol P	Bisphenol S	Bisphenol Z
**Bottom asymptote**	0.08504	0	0.10015	0.06075	0	0	0.1106
**Top asymptote**	1.61113	3.04708	3.9619	5.17454	3.74192	7.86938	5.81126
**Inflection point (mg L^{-1})**	18.24714	12.38789	26.31859	5.98875	0.78531	514.59788	1.20249
**Hill slope (L mg^{-1})**	-0.19063	-0.53164	-0.4384	-0.3404	-0.30574	-0.02192	-5.48007
**EC_{10} (mg L^{-1})**	13.2 ± 13.5	10.6 ± 2.1	24.1 ± 2.0	3.2 ± 1.0	0.08 ± 0.1	471 ± 41	10.7 ± 3.1
**EC_{20} (mg L^{-1})**	15.1 ± 9.5	11.3 ± 1.5	24.9 ± 1.6	4.2 ± 0.8	0.1 ± 1.5	487 ± 23	12.4 ± 2.9
**EC_{50} (mg L^{-1})**	18.2 ± 5.7	12.4 ± 0.9	26.3 ± 1.1	6.0 ± 0.5	6 ± 84	515 ± 37	15.9 ± 2.8
**EC_{80} (mg L^{-1})**	21.4 ± 9.9	13.5 ± 1.3	27.7 ± 1.0	7.8 ± 0.4	568 ± 3088	542 ± 76	20.5 ± 3.8
**EC_{90} (mg L^{-1})**	23.3 ± 13.8	14.2 ± 1.9	28.5 ± 1.2	8.8 ± 0.5	8061 ± 40679	558 ± 101	23.8 ± 5.3

2.2. Removal kinetics of BPA with different morphotypes and conditions

We compared the removal of BPA by different *U. mutabilis* morphotypes (slender: strain FSU-UM5-1 and wildtype: strain FSU-UM1-41) and conditions (**Fig. S4**). The tripartite community of the morphotypes slender and wildtype rapidly removed BPA. Both curves indicate a removal with first-order kinetics with a half-time as small as 1.85 h with circa 0.1 mg BPA per 1 g of fresh weight or 80 mg dry weight of *Ulva*. The removal was significantly slower in the absence of light and for callus-shaped *Ulva* with half-times of 14.6 h and 25.8 h, respectively. With autoclaved *Ulva*, the concentration followed a Langmuir isotherm instead of first-order kinetics, indicating adsorption to the inactive tissue. Living *Ulva* can likely adsorb BPA in a similar matter in addition to uptake and transformation, as discussed before.

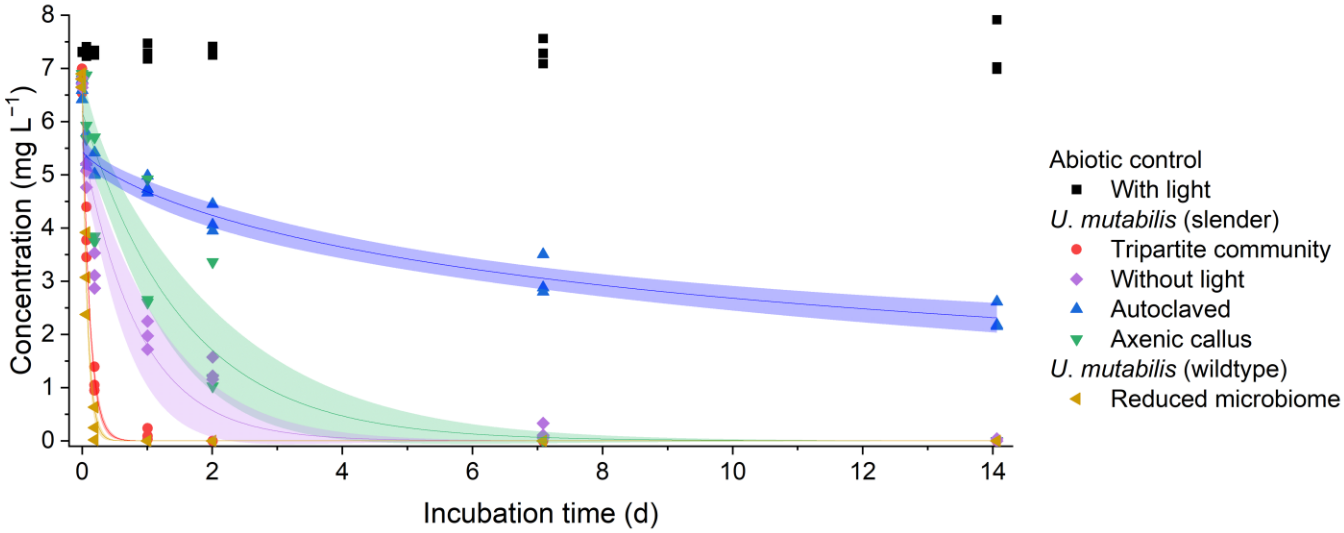

Figure S4. Change of concentration of bisphenol A under different conditions. Conditions include incubation under abiotic conditions (black), with *U. mutabilis* (slender) in a tripartite community with light (red), without light (violet), autoclaved (blue), axenic *U. mutabilis* (slender) in callus shape (green), and *U. mutabilis* (wildtype) (yellow). Symbols represent the results of individual biological replicates, and curves were fitted with a 95 % confidence interval. Biotic conditions were fitted with first-order reaction curves, while autoclaved *U. mutabilis* (slender) was fitted with a Langmuir isotherm, which was the preferred fit after Akaike and Bayesian information criteria. The wildtype morphotype of *U. mutabilis* was associated with a reduced (core) microbiome.

2.3. Transformation products of BPA

Table S7. Transformation products of BPA detected by GC-EI-HRMS. Proposed structures, retention times and quantifying ions. Masses of quantifying ions refer to the trimethylsilyl and O-methyl-oxime derivatives. *The GC-column was shortened between measurements. Therefore, the retention times of marked compounds are likely 5-7 seconds shorter compared to the others. **Structures proposed due to exact mass and isotopic abundance of one fragment. No confirmation of the structure is available.

Molecular formula	Proposed structure (IUPAC)	Short name (abbreviation)	RT (min)	Quantifying ion (*m*/*z*)	Type
**Detected by $X^{13}CMS$**					
$C_9H_{10}O$	4-(prop-1-en-2-yl)phenol	Propenylphenol (PP)	9.70	206.11269	$M^{+\bullet}$
$C_{10}H_{14}O_2$	4-(2-methoxypropan-2-yl)phenol	Methoxypropanylphenol (MPP)	11.43	223.11488	$(M-CH_3)^+$
$C_{11}H_{16}O_2$	4-(2-ethoxypropan-2-yl)phenol	Ethoxypropanylphenol (EPP)	12.17	237.13053	$(M-CH_3)^+$
$C_9H_{12}O_2$	4-(2-hydroxypropan-2-yl)phenol	Hydroxypropanylphenol (HPP)	13.54	281.13876	$(M-CH_3)^+$
$C_8H_8O_2$	1-(4-hydroxyphenyl)ethan-1-one	Hydroxyacetophenone (HAP)	14.32	237.11796	$M^{+\bullet}$
$C_9H_{11}BrO_2$	2-bromo-4-(2-hydroxypropan-2-yl)phenol	Bromohydroxypropanylphenol (BrHPP)	17.94	359.04927	$(M-CH_3)^+$
$C_9H_{10}Br_2O_2$	2,6-dibromo-4-(2-hydroxypropan-2-yl)phenol	Dibromohydroxypropanylphenol (Br_2HPP)	22.10	438.95774	$(M-CH_3)^+$
$C_{15}H_{15}BrO_2$	2-bromo-4-(2-(4-hydroxyphenyl)propan-2-yl)phenol	Bromobisphenol A (BrBPA)	30.74	435.08057	$(M-CH_3)^+$
$C_{15}H_{15}IO_2$	4-(2-(4-hydroxyphenyl)propan-2-yl)-2-iodophenol	Iodobisphenol A (IBPA)	32.16	483.06670	$(M-CH_3)^+$
$C_{15}H_{14}Br_2O_2$	2,6-dibromo-4-(2-(4-hydroxyphenyl)propan-2-yl)phenol	3,5-Dibromobisphenol A (3,5-Br_2BPA)	33.69	514.98904	$(M-CH_3)^+$
$C_{15}H_{14}Br_2O_2$	4,4'-(propane-2,2-diyl)bis(2-bromophenol)	3,3'-Dibromobisphenol A (3,3'-Br_2BPA)	33.75	514.98904	$(M-CH_3)^+$
$C_{21}H_{20}O_3$	4-(2-(4-(4-hydroxyphenoxy)phenyl)propan-2-yl)phenol	Bisphenol A-hydroquinone ether (BPA-HQ)	37.05	449.19627	$(M-CH_3)^+$
$C_{21}H_{19}BrO_3$	-	Brominated BPA-HQ	38.65	527.10679	$(M-CH_3)^+$
**Traces (found by direct search in some samples)**					
$C_{15}H_{14}BrIO_2$	2-bromo-4-(2-(4-hydroxyphenyl)propan-2-yl)-6-iodophenol**	3-Bromo,5-iodobisphenol A (3-Br,5-IBPA)	34.76	560.97722	$(M-CH_3)^+$
$C_{15}H_{14}BrIO_2$	2-bromo-4-(2-(4-hydroxy-3-iodophenyl)propan-2-yl)phenol**	3-Bromo,3'-iodobisphenol A (3-Br,3'-IBPA)	34.80	560.97722	$(M-CH_3)^+$
$C_{15}H_{13}Br_3O_2$	2,6-dibromo-4-(2-(3-bromo-4-hydroxyphenyl)propan-2-yl)phenol	Tribromobisphenol A (Br_3BPA)	35.86*	592.90057	$(M-CH_3)^+$
$C_{15}H_{12}Br_4O_2$	4,4'-(propane-2,2-diyl)bis(2,6-dibromophenol)	Tetrabrombisphenol A (Br_4BPA)	37.70*	672.81020	$(M-CH_3)^+$

Table S8. Transformation products of BPA detected by LC-MS. Proposed structures, retention times, and quantifying ions. *Most likely an isomeric mixture with different positions of bromine atoms in the ortho position of the phenol group, not separated in LC.

Molecular formula	Proposed structure (IUPAC)	Short name (abbreviation)	RT (min)	Quantifying ion (*m*/*z*)	Type
**Detected by X^{13}CMS and LC metabolomics method**					
$C_{15}H_{16}O_5S$	4-(2-(4-hydroxyphenyl)propan-2-yl)phenyl hydrogen sulfate	Bisphenol A bisulfate	4.39	307.06457	$(M-H)^-$
$C_{15}H_{15}BrO_5S$	*	Bromobisphenol A bisulfate	4.62	384.97508	$(M-H)^-$
**Detected by targeted search and LC quantification method**					
$C_{15}H_{15}BrO_2$	2-bromo-4-(2-(4-hydroxyphenyl)propan-2-yl)phenol	Bromobisphenol A (BrBPA)	7.76	305.01827	$(M-H)^-$
$C_{15}H_{14}Br_2O_2$	*	Dibromobisphenol A (Br_2BPA)	7.41	384.92673	$(M-H)^-$
$C_{15}H_{13}Br_3O_2$	*	Tribromobisphenol A (Br_3BPA)	7.72	462.83724	$(M-H)^-$
$C_{15}H_{16}O_5S$	4-(2-(4-hydroxyphenyl)propan-2-yl)phenyl hydrogen sulfate	Bisphenol A bisulfate	5.98	307.06457	$(M-H)^-$
$C_{15}H_{15}BrO_5S$	*	Bromobisphenol A bisulfate	6.50	384.97508	$(M-H)^-$
$C_{15}H_{14}Br_2O_5S$	*	Dibromobisphenol A bisulfate	6.13	464.88355	$(M-H)^-$

2.4. Time course of transformation products

After identifying transformation products, samples and data of the kinetics experiments were reevaluated (**Fig. S5-7**). The first-order consecutive reversible reaction kinetics model fitted every transformation product quite well.

As described for the bioaccumulation experiment, the area of accumulated bisphenol A (**Fig. S5g**) followed the course of bisphenol A in the medium (**Fig. S6a**) except at the beginning, where it increased quickly. The peak area of every transformation product varied over time. Propenylphenol (**Fig. S5a**), iodobisphenol A (**Fig. S5i**), and bisphenol A-hydroquinone ether (**Fig. S5l**) followed a similar time course than bisphenol A with a quick increase in the first hours followed by a decrease over approximately 4 days. The areas of methoxypropanylphenol (**Fig. S5b**) and ethoxypropanylphenol (**Fig. S5c**) had a comparable increase but decreased slowly, not quite reaching equilibrium after 16 days. While the areas of hydroxypropanylphenol (**Fig. S5d**), bisphenol A bisulfate (**Fig. S6b**) and bromobisphenol A bisulfate (**Fig. S6c**) also increased quickly within hours, the area kept increasing afterward with a slower rate. Although the areas of all brominated transformation products varied between replicates, they tended to increase over several days, with some not reaching a maximum within the time frame of the experiment.

Comparing the areas of all GC analytes (**Fig. 5n and 5o**), accumulated BPA had the highest area by a considerable degree. The area of hydroxypropanylphenol and bromobisphenol A was two orders of magnitude lower, while the area of the other transformation products was three or more orders of magnitude lower. Although the concentrations of the transformation products were unknown, a proportional relationship between the peak areas and concentration could be assumed within a reasonable margin. Since the structure and ionization of all GC analytes but propenylphenol were quite similar, their peak areas are somewhat comparable. This thorough comparison of GC analytes, which were structurally similar, provides a strong basis for our findings. Therefore, the low areas of all transformation products compared to accumulated BPA, which was below 1 % of the added amount, suggest that they were either intermediate products of a degradation pathway or minor products of low relevance.

Although it is theoretically possible that extraction and storage with ethanol and methanol yields their adducts as artifacts, the different time course of hydroxypropanylphenol and its ethers (methoxy- and ethoxypropanylphenol) indicated that the ethers were generated and metabolized before extraction.

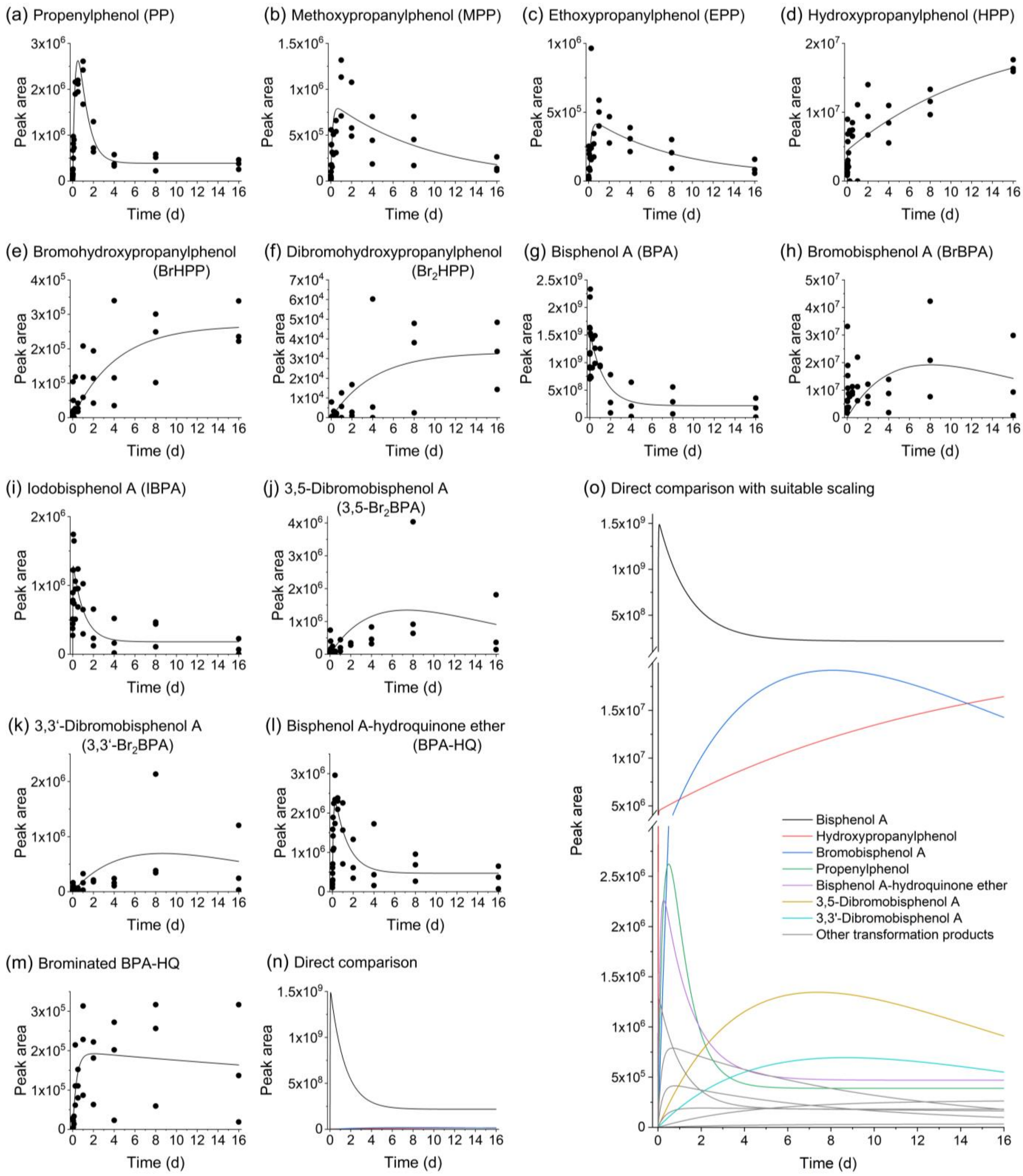

Figure S5. Peak area of BPA and its transformation products in algal tissue. Change over time was fitted with first order consecutive reversible reaction kinetics model. Despite the high biological variation observed in some samples, the model demonstrated an excellent fit. Compounds were detected with GC/MS and were ordered by retention time (a-m). Model fits all compounds for comparison (n-o).

When comparing the areas of all LC/MS analytes (**Fig. 6d**), the area of bisphenol A bisulfate reached about 12 % of the starting area of BPA. Since the pK_a of bisulfates was substantially lower than that of phenols, we expected distinctly higher ionization efficiency in the negative mode for bisphenol A bisulfate than BPA. Therefore, the peak areas of these analytes could not be compared directly, and the actual concentration of bisphenol A bisulfate was likely to be considerably lower than 12 % of the initial BPA concentration. Although a calibration with a synthesized standard would be necessary to calculate a concentration of bisphenol A bisulfate, we estimated its concentration in the medium to be negligibly small compared to the added amount of bisphenol A.

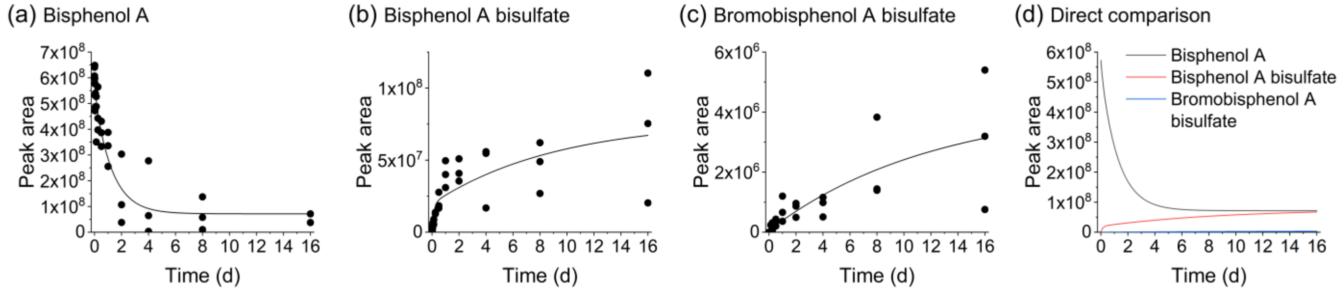

Figure S6. Peak area of BPA and its transformation products in the culture medium. Change over time was fitted with a first-order consecutive reversible reaction kinetics model. Compounds were detected with LC/MS (a-c) and a comparison of all curves (d).

The data of the removal kinetics experiment with different morphotypes and conditions was also reevaluated (**Fig. S7**). In this experiment, the starting concentration of BPA was half compared to the uptake experiment (**Fig. S7a**). Therefore, the resulting peak area of BPA at the start was smaller but not precisely half, presumably due to ion suppression effects. This effect was considered using isotopically labeled internal standards and area ratios for calculating concentrations. Nevertheless, peak areas were adequate for assessing the temporal progression of transformation products. Compared to the uptake and isotope label experiment, a targeted search of transformation products yielded higher peak areas for bisphenol A bisulfate (**Fig. S7e**) and bromobisphenol A bisulfate (**Fig. S7f**) and dibromobisphenol A bisulfate was identified as an additional transformation product (**Fig. S7g**). Additionally, mono- (**Fig. S7b**), di- (**Fig. S7c**), and tribromobisphenol A (**Fig. S7d**), identified by GC/MS, were detected in the medium. This was possible because all transformation products were more abundant in cultures incubated without light.

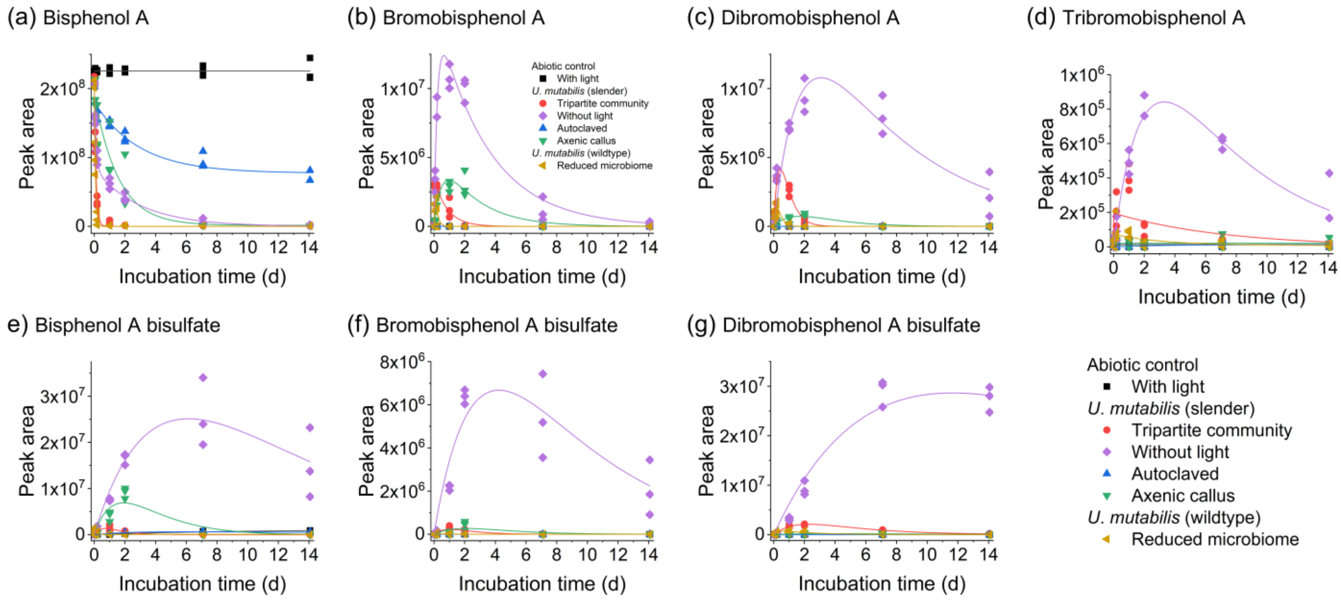

Figure S7. Peak area of BPA and its transformation products in the culture medium. Change over time was fitted with a first-order consecutive reversible reaction kinetics model. Compounds were detected with LC/MS for different conditions. The wildtype morphotype of *U. mutabilis* was associated with a reduced (core) microbiome.

3. Transformation products of bisphenol A

$X^{13}CMS$ automatically detected mass traces (features) of labeled transformation products. Co-eluting identified features were manually verified and deconvoluted into mass spectra (**Chapters 3-5**). The signal intensity of features was calculated as the mean area of 5 biological replicates. Features identified by $X^{13}CMS$ for unlabeled samples (black) were depicted head to tail with ^{13}C labeled samples (blue) or D labeled samples (red) to allow quick calculation of the number of labeled atoms. Some features, which were not automatically identified, were manually added to verify fragments or molecular ions (green). MS^2 spectra for LC data were measured for one mass peak (yellow).

Proposed structures and chemical formulas of unlabeled ions were depicted with theoretical mass (*m*/*z* for *z* = 1) and the relative difference (delta) between theoretical and experimental *m*/*z* in parts per million (ppm; equation 1):

$$\text{delta} = \left(\frac{\text{experimental}\, m/z}{\text{theoretical}\, m/z} - 1\right) \times 10^{6}\ \text{ppm} \qquad \text{(Equation 1)}$$

Labeled features of more abundant compounds often showed M-n peaks due to the automatic identification of incomplete labeling of the commercially available labeled standards (97 – 99 % isotopic purity). $X^{13}CMS$ also often detected natural ^{13}C M+1 peaks in the unlabeled samples for analysis of ^{13}C label. In a few cases, features of the unlabeled compound were also present in the labeled samples, presumably because the compound is naturally present in *Ulva*.

All proposed structures and their fragments take the number and position of labeled atoms into account: Every proposed structure carries the correct number of labeled atoms to be expected for the proposed biotransformation, and features of corresponding fragments carry the correct number of labeled atoms to be expected for the proposed fragmentation.

Proposed structures were confirmed by comparison with a spectrum library when available.

3.1. Example of structural elucidation

The structural elucidation can be explained with the example of bromobisphenol A (**Fig. S8**): Comparing the raw spectra of unlabeled (30.72 - 30.76 min), ^{13}C labeled (30.72 - 30.76 min), and D labeled samples (30.57 - 30.64 min; D labeled compounds revealed a shorter retention time[10]), 8 labeled mass peaks can easily be spotted at *m/z* 191, 207, 341, 357, 435, 437, 450, and 452.[*] With these masses, structural elucidation could be carried out in the usual manner[11] with the added benefit of the high mass resolution, knowledge of precursor structure, and the number of labeled atoms for each fragment. In the example, *m/z* 437 and 452 are ^{81}Br M+2 peaks of *m/z* 435 and 450, respectively. The ion *m/z* 450 was undoubtedly the molecular ion since it (a) had the highest mass and highest number of labels, (b) fulfills all requirements for an odd-electron ion, and (c) can yield the other ions by logical neutral losses as we will see later. Due to the number of labeled atoms, the molecular ion carried at least 12 carbon and 13 hydrogen atoms in addition to the identified singular bromine. Calculating molecular formula from the exact mass 450.10405 with elements present for the derivatized precursor BPA (carbon, hydrogen, oxygen, and silicon) plus the identified bromine (exactly one) resulted in $C_{21}H_{31}O_2BrSi_2$ (0.006 delta ppm) and $C_{22}H_{27}O_5Br$ (0.915 delta ppm) within the resolution of the instrument (5 delta ppm).[†] When comparing these molecular formulas to the derivatized precursor ($C_{21}H_{32}O_2Si_2$), $C_{21}H_{31}O_2BrSi_2$ was very similar and could result from a simple replacement of hydrogen with bromine (bromination). Therefore, brominated BPA was a logical structural proposal, which was confirmed with the fragments (**Fig. S8**). Derivatized BPA has 3 equivalent hydrogen atoms: ortho, meta on the phenol subdivision, and at the methyl group. The deuterium-labeled fragment of *m/z* 207 contained 10 deuterium atoms and, therefore, both fully labeled methyl groups, indicating that the bromine atom was located on the phenol ring lost during fragmentation. Since no fragments existed that allowed the differentiation of ortho and meta bromobisphenol A, the structure had to be confirmed via co-injection (see **chapter 3.9**).

---

[*] X^{13}CMS immensely aided this step since it automatically identified labeled features over the whole retention time, even at low abundance.

[†] Of course, other organic elements might also have been added by biotransformation or derivatization (nitrogen is added by derivatization of aldehydes or ketones). These were added in an expanding search to calculate the molecular formula, whenever the smaller list of elements proved insufficient.

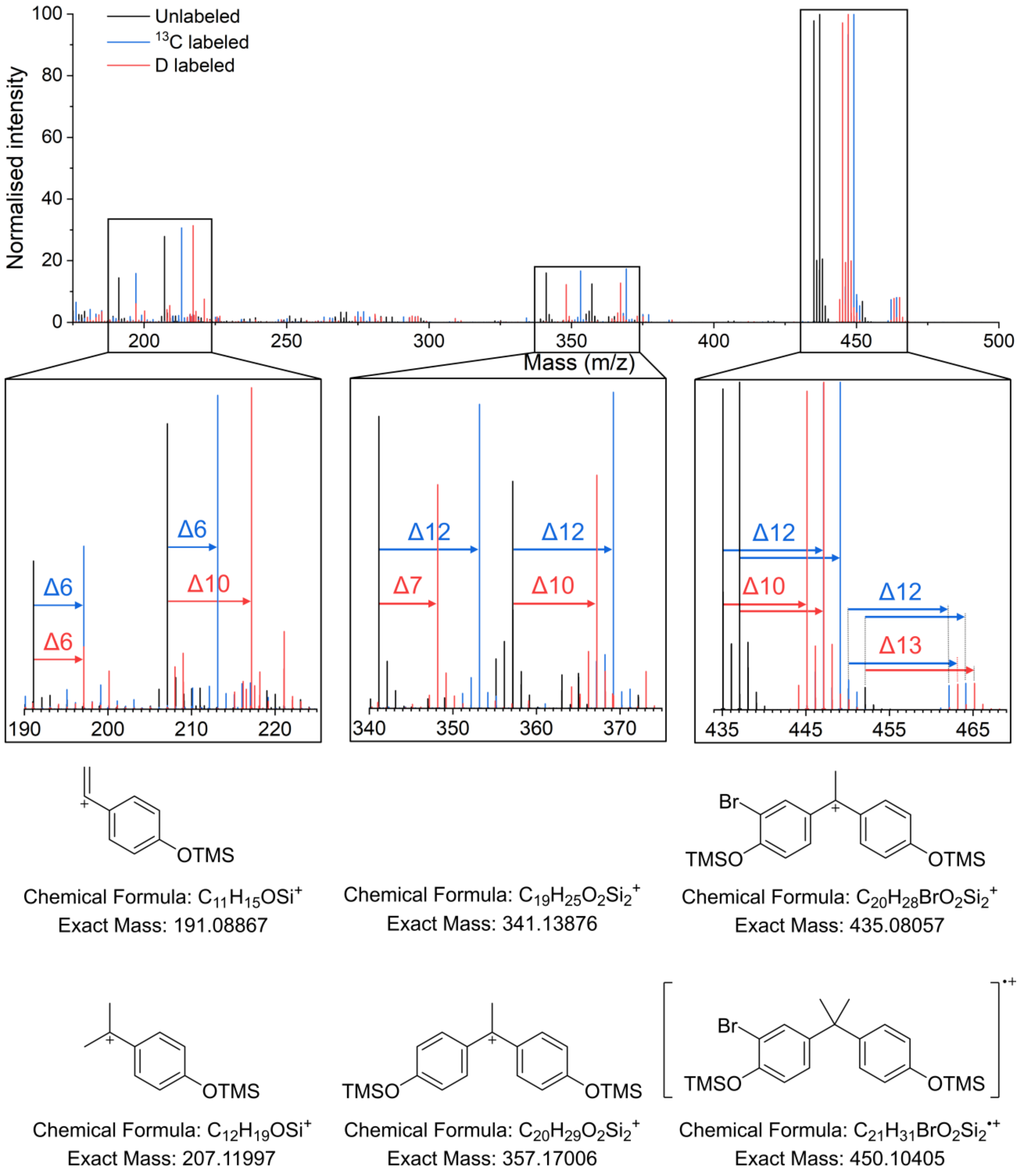

Figure S8. Example of structural elucidation using bromobisphenol A. Raw spectra of unlabeled, D-labeled, and ^{13}C-labeled samples with proposed (unlabeled) molecular ion and fragments.

3.2. **Propenylphenol (PP)**, rt 9.70 min, GC (derivatized)

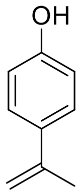

4-(prop-1-en-2-yl)phenol

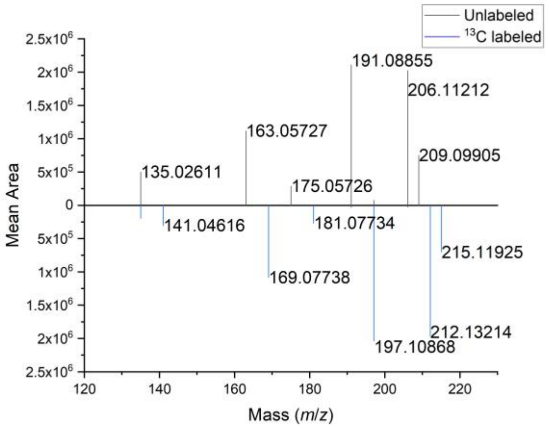

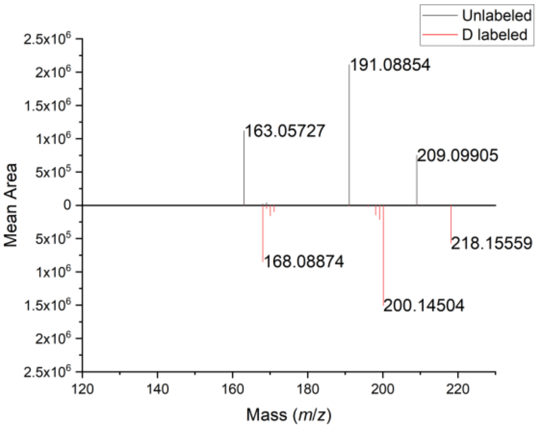

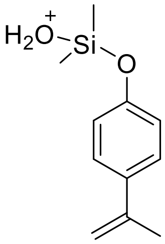

Chemical formula: $C_{11}H_{17}O_2Si^+$
Exact mass: 209.09923
Delta ppm: -0.874

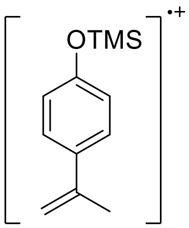

Chemical Formula: $C_{12}H_{18}OSi^{\bullet+}$
Exact Mass: 206.11214
Delta ppm: -0.113

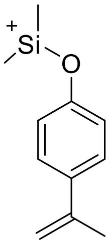

Chemical formula: $C_{11}H_{15}OSi^+$
Exact mass: 191.08867
Delta ppm: -0.618

$C_{10}H_{11}OSi^+$
Exact mass: 175.05737
Delta ppm: -0.617

$C_9H_{11}OSi^+$
Exact mass: 163.05737
Delta ppm: -0.601

$C_7H_7OSi^+$
Exact mass: 135.02607
Delta ppm: 0.312

Figure S9. Mass spectra and identified fragments of propenylphenol. The highest mass (*m/z* 209) resulted from the formation of water adducts of the $[M\text{-}CH_3]^+$ ion. This gas phase reaction is a known phenomenon in ion trap mass spectrometry.[12]

3.3. **Methoxypropanylphenol (MPP)**, rt 11.43 min, GC (derivatized)

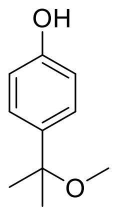

4-(2-methoxypropan-2-yl)phenol

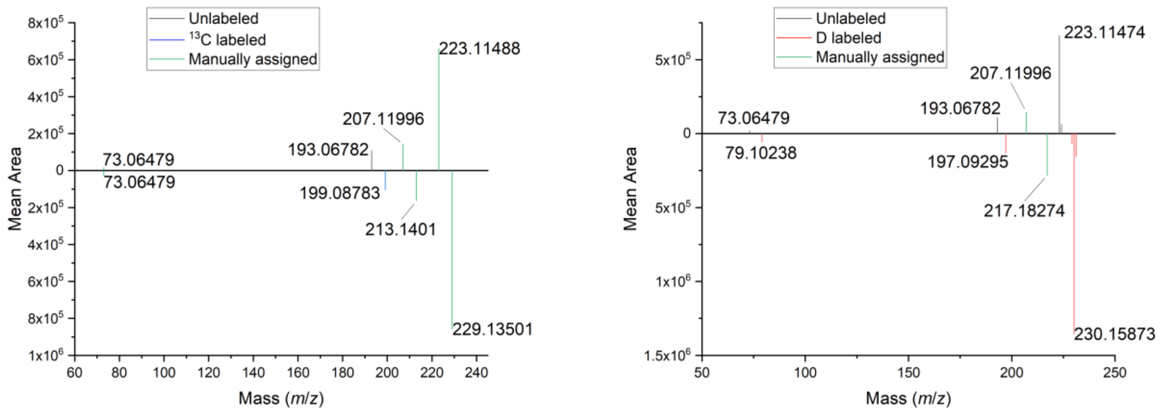

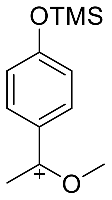

Chemical formula: $C_{12}H_{19}O_2Si^+$
Exact mass: 223.11488
Delta ppm: -0.020

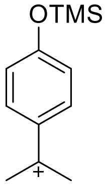

Chemical formula: $C_{12}H_{19}OSi^+$
Exact mass: 207.11997
Delta ppm: -0.584

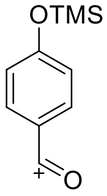

Chemical formula: $C_{10}H_{13}O_2Si^+$
Exact mass: 193.06793
Delta ppm: -0.040

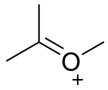

Chemical formula: $C_4H_9O^+$
Exact mass: 73.06479
Delta ppm: -0.013

Figure S10. Mass spectra and identified fragments of methoxypropanylphenol. The structural proposition was confirmed by the spectral library.[13]

3.4. **Ethoxypropanylphenol (EPP)**, rt 12.17 min, GC (derivatized)

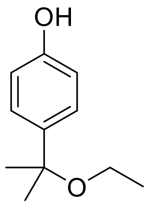

4-(2-ethoxypropan-2-yl)phenol

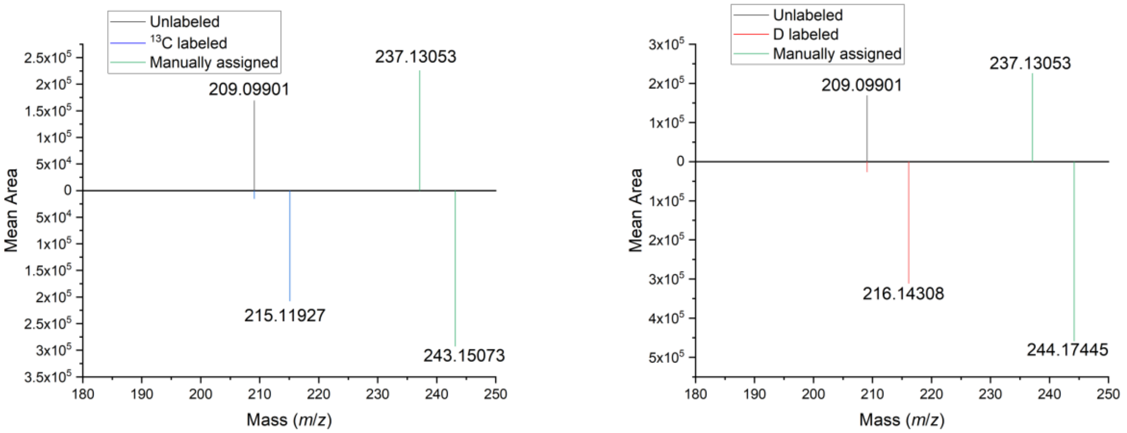

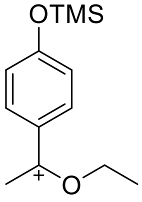

Chemical formula: $C_{13}H_{21}O_2Si^+$
Exact mass: 237.13053
Delta ppm: -0.358

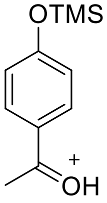

Chemical formula: $C_{11}H_{17}O_2Si^+$
Exact mass: 209.09923
Delta ppm: -1.066

Figure S11. Mass spectra and identified fragments of ethoxypropanylphenol.

3.5. **Hydroxypropanylphenol (HPP)**, rt 13.54 min, GC (derivatized)

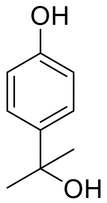

4-(2-hydroxypropan-2-yl)phenol

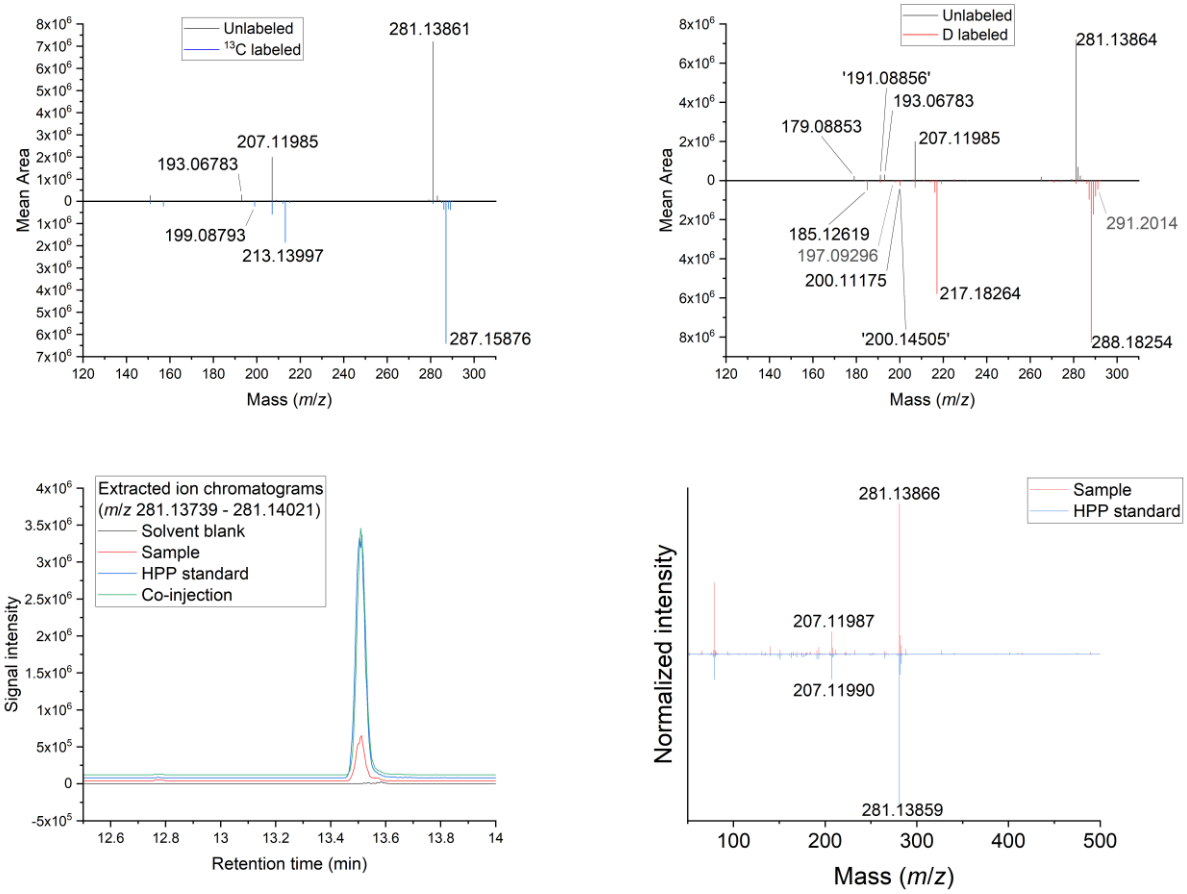

Figure S12: Mass spectra, and co-injection with mass spectra of hydroxypropanylphenol. X^{13}CMS identified two D-labeled fragments for the unlabeled *m/z* 281 and 193, depending on whether a labeled or unlabeled methyl group was fragmented, resulting in a mass difference of 3 nominal masses. The minority fragments are shown in grey. To differentiate fragment pairs (191, 200) and (193, 200), the former are marked. Structure was confirmed by co-injection. Due to column cutting, the co-injection measurements had a slightly shorter retention time compared to the labeling experiment.

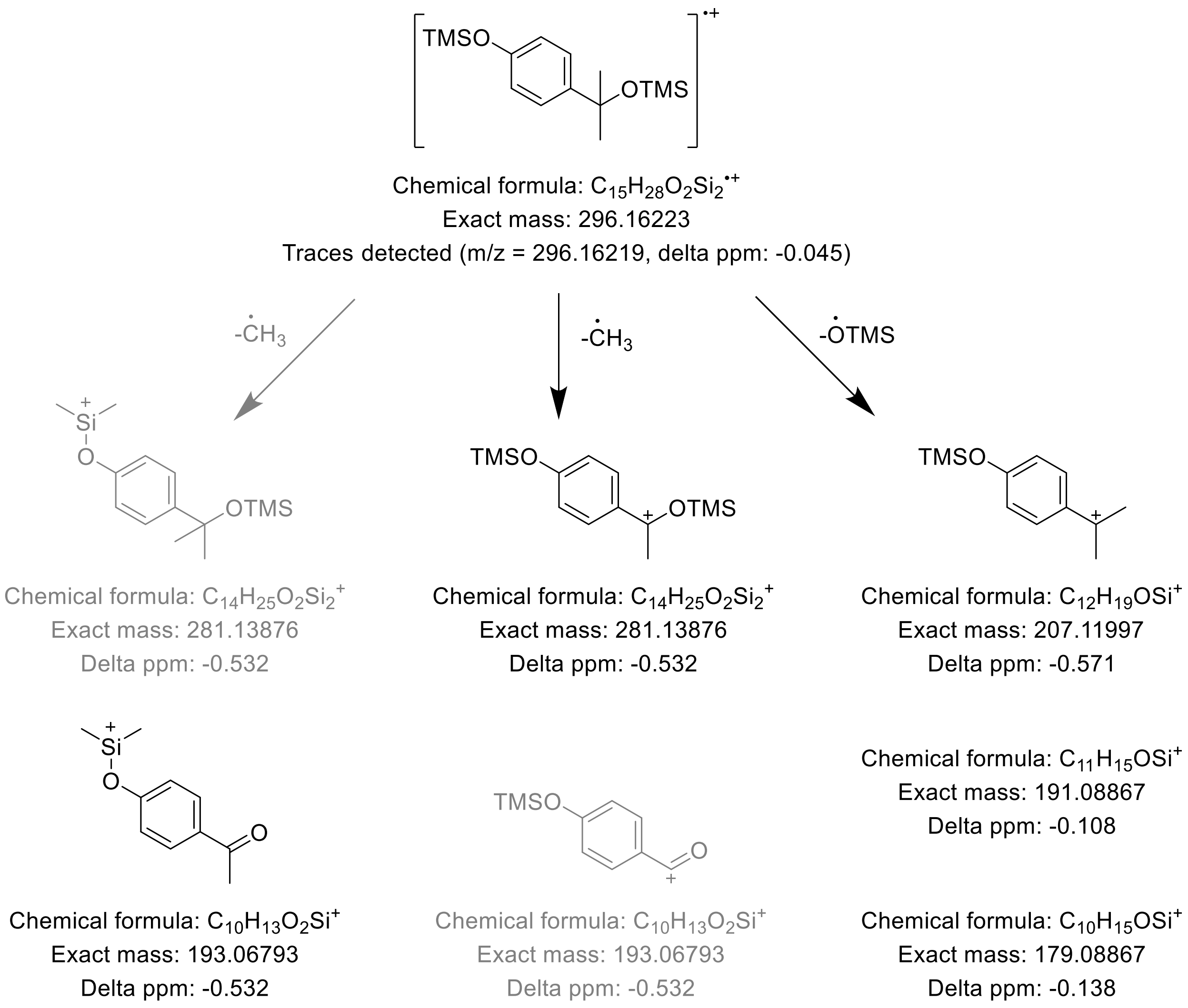

Figure S13: Identified fragments of hydroxypropanylphenol. X[13]CMS identified two D-labeled fragments corresponding to the unlabeled *m/z* 281 and 193. The presence of a labeled or unlabeled methyl group caused a mass difference of 3 nominal masses. The minority fragments are shown in grey. The targeted search successfully detected traces of the predicted molecular ion.

3.6. **Hydroxyacetophenone (HAP)**, rt 14.32 min, GC (derivatized)

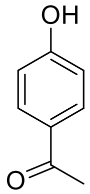

1-(4-hydroxyphenyl)ethan-1-one

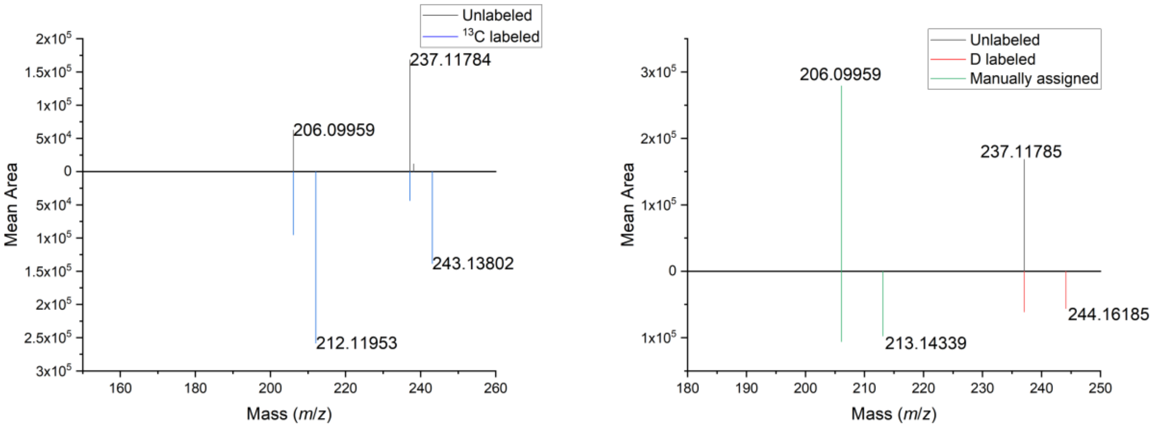

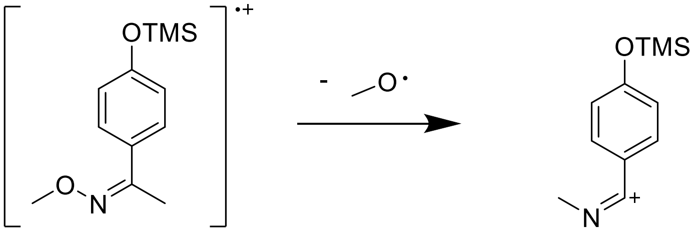

Chemical formula: $C_{12}H_{19}NO_2Si^{\bullet+}$
Exact mass: 237.11796
Delta ppm: -0.493

Chemical formula: $C_{11}H_{16}NOSi^{+}$
Exact mass: 206.09957
Delta ppm: 0.111

Figure S14. Mass spectra and identified fragments of 4-hydroxyacetophenone. The structural proposition was confirmed by the spectral library.[14]

3.7. **Bromohydroxypropanylphenol (BrHPP)**, rt 17.94 min, GC (derivatized)

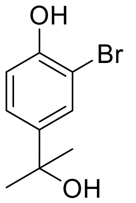

2-bromo-4-(2-hydroxypropan-2-yl)phenol

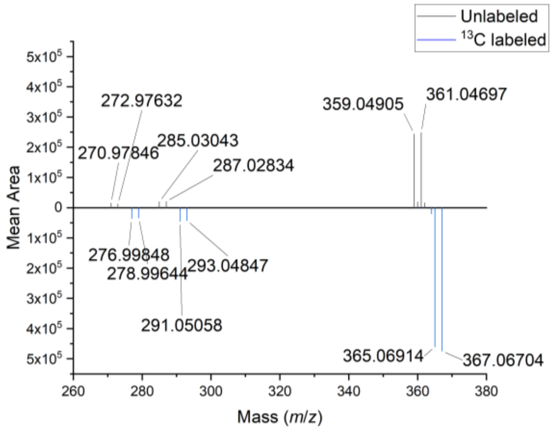

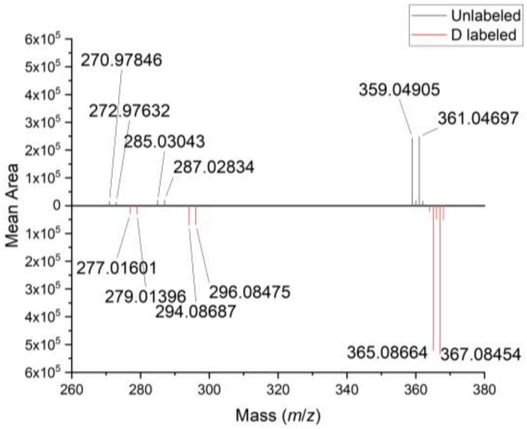

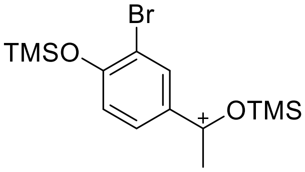

Chemical formula: $C_{14}H_{24}BrO_2Si_2^+$

Exact mass (abundance), delta of Br peaks:

^{79}Br: 359.04927 (100.0%), -0.619 ppm

^{81}Br: 361.04722 (97.3%), -0.708 ppm

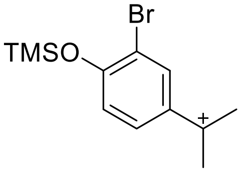

Chemical formula: $C_{12}H_{18}BrOSi^+$

Exact mass (abundance), delta of Br peaks:

^{79}Br: 285.03048 (100.0%), -0.178 ppm

^{81}Br: 287.02843 (97.3%), -0.328 ppm

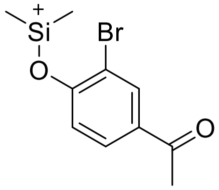

Chemical formula: $C_{10}H_{12}BrO_2Si^+$

Exact masses (abundance), delta of Br peaks:

^{79}Br: 270.97844 (100.0%), 0.054 ppm

^{81}Br: 272.97640 (97.3%), -0.288 ppm

Figure S15. Mass spectra and identified fragments of bromohydroxypropanylphenol. The presence of bromine atoms on the ring is indicated by spectra and number of deuterium-labeled atoms. The ortho position to the phenol oxygen is the most probable location for bromination, as evidenced by the bromination of the structurally similar compound bisphenol A at that position (see **Chapter 3.9**). Moreover, the ortho position is favored in electrophilic substitution reactions, which is the probable reaction mechanism for this bromination.

3.8. **Dibromohydroxypropanylphenol (Br2HPP)**, rt 22.10 min, GC (derivatized)

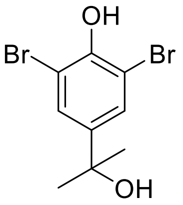

2,6-dibromo-4-(2-hydroxypropan-2-yl)phenol

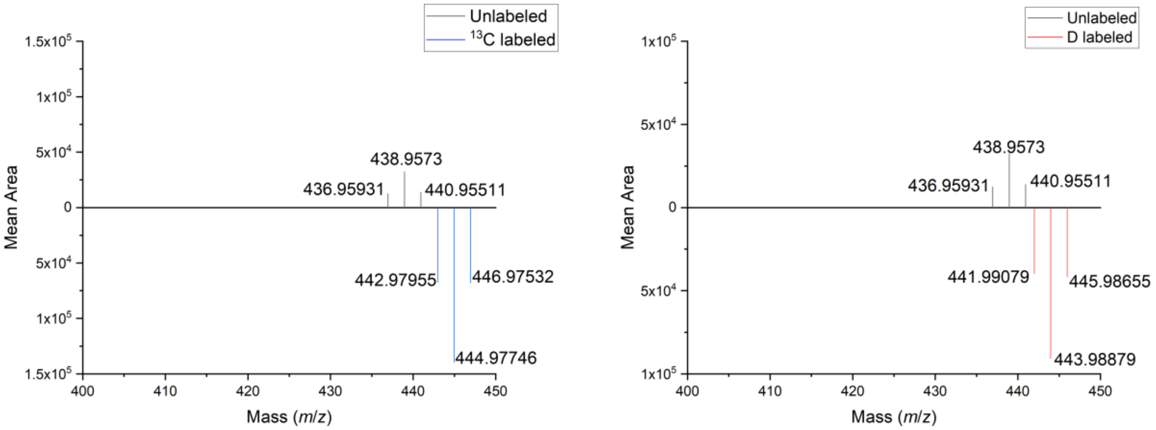

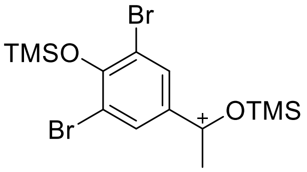

Chemical formula: $C_{14}H_{23}Br_2O_2Si_2^+$

Exact mass (abundance), delta of Br peaks:

$^{79}Br_2$: 436.95978 (51.4%), -1.086 ppm

$^{79}Br^{81}Br$: 438.95774 (100.0%), -0.998 ppm

$^{81}Br_2$: 440.95569 (48.6%), -1.319 ppm

Figure S16. Mass spectra and identified fragments of dibromohydroxypropanylphenol. The presence of bromine atoms on the ring is indicated by spectra and number of deuterium-labeled atoms. The ortho position to the phenol oxygen is the most probable location for bromination, as evidenced by the bromination of the structurally similar compound bisphenol A at that position (see **Chapters 3.9 and 3.11-3.14**). Moreover, the ortho position is favored in electrophilic substitution reactions, which is the probable reaction mechanism for this bromination.

3.9. **Bromobisphenol A (BrBPA)**, rt 30.74 min, GC (derivatized)

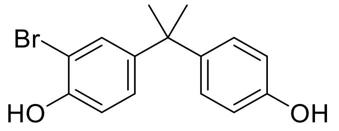

2-bromo-4-(2-(4-hydroxyphenyl)propan-2-yl)phenol

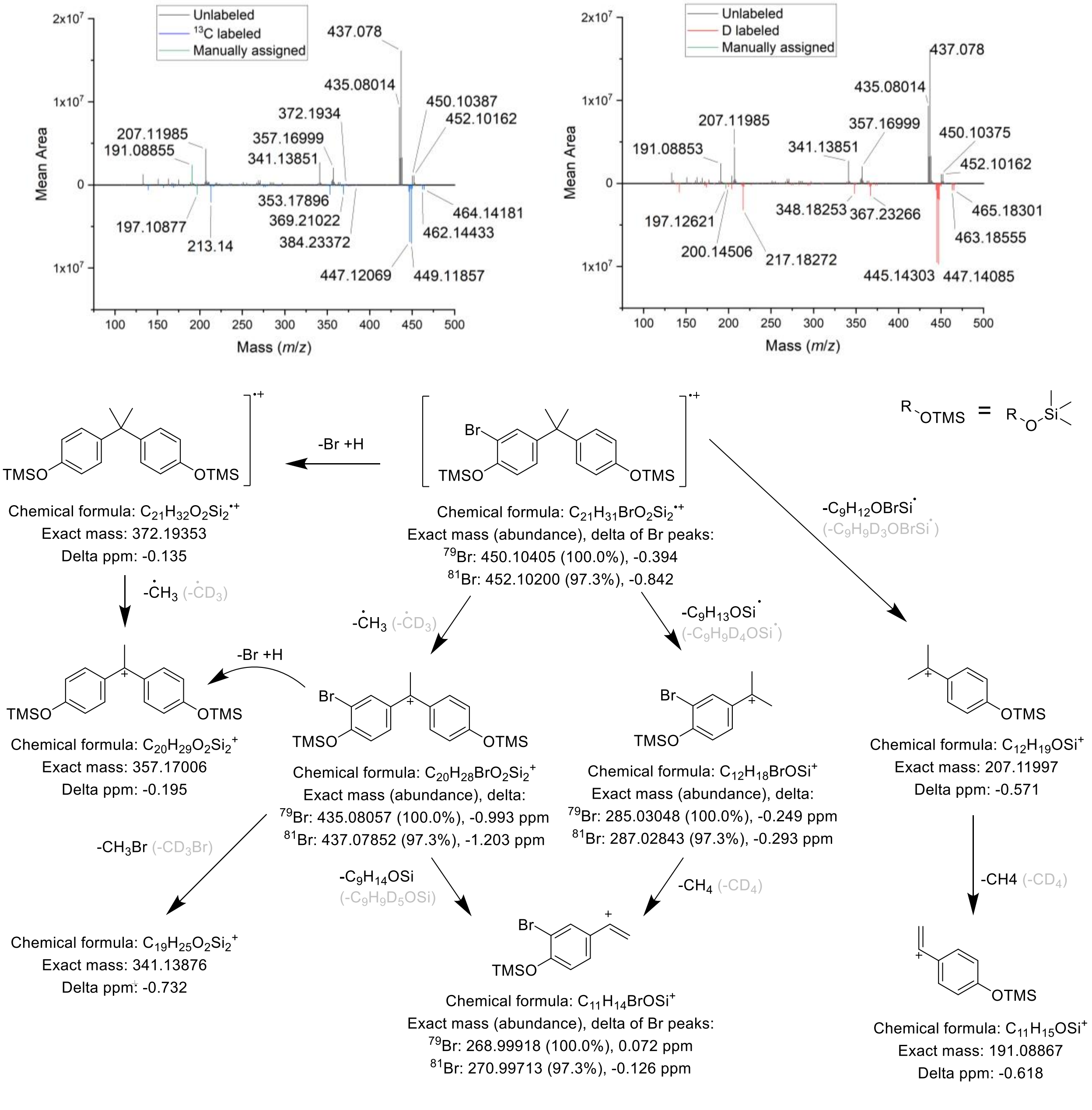

Figure S17. Mass spectra identified important fragments and fragmentation pathways of bromobisphenol A. Fragment losses for deuterium-labeled isotopologue are depicted in light grey.

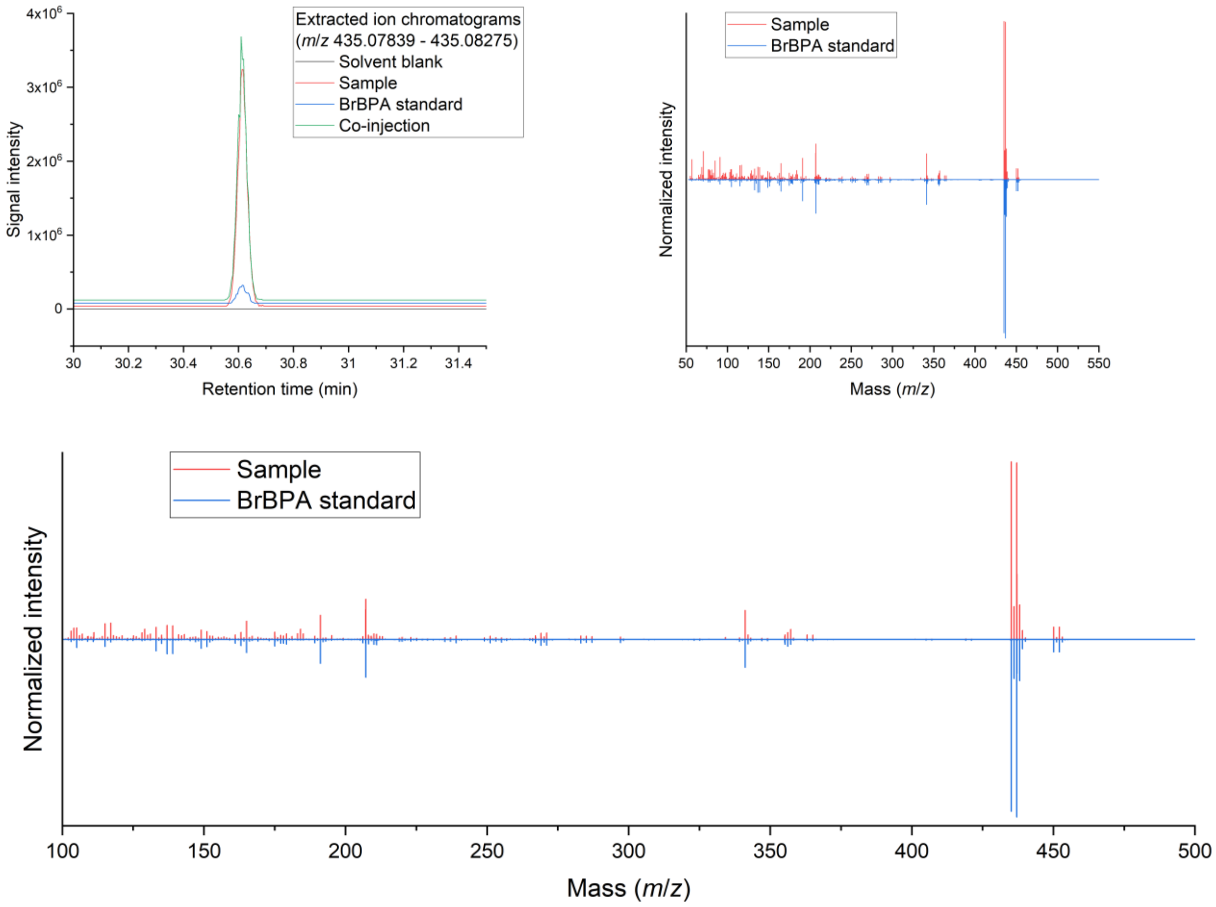

Figure S18: Identity of bromobisphenol A was confirmed through co-injection with mass spectra. The retention time of the co-injection (extracted ion chromatograms) was slightly shorter compared to the labeling experiment due to column cutting.

3.10. **Iodobisphenol A (IBPA)**, rt 32.16 min, GC (derivatized)

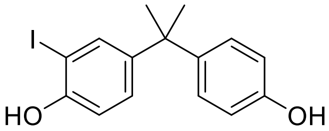

4-(2-(4-hydroxyphenyl)propan-2-yl)-2-iodophenol

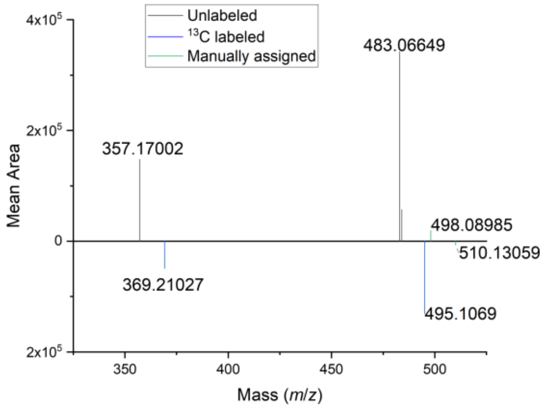

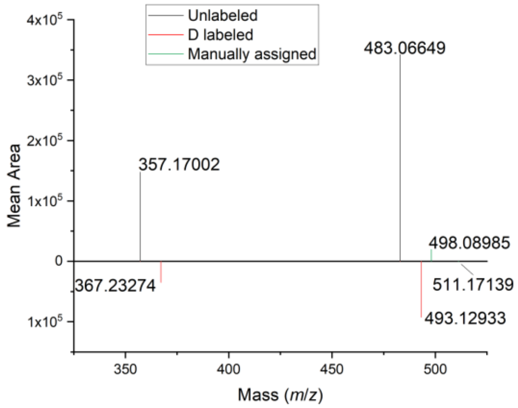

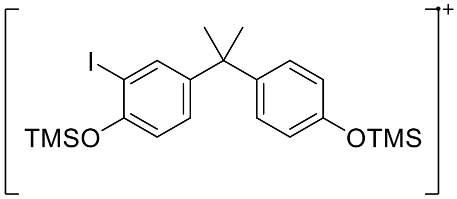

Chemical Formula: $C_{21}H_{31}IO_2Si_2^{\bullet+}$
Exact Mass: 498.09018
Delta ppm: -0.658

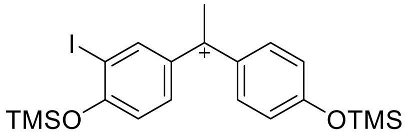

Chemical Formula: $C_{20}H_{28}IO_2Si_2^{+}$
Exact Mass: 483.06670
Delta ppm: -0.44

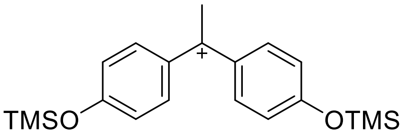

Chemical Formula: $C_{20}H_{29}O_2Si_2^{+}$
Exact Mass: 357.17006
Delta ppm: -0.111

Figure S19. Mass spectra and identified fragments of Iodobisphenol A. Spectra and the number of deuterium-labeled atoms indicate the presence of iodine atom on the ring. The ortho position to the phenol oxygen is the most probable location for iodination, as evidenced by the bromination of bisphenol A at that position (see **Chapter 3.9**). The most likely reaction mechanism for this iodination is an electrophilic substitution reaction with HOI as an electrophile. This reaction could be catalyzed by haloperoxidases (e.g., bromoperoxidase) and would favor the ortho position.

3.11. **3,5-Dibromobisphenol A (3,5-Br2BPA)**, rt 33.69 min, GC (derivatized)

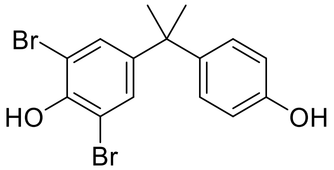

2,6-dibromo-4-(2-(4-hydroxyphenyl)propan-2-yl)phenol

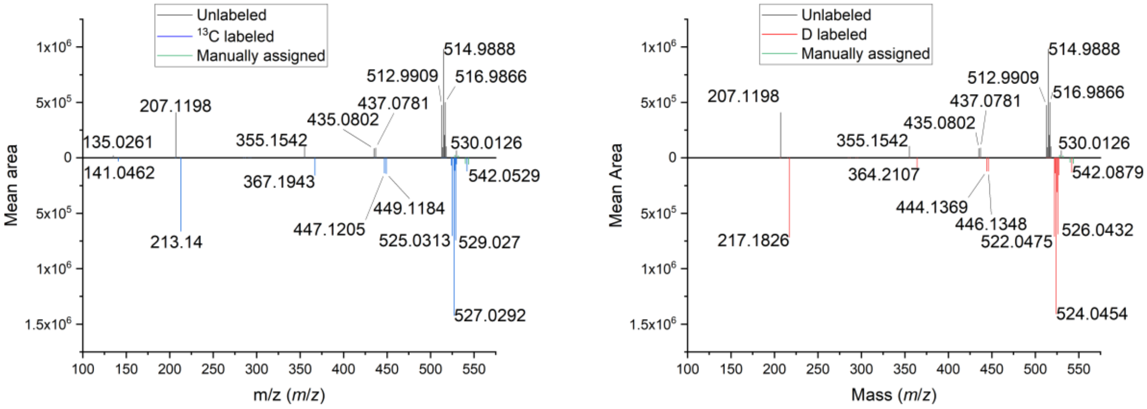

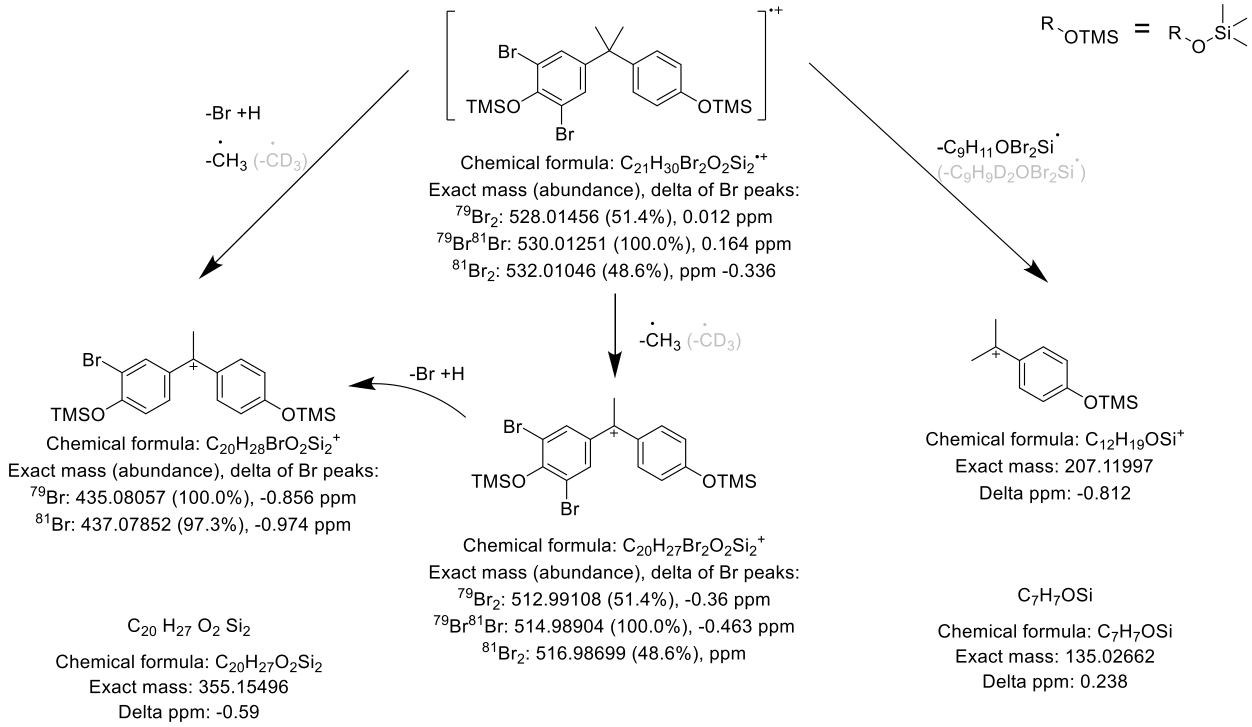

Figure S20. Mass spectra, identified important fragments, and fragmentation pathway of 3,5-dibromobisphenol A. Fragment losses for deuterium labeled isotopologue are depicted in light grey.

3.12. 3,3'-Dibromobisphenol A (3,3'-Br₂BPA), rt 33.75 min, GC (derivatized)

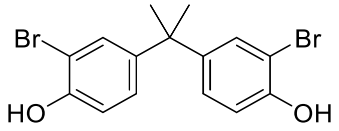

4,4'-(propane-2,2-diyl)bis(2-bromophenol)

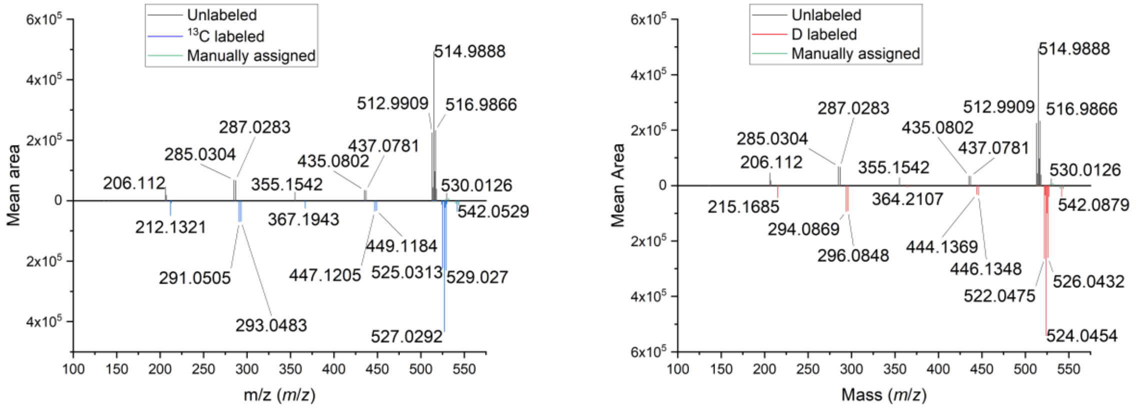

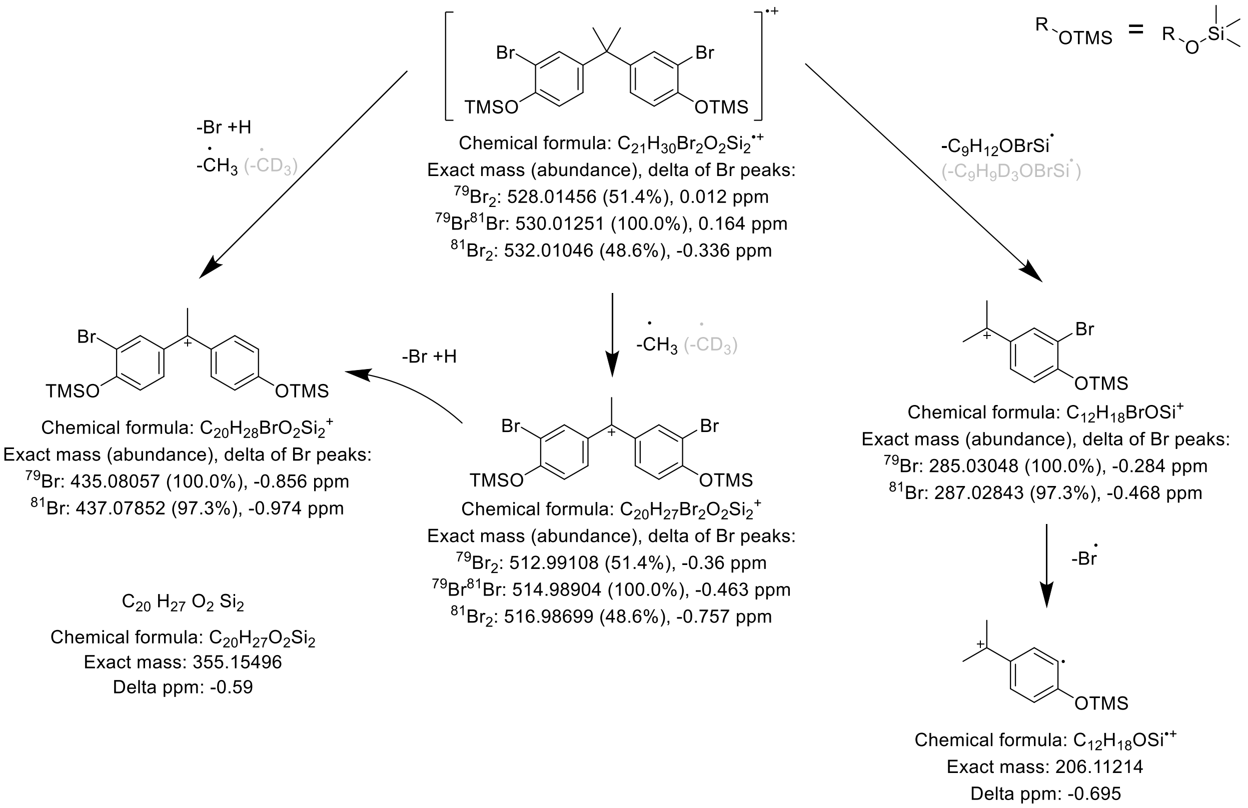

Figure S21. Mass spectra, identified important fragments, and fragmentation pathway of 3,3'-dibromobisphenol A. Fragment losses for deuterium labeled isotopologue are depicted in light grey.

3.13. **Comparison of isomers of dibromobisphenol A (Br_2BPA)**, GC (derivatized)

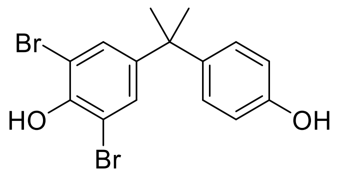

2,6-dibromo-4-(2-(4-hydroxyphenyl)propan-2-yl)phenol

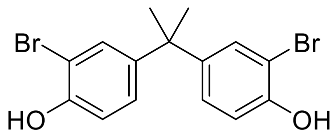

4,4'-(propane-2,2-diyl)bis(2-bromophenol)

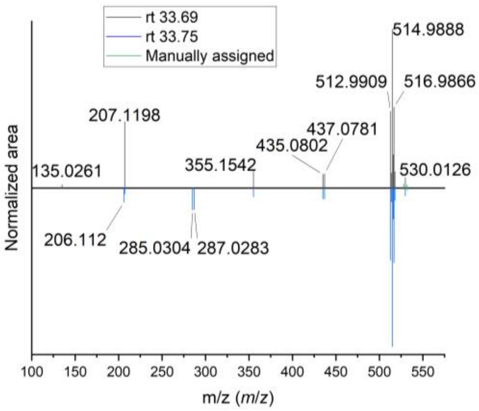

Figure S22: Spectra of 3,5 and 3,3'-dibromobisphenol A. The primary differentiating factors in the spectra were a markedly elevated *m/z* 207 peak at a retention time of 33.69 min, accompanied by the absence of the 206 and 285/287 peaks, which were only present at a retention time of 33.75 min. These observations suggest that the former compound possesses two bromine atoms at a single ring, whereas the latter carries one bromine atom per ring.

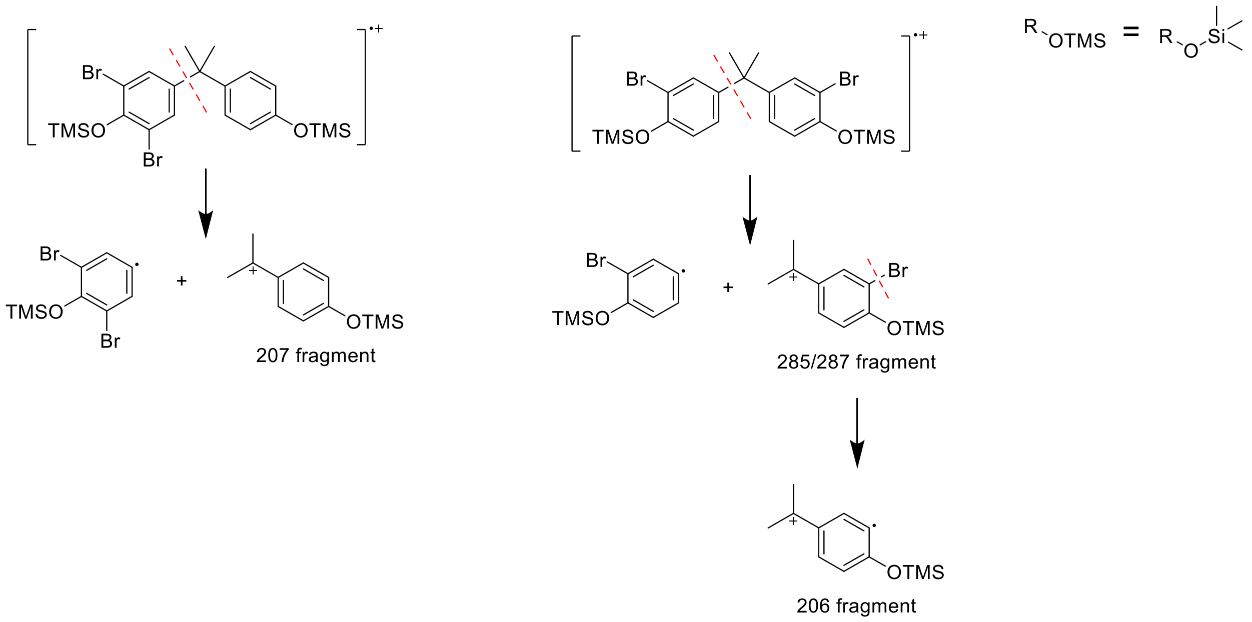

Figure S23: Fragmentation differences for dibromobisphenol A. Fragmentation resulted in a *m/z* 207 peak for the 3,5-isomer (rt 33.69) or *m/z* 206 and 285/287 peaks for the 3,3'-isomer (rt 33.75).

3.14. **Assignment of isomers of dibromobisphenol A (Br_2BPA)**, GC (derivatized)

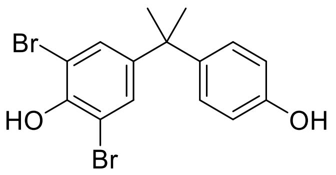

2,6-dibromo-4-(2-(4-hydroxyphenyl)propan-2-yl)phenol

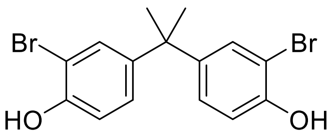

4,4'-(propane-2,2-diyl)bis(2-bromophenol)

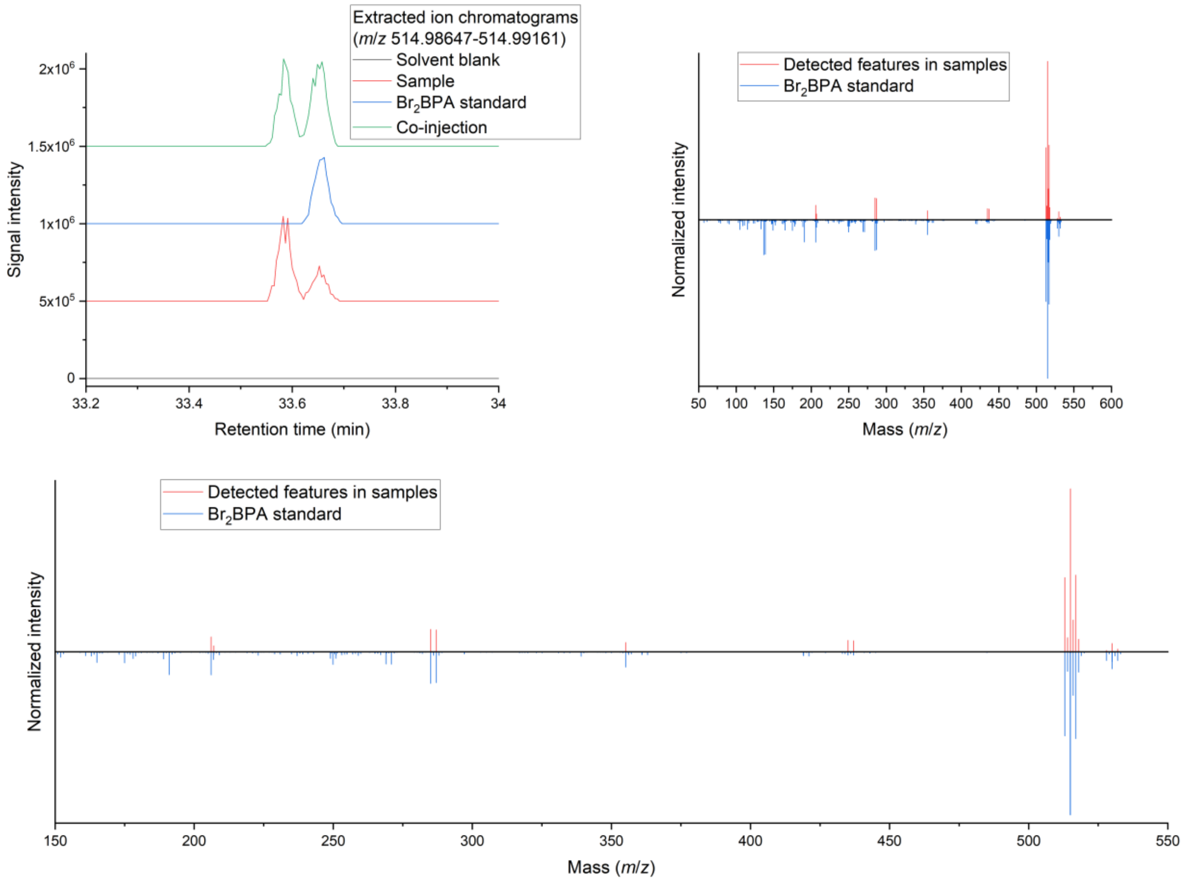

Figure S24: Co-injection and mass spectra of 3,3'-dibromobisphenol A standard and sample. Extracted ion chromatograms are drawn with an offset of 50000. They showed two peaks for the sample and one peak for the standard. The second peak of the sample co-eluted with the standard, which was confirmed with a co-injection. Therefore, the second peak of the sample was identified as 3,3'-dibromobisphenol A, while the first peak was determined to be 3,5-dibromobisphenol A. Additionally, the identity of 3,3'-dibromobisphenol A was confirmed through mass spectra. Due to the low abundance and matrix background, the spectrum displayed for the samples is the deconvoluted result of the labeling experiment (as depicted in **Chapter 3.12**), which differed in the relative abundance of some peaks in comparison to the raw spectrum of the standard for the same reasons. The retention time of the co-injection (extracted ion chromatograms) was slightly shorter than the labeling experiment due to column cutting.

3.15. **Isomers of bromoiodobisphenol A (BrIBPA)**, rt 34.76 and 34.80 min, GC (derivatized), targeted search

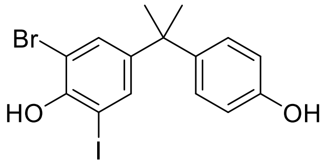

2-bromo-4-(2-(4-hydroxyphenyl)propan-2-yl)-6-iodophenol

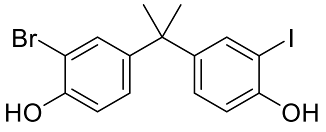

2-bromo-4-(2-(4-hydroxy-3-iodophenyl)propan-2-yl)phenol

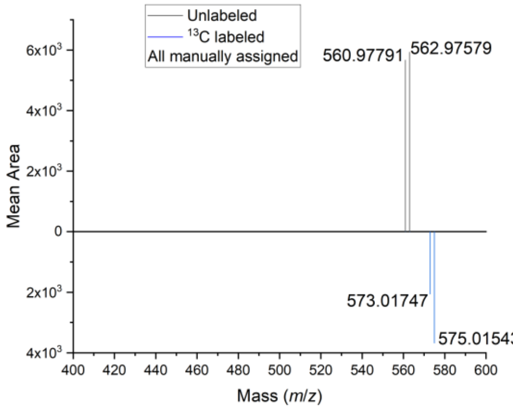

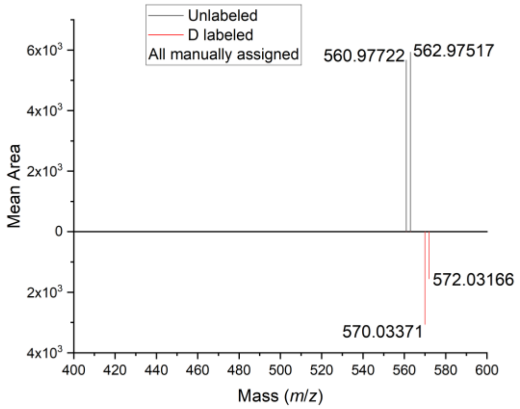

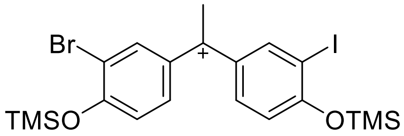

Chemical formula: $C_{20}H_{27}BrIO_2Si_2^+$
Exact mass (abundance), delta of Br peaks:
^{79}Br: 560.97722 (100.0%), 1.243 ppm
^{81}Br: 562.97517 (97.3%), 1.097 ppm

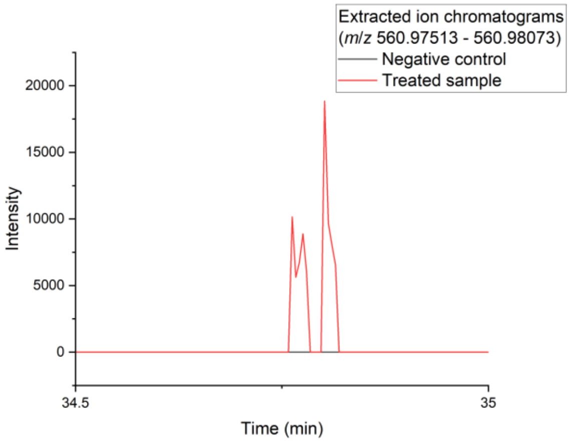

Figure S25. Proposed structure, mass spectra, identified fragments, and chromatogram of bromoiodobisphenol A. The predicted $[M\text{-}CH_3]^+$ ion was detected in several samples and corresponding isotopologues in the labeled samples. The chromatogram revealed two peaks, most likely representing isomers with halogens in the ortho position. Based on the retention time of dibromobisphenol A, the isomer with both halogens on one ring was expected to elute first.

3.16. **Polybrominated bisphenol A**, GC (derivatized), targeted search

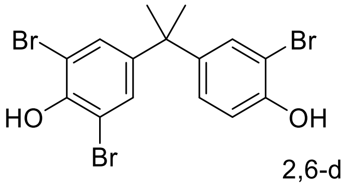

2,6-dibromo-4-(2-(3-bromo-4-hydroxyphenyl)propan-2-yl)phenol

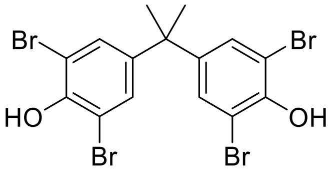

4,4'-(propane-2,2-diyl)bis(2,6-dibromophenol)

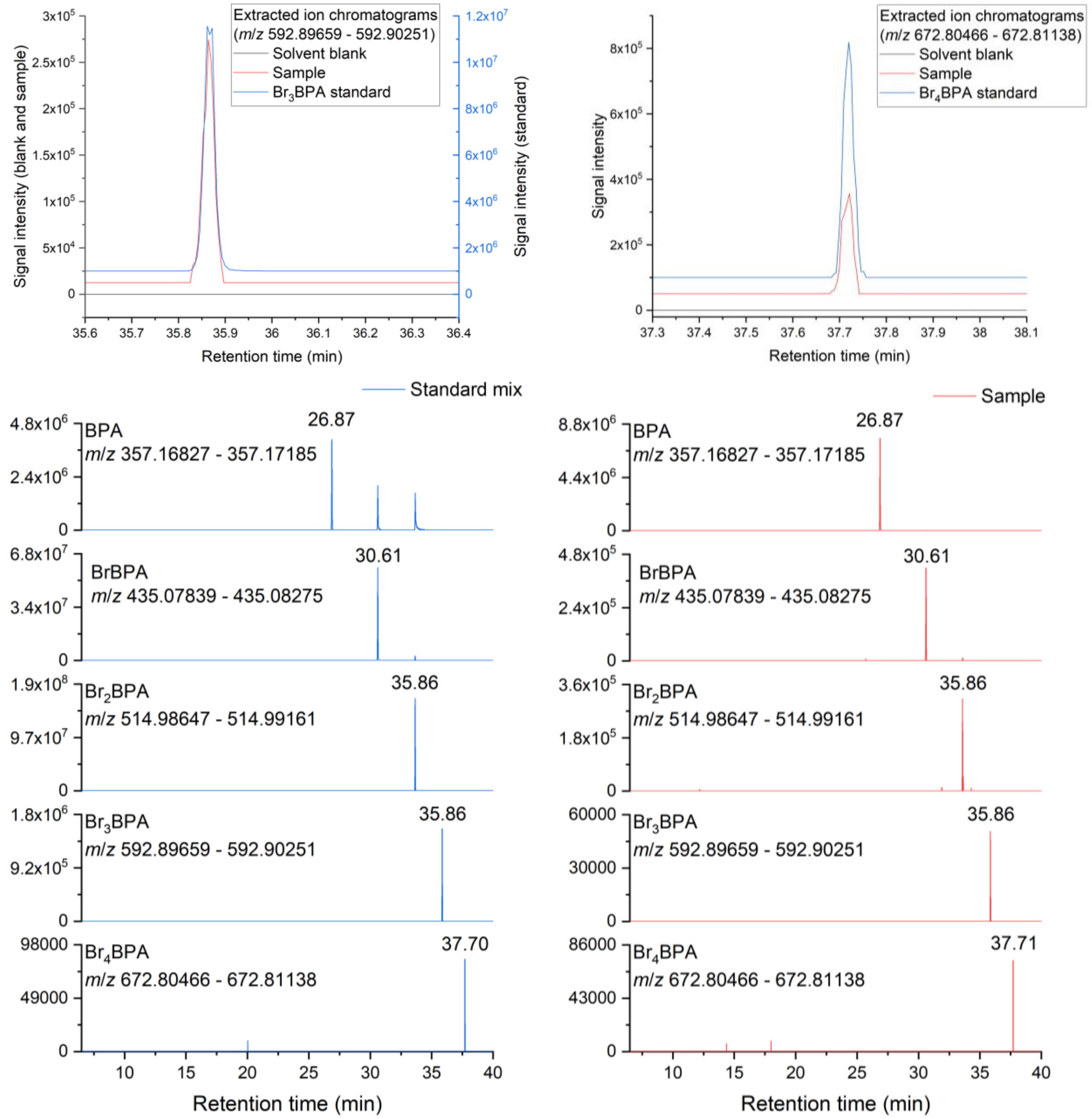

Figure S26. Proposed structure of tri- and tetrabromobisphenol A and extracted ion chromatograms of polybrominated bisphenol A. Chromatograms of synthesized standards and sample show the predicted $[M\text{-}CH_3]^+$ ions and are drawn with offsets. Fragments with Br losses were observed in chromatograms of less brominated analogues (see the standard mix, BPA).

3.17. **Tribromobisphenol A (Br3BPA)**, rt 35.86 min, GC (derivatized)

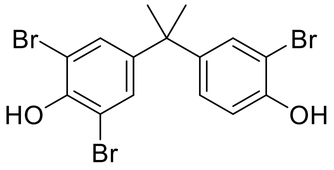

2,6-dibromo-4-(2-(3-bromo-4-hydroxyphenyl)propan-2-yl)phenol

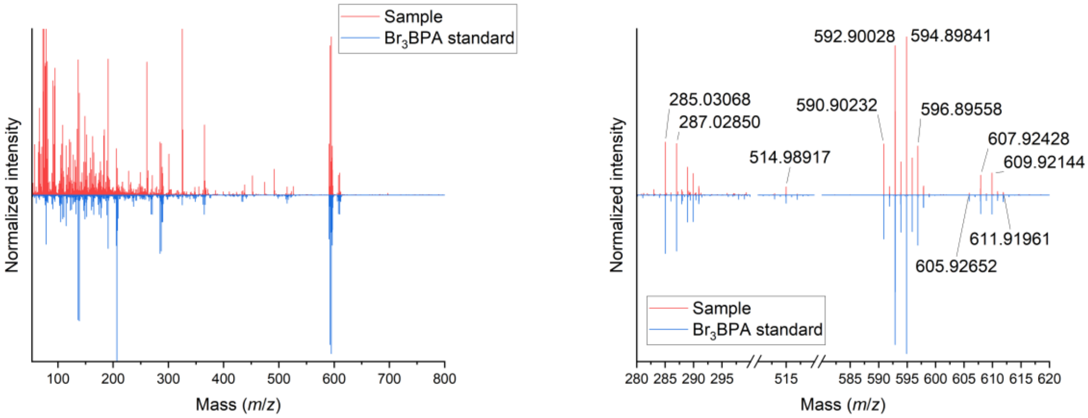

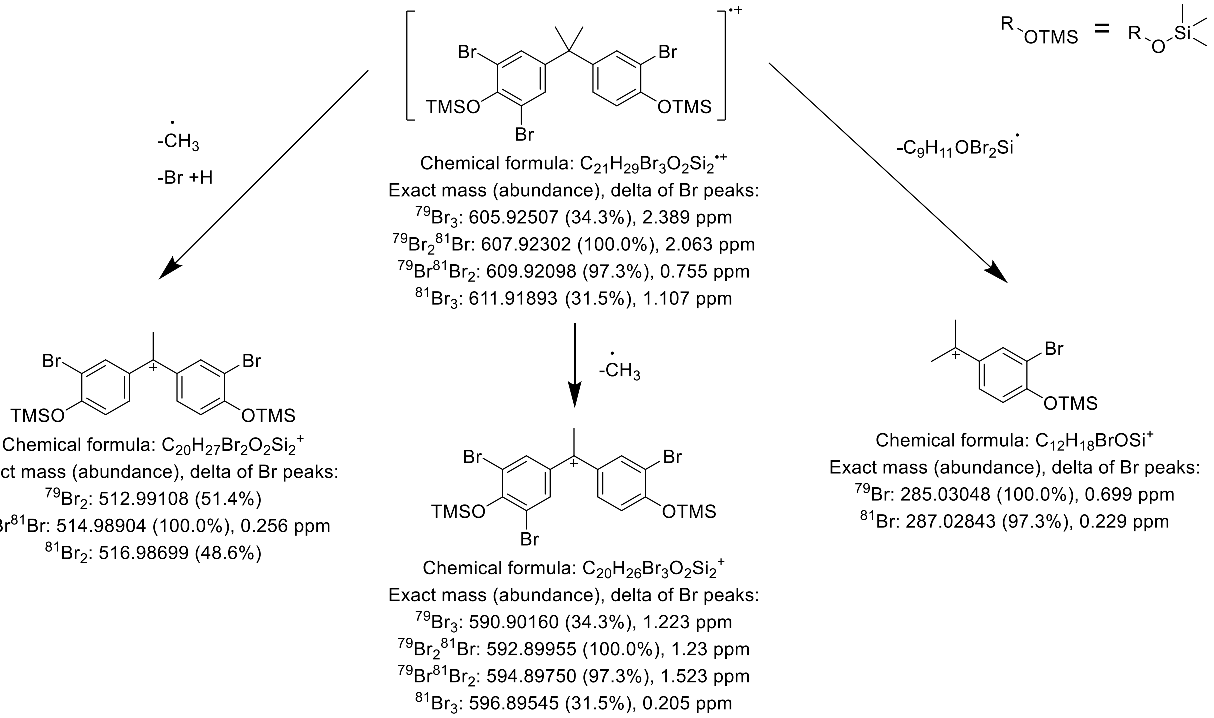

Figure S27. Proposed structure, mass spectra, identified fragments, and fragmentation pathway of tribromobisphenol A. The first spectrum contains high matrix interference due to the low abundance of target molecules. The second spectrum shows masses of the most important fragments without matrix interference.

3.18. **Tetrabromobisphenol A (Br4BPA)**, rt 37.70 min, GC (derivatized)

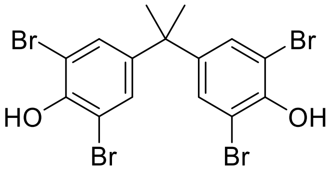

4,4'-(propane-2,2-diyl)bis(2,6-dibromophenol)

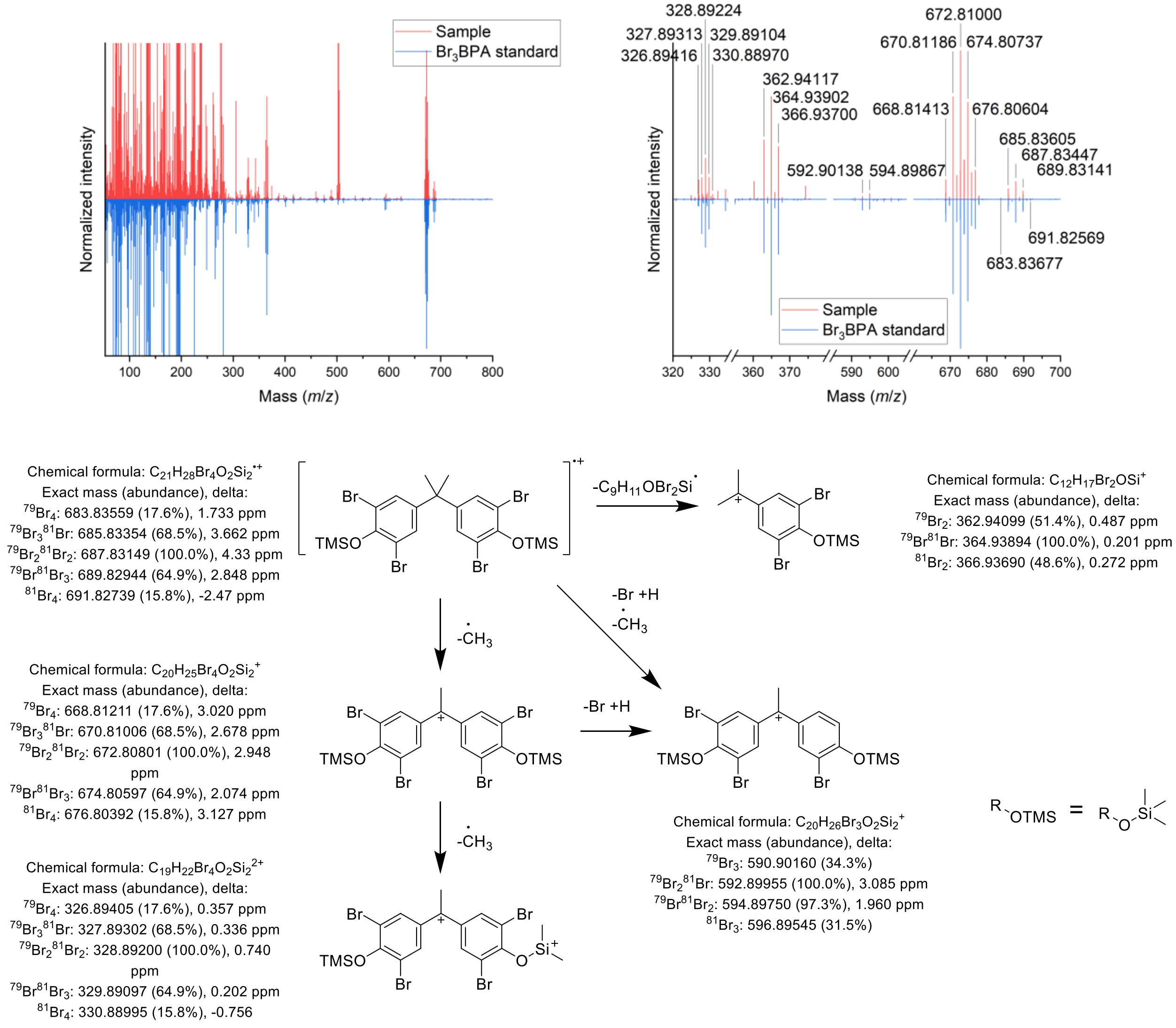

Figure S28. Proposed structure, mass spectra, identified fragments, and fragmentation pathway of tetrabromobisphenol A. The first spectrum exhibits significant matrix interference caused by the low concentration of target molecules. The second spectrum displays the masses of the most significant fragments, excluding any interference from the matrix. The structural proposition was confirmed by the spectral library.[15]

3.19. **Bisphenol A-hydroquinone ether (BPA-HQ)**, rt 37.05 min, GC (derivatized)

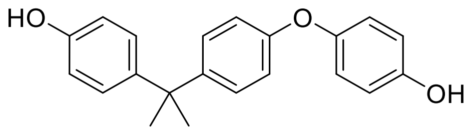

4-(2-(4-(4-hydroxyphenoxy)phenyl)propan-2-yl)phenol

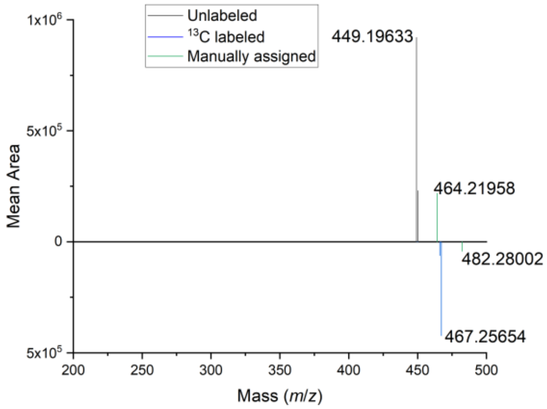

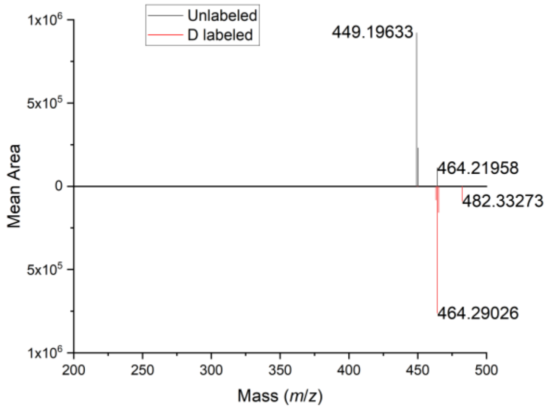

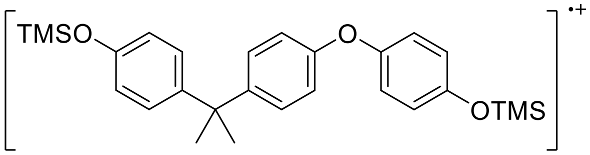

Chemical formula: $C_{27}H_{36}O_3Si_2^{\bullet +}$
Exact mass: 464.21975
Delta ppm: -0.365

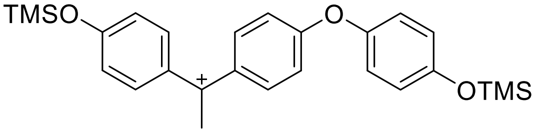

Chemical formula: $C_{26}H_{33}O_3Si_2^{+}$
Exact mass: 449.19627
Delta ppm: -0.365

Figure S29. Proposed structure, mass spectra, and identified fragments of Bisphenol A-hydroquinone ether (BPA-HQ).

3.20. **Brominated bisphenol A-hydroquinone ether isomers**, rt 38.65 min, GC (derivatized)

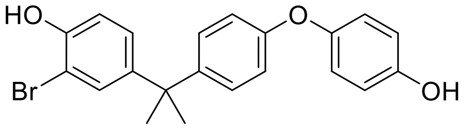

2-bromo-4-(2-(4-(4-hydroxyphenoxy)phenyl)propan-2-yl)phenol

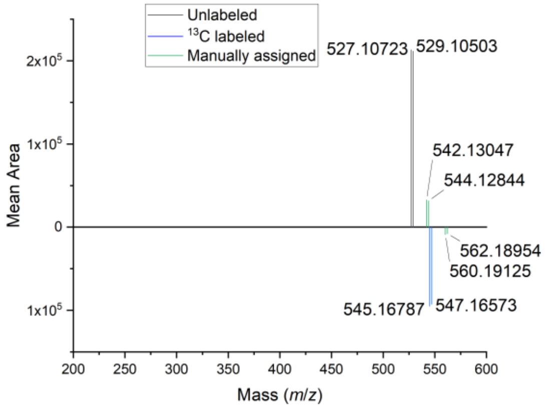

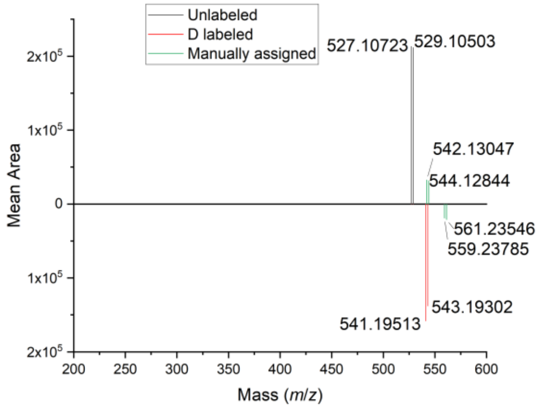

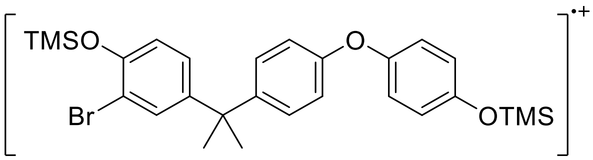

Chemical formula: $C_{27}H_{35}BrO_3Si_2^{\bullet+}$
Exact mass (abundance), delta of Br peaks:
^{79}Br: 542.13026 (100.0%), 0.377 ppm
^{81}Br: 544.12821 (97.3%), 0.419 ppm

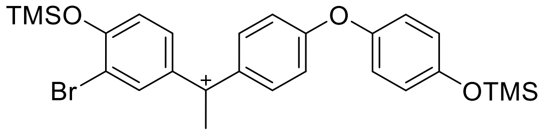

Chemical formula: $C_{26}H_{32}BrO_3Si_2^{+}$
Exact mass (abundance), delta of Br peaks:
^{79}Br: 527.10679 (100.0%), 0.841 ppm
^{81}Br: 529.10474 (97.3%), 0.547 ppm

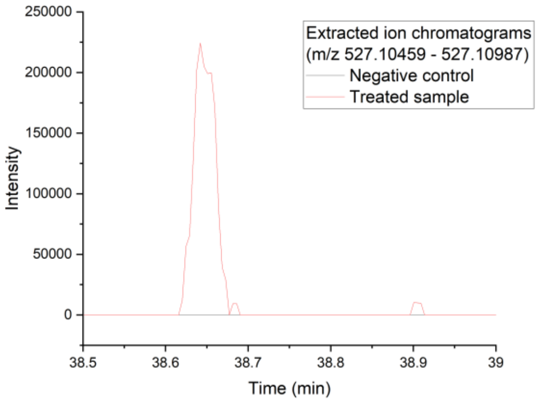

Figure S30. Proposed structure, mass spectra, identified fragments, and chromatogram of brominated bisphenol A-hydroquinone ether. Based on the shape of the peak in the extracted ion chromatogram, it could be inferred that there were one or two prominent isomers and possibly two less significant isomers. Although there are four potential isomers with brominated ortho position of phenolic oxygen, we have only illustrated one to enhance clarity.

3.21. **Bisphenol A bisulfate**, rt 4.39 min, LC in negative mode

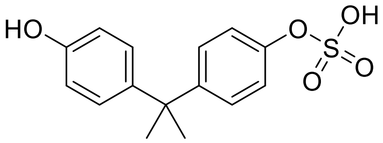

4-(2-(4-hydroxyphenyl)propan-2-yl)phenyl hydrogen sulfate

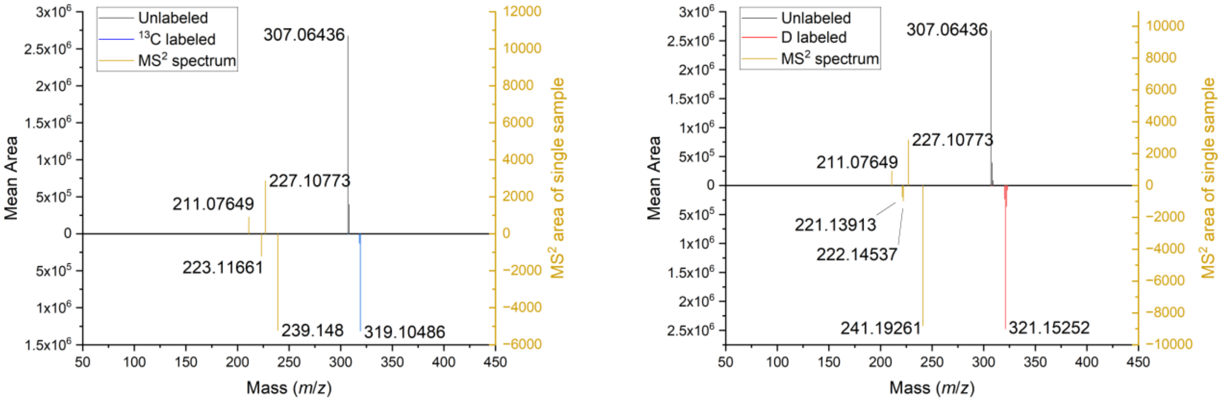

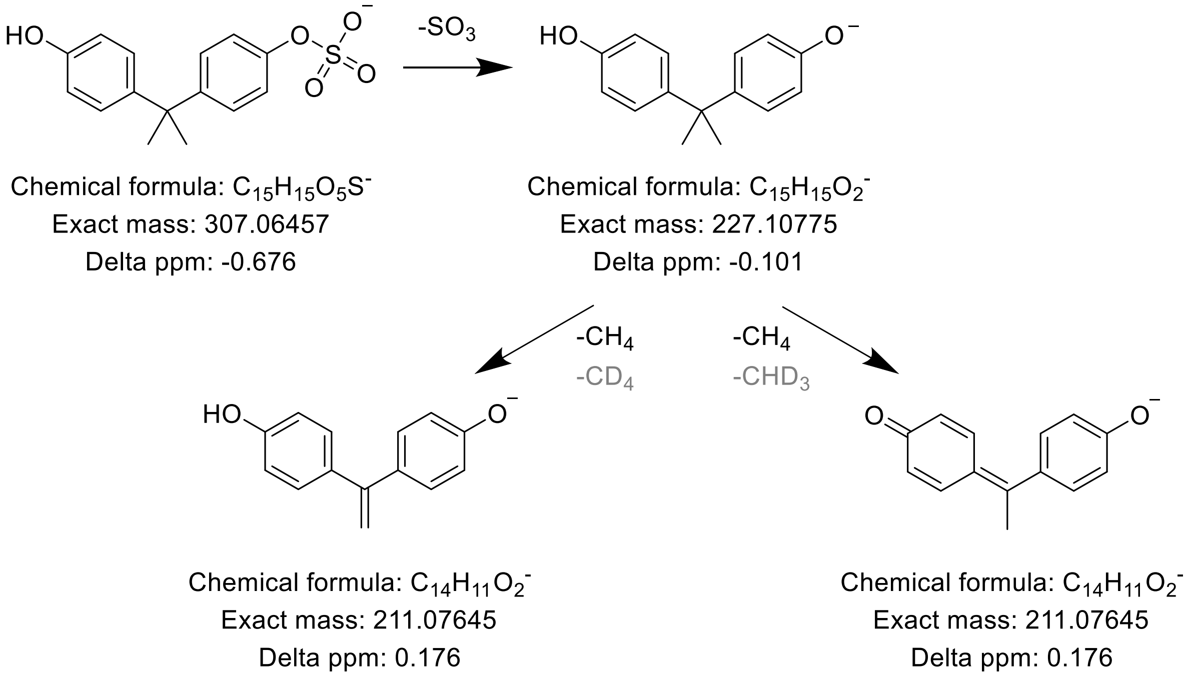

Figure S31. Proposed structure, mass spectra, and MS^2 fragmentation of bisphenol A bisulfate. MS^2 spectra (yellow) were measured with 55 eV collision energy from the *m/z* 307 precursor ion. The originally labeled phenolic D atom was lost due to its relative acidity. Therefore, the *m/z* 307 quasi-molecular ion carries a D_{14} label and two differently labeled fragments of the *m/z* 211 ions were detected, resulting from different fragmentation of the *m/z* 227 ion.

3.22. **Bromobisphenol A bisulfate**, rt 4.62 min, LC in negative mode

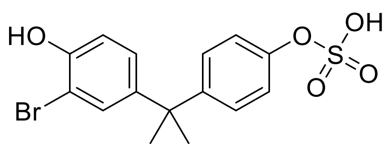

4-(2-(3-bromo-4-hydroxyphenyl)propan-2-yl)phenyl hydrogen sulfate

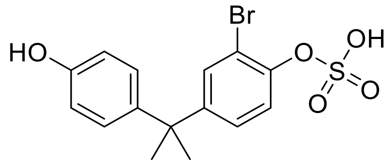

2-bromo-4-(2-(4-hydroxyphenyl)propan-2-yl)phenyl hydrogen sulfate

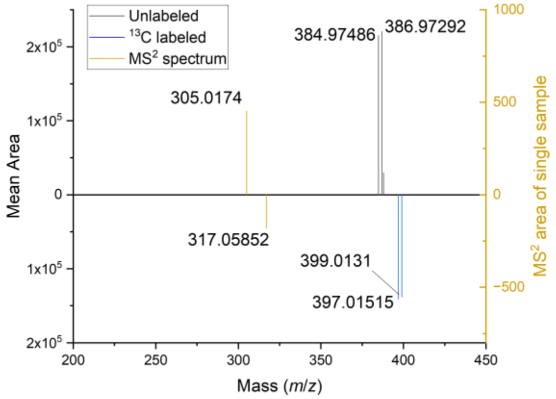

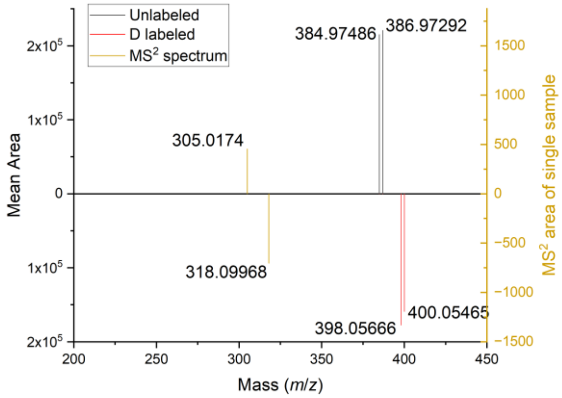

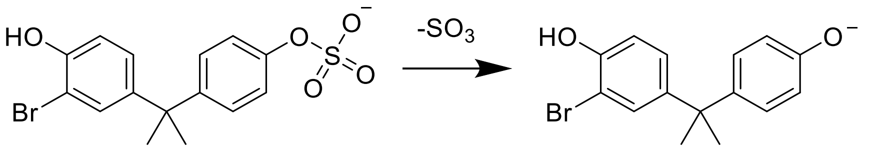

Chemical formula: $C_{15}H_{14}BrO_5S^-$
Exact mass (abundance), delta of Br peaks:
^{79}Br: 384.97508 (100.0%), -0.572 ppm
^{81}Br: 386.97303 (97.3%), -0.293 ppm

Chemical formula: $C_{15}H_{14}BrO_2^-$
Exact mass: 305.01827
Delta ppm: -2.838

Figure S32. Proposed structure, mass spectra, and MS^2 fragmentation of bromobisphenol A bisulfate. Since MS^2 spectra (yellow) were measured with 55 eV collision energy from the *m*/*z* 385 precursor ion, the fragment contained only ^{79}Br and the typical bromine M+2 peak was not observed in MS^2. The originally labeled phenolic D atom was lost due to its relative acidity. Therefore, the *m/z* 385/387 quasi-molecular ion carries a D_{13} label. As discussed before, a bromination in the ortho position is very likely. Although two such isomers exist, it is probable, that enzymatic transformation happens predominantly at the opposite ends of the molecule due to steric hindrance. This isomer was chosen for the depiction of fragmentation.

3.23. **Dibromobisphenol A bisulfate**, rt 6.13 min, LC in negative mode

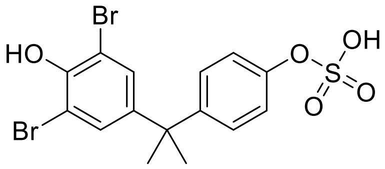

4-(2-(3,5-dibromo-4-hydroxyphenyl)propan-2-yl)
phenyl hydrogen sulfate

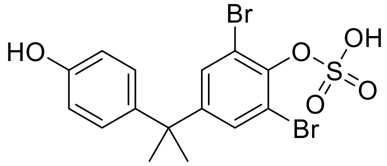

2,6-dibromo-4-(2-(4-hydroxyphenyl)propan-2-yl)
phenyl hydrogen sulfate

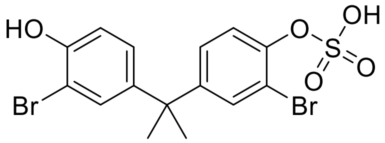

2-bromo-4-(2-(3-bromo-4-hydroxyphenyl)propan-2-yl)
phenyl hydrogen sulfate

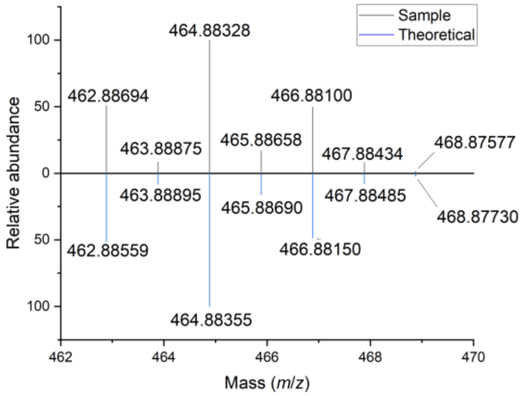

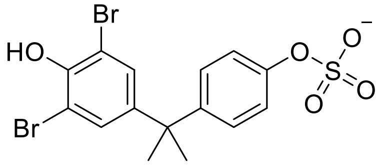

Chemical Formula: $C_{15}H_{13}Br_2O_5S^-$
Exact mass (abundance), delta of Br peaks:
$^{79}Br_2$: 462.88560 (51.4%), 2.918 ppm
$^{79}Br^{81}Br$: 464.88355 (100.0%), -0.579 ppm
$^{81}Br_2$: 466.88150 (48.6%), -1.063 ppm

Figure S33. Proposed structure and mass spectrum of dibromobisphenol A bisulfate. There are no samples with labels available because this transformation product was detected in a separate experiment. As previously mentioned, a bromination in the ortho position is highly probable. While there are three isomers, it is likely that enzymatic transformation primarily occurred at the ends of the molecule that are opposite to each other, because of steric hindrance. This particular isomer was selected to represent the quasi-molecular ion.

3.24. Additionally detected features with $^{13}C_{6n}$ label, LC in negative mode

Table S9. Features detected with X^{13}CMS with $^{13}C_{6n}$ label. *Mass was manually added as the original X^{13}CMS output detected an isotopic peak instead of base mass.

Retention time (min)	Mass (m/z)	^{13}C Label
**5.25**	361.1445	18
**5.95**	603.2748	30
**6.00**	532.9939	18
**6.31**	359.1654*	18
**6.34**	513.1892	24
**6.41**	443.0500*	18
**6.48**	531.1147*	24
**6.59**	679.3059	36
**6.77**	611.0261	24
**6.81**	569.0918	24
**7.19**	661.1531	30
**7.21**	579.1941*	30
**7.21**	606.2128*	30
**7.21**	611.2047	30
**7.21**	661.1531*	30
**7.21**	643.1426	30
**7.52**	739.0635	30
**7.52**	661.0994	30

4. Transformation products of bisphenol F

4.1. Ethoxymethylphenol, rt 11.04 min, GC (derivatized)

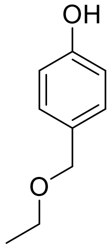

4-(ethoxymethyl)phenol

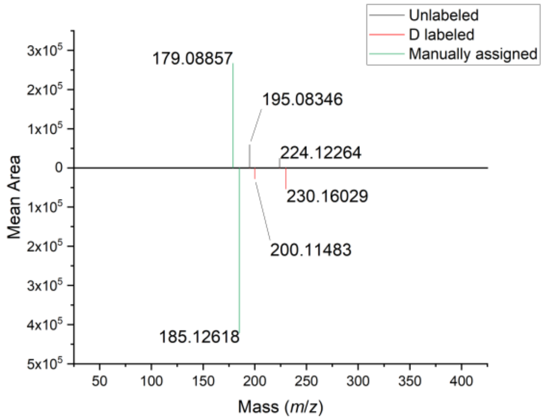

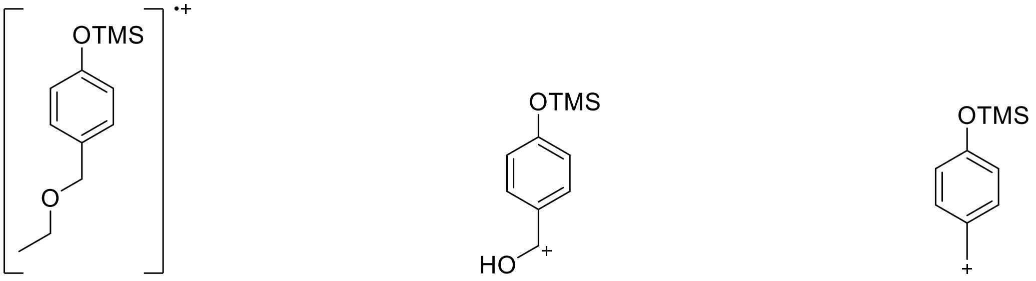

Chemical formula: $C_{12}H_{20}O_2Si^{\bullet+}$
Exact mass: 224.12271
Delta ppm: -0.303

Chemical formula: $C_{10}H_{15}O_2Si^{+}$
Exact mass: 195.08358
Delta ppm: -0.629

Chemical formula: $C_{10}H_{15}OSi^{+}$
Exact mass: 179.08867
Delta ppm: -0.303

Figure S34. Mass spectra and identified fragments of ethoxymethylphenol. The structural proposition was confirmed by spectral library.[16]

4.2. **Hydroxybenzaldehyde**, rt 12.42 min, GC (derivatized)

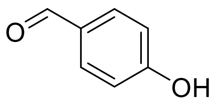

4-hydroxybenzaldehyde

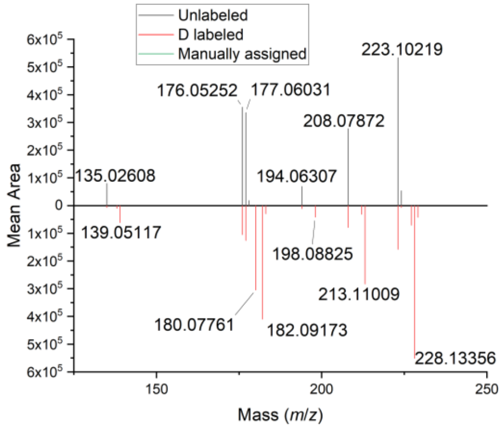

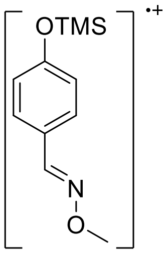

Chemical formula: $C_{11}H_{17}NO_2Si^{\bullet+}$
Exact mass: 223.10231
Delta ppm: -0.524

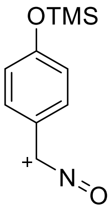

Chemical formula: $C_{10}H_{14}NO_2Si^{+}$
Exact mass: 208.07883
Delta ppm: -0.537

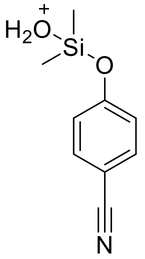

Chemical formula: $C_9H_{12}NO_2Si^{+}$
Exact mass: 194.06318
Delta ppm: -0.575

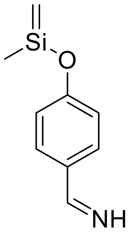

Chemical formula: $C_9H_{11}NOSi$
Exact mass: 177.06099
Delta ppm: -0.746

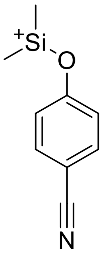

Chemical formula: $C_9H_{10}NOSi^{+}$
Exact mass: 176.05262
Delta ppm: -0.551

$C_7H_7OSi^{+}$

Chemical formula: $C_7H_7OSi^{+}$
Exact mass: 135.02607
Delta ppm: 0.090

Figure S35. Mass spectra and identified fragments of hydroxybenzaldehyde. The structural proposition was confirmed by the spectral library.[17]

Hydroxymethylphenol, rt 12.61 min, GC (derivatized)

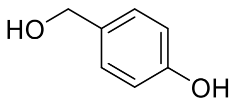

4-(hydroxymethyl)phenol

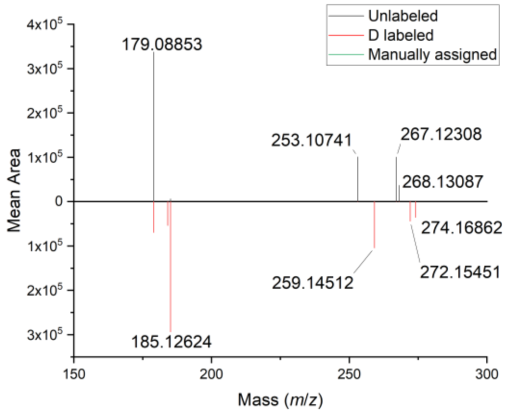

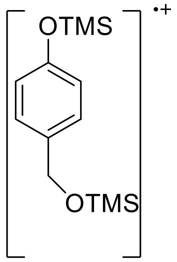

Chemical formula: $C_{13}H_{24}O_2Si_2^{\bullet+}$
Exact mass: 268.13093
Delta ppm: -0.241

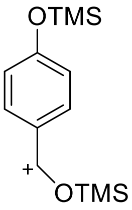

Chemical formula: $C_{13}H_{23}O_2Si_2^{+}$
Exact mass: 267.12311
Delta ppm: -0.110

$[M-CH_3]^+$

Chemical formula: $C_{12}H_{21}O_2Si_2^{+}$
Exact mass: 253.10746
Delta ppm: -0.195

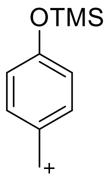

Chemical formula: $C_{10}H_{15}OSi^{+}$
Exact mass: 179.08867
Delta ppm: -0.771

Figure S36. Mass spectra and identified fragments of hydroxymethylphenol. The structural proposition was confirmed by the spectral library.[18]

4.3. **Aminomethylphenol**, rt 18.06 min, GC (derivatized)

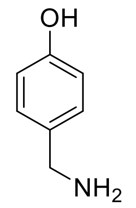

4-(aminomethyl)phenol

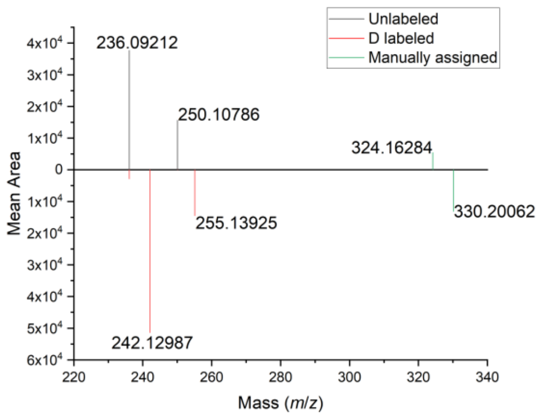

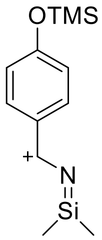

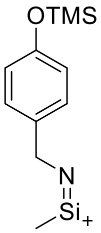

$[M-CH_3]^+$		
Chemical formula: $C_{15}H_{30}NOSi_3^+$	Chemical formula: $C_{12}H_{20}NOSi_2^+$	Chemical formula: $C_{11}H_{18}NOSi_2^+$
Exact mass: 324.16297	Exact mass: 250.10779	Exact mass: 236.09214
Delta ppm: -0.403	Delta ppm: 0.265	Delta ppm: -0.101

Figure S37. Mass spectra and identified fragments of aminomethylphenol. The structural proposition was confirmed by the spectral library.[19]

5. Transformation products of ethinylestradiol

5.1. Isomers of bromoethinylestradiol, rt 37.54 min and 37.75 min, GC (derivatized)

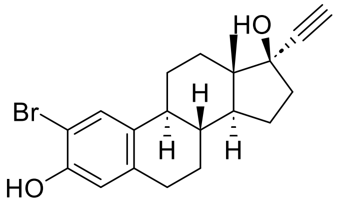

2-bromoethinylestradiol

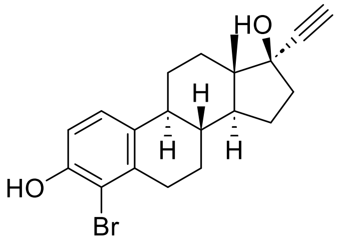

4-bromoethinylestradiol

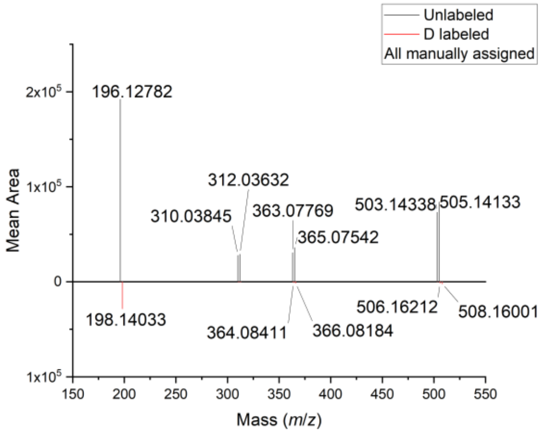

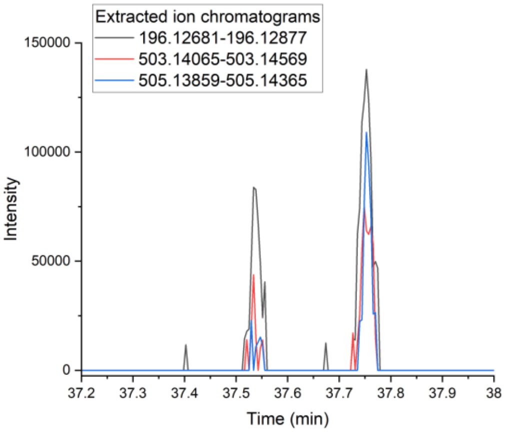

$[M{-}CH_3]^+$

Chemical formula: $C_{25}H_{36}BrO_2Si_2^+$
Exact mass (abundance), delta of Br peaks:
^{79}Br: 503.14317 (100.0%), 0.412 ppm
^{81}Br: 505.14112 (97.3%), 0.404 ppm

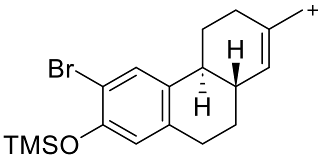

Chemical formula: $C_{18}H_{24}BrOSi^+$
Exact mass (abundance), delta of Br peaks:
^{79}Br: 363.07743 (100.0%), 0.713 ppm
^{81}Br: 365.07538 (97.3%), 0.097 ppm

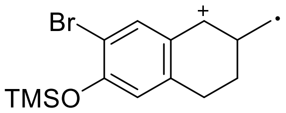

Chemical formula: $C_{14}H_{19}BrOSi^{\bullet+}$
Exact mass (abundance), delta of Br peaks:
^{79}Br: 310.03830 (100.0%), 0.465 ppm
^{81}Br: 312.03626 (97.3%), 0.195 ppm

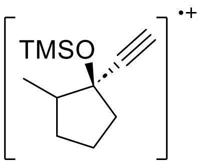

Chemical formula: $C_{11}H_{20}OSi^{\bullet+}$
Exact mass: 196.12779
Delta ppm: 0.136

Figure S38. Mass spectra, extracted ion chromatograms, and identified fragments of isomers of bromoethinylestradiol. Traces of two isomers with retention times 37.54 min and 37.75 min.

X[13]CMS only detected the feature pair *m*/*z* 196 and 198 due to the low intensity of deuterium-labeled samples. However, brominated peaks were detected in the unlabeled samples and could be matched with traces in labeled samples. A comparison with the precursor (Fig. S39) confirmed that the compound was brominated ethinylestradiol. Several labeled deuterium atoms of different fragments indicated that the bromine atom replaced one of the two deuterium atoms at the phenol ring (ortho position). Additionally, bisphenol A was also brominated in this position. Since two peaks were observed in the extraction ion chromatograms (**Fig. S38**), bromination seems to generate both possible isomers. The peak area of the *m*/*z* 196 fragment has a ratio of 2.4 to 1, suggesting a somewhat selective reaction.

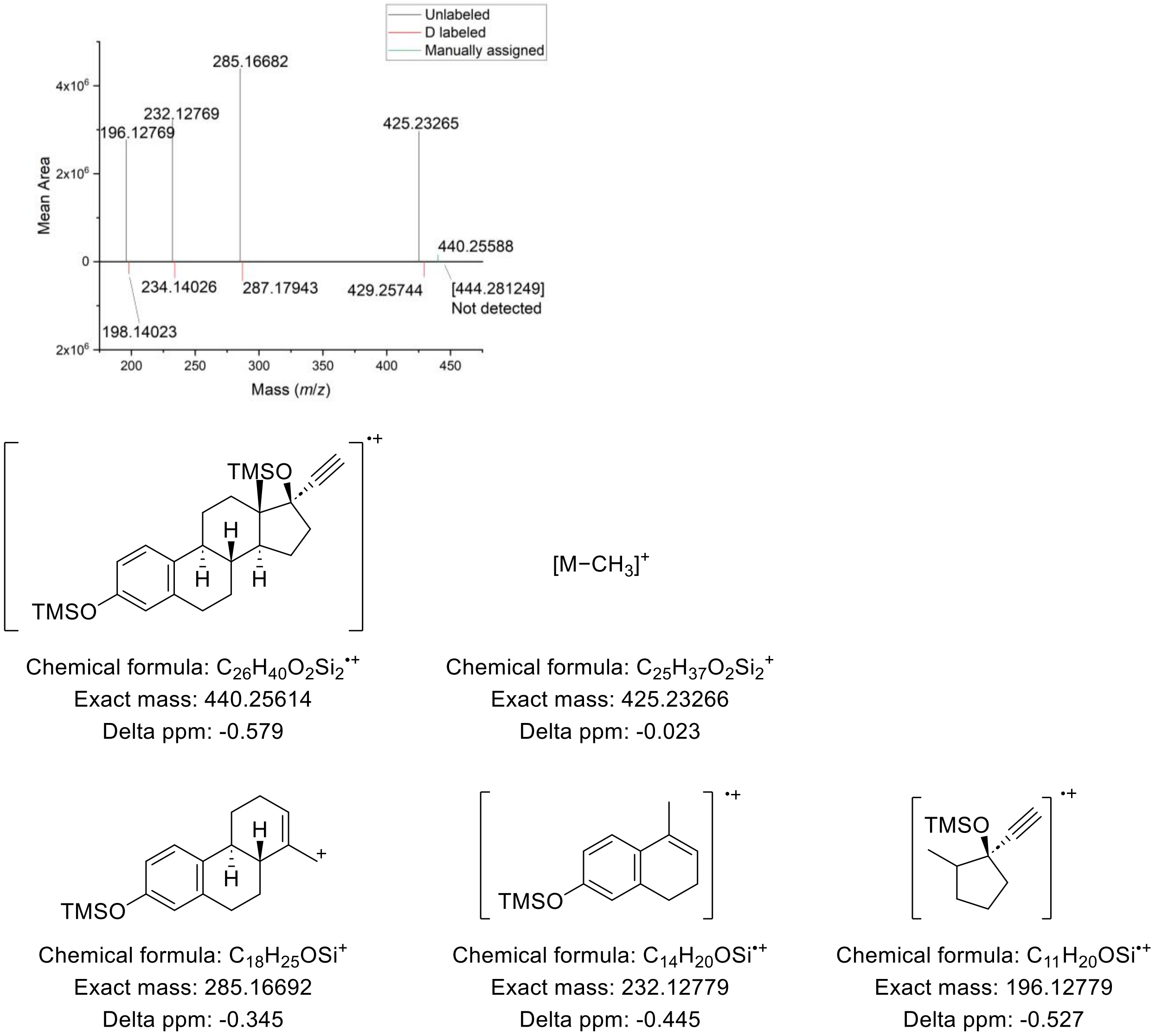

Figure S39. Spectra and proposed fragments of ethinylestradiol for comparison. The spectrum was confirmed by the spectral library.[20]

6. References

1. Hardegen, J.; Amend, G.; Wichard, T., Lifecycle-dependent toxicity and removal of micropollutants in algal cultures of the green seaweed Ulva (Chlorophyta). *J. Appl. Phycol.* **2023**. DOI: 10.1007/s10811-023-02936-x

2. OriginLab Corporation *OriginPro, Version 2024,* Northampton, MA, USA.

3. Føyn, B., Über die Sexualität und den Generationswechsel von Ulva mutabilis. *Arch. Protistenkd.* **1958,** *102*, 473-480

4. Kuhlisch, C.; Califano, G.; Wichard, T.; Pohnert, G., Metabolomics of intra- and extracellular metabolites from micro- and macroalgae using GC-MS and LC-MS. In *Protocols for Macroalgae Research*, Charrier, B.; Wichard, T.; Reddy, C. R. K., Eds. CRC Press: Boca Raton, 2018, Chapter 18.

5. Chambers, M. C.; Maclean, B.; Burke, R.; Amodei, D.; Ruderman, D. L.; Neumann, S.; Gatto, L.; Fischer, B.; Pratt, B.; Egertson, J.; Hoff, K.; Kessner, D.; Tasman, N.; Shulman, N.; Frewen, B.; Baker, T. A.; Brusniak, M. Y.; Paulse, C.; Creasy, D.; Flashner, L.; Kani, K.; Moulding, C.; Seymour, S. L.; Nuwaysir, L. M.; Lefebvre, B.; Kuhlmann, F.; Roark, J.; Rainer, P.; Detlev, S.; Hemenway, T.; Huhmer, A.; Langridge, J.; Connolly, B.; Chadick, T.; Holly, K.; Eckels, J.; Deutsch, E. W.; Moritz, R. L.; Katz, J. E.; Agus, D. B.; MacCoss, M.; Tabb, D. L.; Mallick, P., A cross-platform toolkit for mass spectrometry and proteomics. *Nat. Biotechnol.* **2012,** *30*, (10), 918-20. DOI: 10.1038/nbt.2377

6. Smith, C. A.; Want, E. J.; O'Maille, G.; Abagyan, R.; Siuzdak, G., XCMS: processing mass spectrometry data for metabolite profiling using nonlinear peak alignment, matching, and identification. *Anal. Chem.* **2006,** *78*, (3), 779-87. DOI: 10.1021/ac051437y

7. Huang, X.; Chen, Y. J.; Cho, K.; Nikolskiy, I.; Crawford, P. A.; Patti, G. J., X13CMS: global tracking of isotopic labels in untargeted metabolomics. *Anal. Chem.* **2014,** *86*, (3), 1632-9. DOI: 10.1021/ac403384n

8. Meyer, N.; Rydzyk, A.; Pohnert, G., Pronounced Uptake and Metabolism of Organic Substrates by Diatoms Revealed by Pulse-Labeling Metabolomics. *Front. Mar. Sci.* **2022,** *9*. DOI: 10.3389/fmars.2022.821167

9. Hardegen, J.; Braeutigam, P.; Abendroth, C.; Wichard, T., Bisphenol A: Quantification in Complex Matrices and Removal by Anaerobic Sludges. *Pollutants* **2021,** *1*, (4), 194-206. DOI: 10.3390/pollutants1040016

10. Hübschmann, H.-J., *Handbook of GC/MS : fundamentals and applications*. 2nd, completed rev. and updated ed.; Wiley-VCH: Weinheim, 2009.

11. McLafferty, F. W.; Tureček, F. e., *Interpretation of mass spectra*. 4th ed.; University Science Books: Mill Valley, Calif., 1993.

12. Baumeister, T. U. H.; Ueberschaar, N.; Pohnert, G., Gas-Phase Chemistry in the GC Orbitrap Mass Spectrometer. *J. Am. Soc. Mass Spectrom.* **2019,** *30*, (4), 573-580. DOI: 10.1007/s13361-018-2117-5

13. National Institute of Standards and Technology, Trimethyl[4-(1-methyl-1-methoxyethyl)phenoxy]silane. In *NIST Standard Reference Database 1A v17*, 2017

14. Wiley & Sons Ltd, Acetophenone methoxime <4-hydroxy->, mono-TMS, isomer 2. In *Biologically and Environmentally Important Organic Compounds: GCMS Library*, 2021

15. National Institute of Standards and Technology, 2,2-Bis[(4-trimethylsiloxy)phenyl]propane. In *NIST Standard Reference Database 1A v17*, 2017

16. National Institute of Standards and Technology, 4-(Ethoxymethyl)phenol, TMS. In *NIST Standard Reference Database 1A v20*, 2020

17. Wiley & Sons Ltd, Benzaldehyde <4-hydroxy->, methoxime, mono-TMS, isomer 2. In *Biologically and Environmentally Important Organic Compounds: GCMS Library*, 2021

18. National Institute of Standards and Technology, Silane, trimethyl[[p-(trimethylsiloxy)benzyl]oxy]-. In *NIST Standard Reference Database 1A v17*, 2017

19. National Institute of Standards and Technology, 4-Hydroxybenzylamine, N,N,O-tris(trimethylsilyl)-. In *NIST Standard Reference Database 1A v20*, 2020

20. National Institute of Standards and Technology, 19-Norpregna-1,3,5(10)-trien-20-yne, 3,17-bis[(trimethylsilyl)oxy]-, (17α)-. In *NIST Standard Reference Database 1A v17*, 2017
